# Targeting oncogenic FLT3 uncovers a ferroptosis vulnerability through selenocysteine recoding in acute myeloid leukaemia

**DOI:** 10.1038/s41556-026-02016-5

**Published:** 2026-08-07

**Authors:** Minhua Li, Yudan Zhu, Yuki Kageyama, Ken Furudate, Ayumi Kitano, Taotao Tan, Mengdie Feng, Jing Zhou, Tao Wang, Robert J. Taylor, Alexandra M. Stevens, Md. Abul Hassan Samee, Jeffrey A. Magee, Koichi Takahashi, Daisuke Nakada

**Affiliations:** 1 Graduate Program in Development, Disease Models and Therapeutics, Baylor College of Medicine, Houston, TX USA; 2 Department of Molecular and Human Genetics, Baylor College of Medicine, Houston, TX USA; 3 Department of Leukemia, The University of Texas MD Anderson Cancer Center, Houston, TX USA; 4 Department of Genomic Medicine, The University of Texas MD Anderson Cancer Center, Houston, TX USA; 5 Department of Integrative Physiology, Baylor College of Medicine, Houston, TX USA; 6 Graduate Program Quantitative and Computational Biosciences, Baylor College of Medicine, Houston, TX USA; 7 Dan L Duncan Comprehensive Cancer Center, Baylor College of Medicine, Houston, TX USA; 8 Veterinary Integrative Biosciences, Veterinary Medicine and Biomedical Sciences, Texas A&M University, College Station, TX USA; 9 Division of Pediatric Hematology/Oncology, Department of Pediatrics, Baylor College of Medicine, Houston, TX USA; 10 Department of Pediatrics, Division of Hematology and Oncology, Washington University School of Medicine, St. Louis, MO USA

**Keywords:** Targeted therapies, Cancer, Cell death, Translation

## Abstract

Ferroptosis, an iron-dependent form of cell death driven by lipid peroxidation, has emerged as a potential therapeutic strategy for therapy-resistant cancers. Glutathione peroxidase 4 and the selenoprotein biosynthesis pathway essential for its translation are key regulators of ferroptosis but lack effective therapeutic targeting. In a drug screening using a selenoprotein translation reporter, here we identify FMS-like tyrosine kinase 3 (FLT3) inhibitors as suppressors of selenoprotein translation that induce ferroptosis in *FLT3*-mutant acute myeloid leukaemia. Mechanistically, FLT3 inhibition disrupts selenocysteine recoding, in which a UGA stop codon is recoded as selenocysteine via the SECIS element and associated binding proteins. Notably, the antileukemic efficacy of the FLT3 inhibitor gilteritinib was markedly reduced by dietary vitamin E, which attenuated ferroptosis. This study highlights ferroptosis as a vulnerability in *FLT3*-mutant acute myeloid leukaemia and suggests that high vitamin E intake may compromise tyrosine kinase inhibitor efficacy partly by suppressing ferroptosis.

## Abstract (web summary)

Li et al. report that FLT3 inhibition impairs selenoprotein translation and induces ferroptosis to suppress *FLT3*-mutant leukaemia. This can be counteracted by dietary vitamin E, which may disrupt the efficacy of tyrosine kinase inhibitors in treatment of acute myeloid leukaemia.

## Main

Acute myeloid leukaemia (AML) is a genetically heterogeneous disease characterized by the aberrant expansion of myeloid precursor cells that evade cell death and differentiation. Among more than 70 recurrently mutated genes, FMS-like tyrosine kinase 3 (*FLT3*) is the most frequently mutated gene found in a third of patients with AML. As the most common FLT3 mutation, internal tandem duplication of *FLT3* (*FLT3*-ITD) constitutively activates tyrosine kinase activity, accounts for 25% of all AML cases and is associated with a poor prognosis^1,2^. Active FLT3 endows AML to evade cell death and differentiation through the PI3K, STAT5 and MAPK pathways^3^. The prevalence and clinical significance of *FLT3* mutations have prompted the development of small-molecule inhibitors against FLT3. Inhibitors like midostaurin and quizartinib have been approved for the treatment of patients who have been newly diagnosed with AML, and gilteritinib has been approved for relapsed or refractory AML^4–6^. Although these FLT3 inhibitors have brought additional therapeutic options to this high-risk subset of AML, they fail to achieve sustained FLT3 inhibition, and resistance to monotherapy is inevitable^7^. Resistance to FLT3 inhibition occurs through paracrine signals from the tumour microenvironment or secondary mutations in FLT3 or other pathways, most notably in the RAS–MAPK pathway^8–12^. A better understanding of how active FLT3 and the RAS–MAPK pathway co-opts cell survival mechanisms may lead to strategies to overcome FLT3-inhibitor resistance.

Selenoproteins are a set of rare (25 in humans) proteins that contain selenocysteine (Sec), often at the active site, and are characterized by two unique features of biosynthesis absent in other proteins. First, Sec lacks its dedicated aminoacyl-tRNA synthetase and is instead synthesised on tRNA^[Ser]Sec^ by a reaction series that initially aminoacylates tRNA^[Ser]Sec^ with serine. The serine is then phosphorylated and subsequently converted to selenocysteyl group, yielding Sec-tRNA^[Ser]Sec^ (refs. ^13,14^). Second, Sec is encoded by the UGA termination codon. Recoding this stop codon to Sec requires a specific hairpin structure in the 3′ untranslated region (UTR) of selenoprotein genes known as the Sec insertion sequence (SECIS). SECIS is bound by several proteins, including SECIS binding protein 2 (SECISBP2), eukaryotic elongation factor, Sec-tRNA specific (EEFSEC) and ribosomal protein L30 (RPL30)^13^. SECISBP2 is a limiting factor for selenoprotein biosynthesis that affects the differential Sec recoding efficiencies among selenoproteins^15^.

We recently identified selenophosphate synthetase 2 (*SEPHS2*) as an AML dependency regulated by an AML-specific super-enhancer^16^. Both *SEPHS2* deletion and dietary selenium restriction impair the expression of the selenoprotein glutathione peroxidase 4 (GPX4), thereby rendering AML cells sensitive to oxidative stress. A subset of AML with *FLT3* mutations was associated with high *SEPHS2* expression, suggesting a link between oncogenic *FLT3* and the selenoprotein biosynthesis pathway^16^. Low-density lipoprotein receptor-related protein 8 (LRP8) is a receptor for SELENOP, a Sec-rich extracellular protein produced in the liver that serves as the selenium carrier^17^. Deletion of *LRP8* depletes the cellular selenium pool and causes ribosome stalling during selenoprotein translation, leading to cell death^18^. Despite strong evidence supporting the potential anticancer benefits of inhibiting selenoprotein biosynthesis, small-molecule inhibitors of this pathway remain unavailable. It is also unknown whether the chemotherapies available at present impinge on this pathway as part of their anticancer effects.

Among the 25 selenoproteins, GPX4 stands out as it is the only enzyme that can reduce lipid hydroperoxides of cell membranes^19^. Accumulation of lipid peroxides triggers ferroptosis, an iron-dependent regulated cell death mechanism involved in a number of disease states, including tissue degeneration, inflammation and cancer^20–22^. The distinct mechanism of ferroptosis, compared with other cell death, provides opportunities to target cancer cells that have developed resistance to apoptosis or necroptosis. For example, GPX4-dependent suppression of ferroptosis was shown to be a vulnerability of the therapy-resistant mesenchymal cell state^23,24^ and metastatic melanoma^25^. Several compounds that inhibit or degrade GPX4 have been developed; however, most of these compounds have limited bioavailability^20^.

Motivated by the need to develop strategies to suppress the selenoprotein biosynthesis pathway for cancer therapy, we screened a panel of approved anticancer drugs with an engineered cell line that reports Sec recoding of an UGA codon. Our study identifies tyrosine kinase inhibitors (TKIs), particularly those with high activity against FLT3, as suppressors of Sec recoding that reduces GPX4 translation and cause ferroptosis in *FLT3*-mutant AML. The in vivo efficacy of FLT3 inhibition against AML was attenuated by dietary vitamin E due to its ability to suppress ferroptosis, indicating that dietary factors may affect the efficacy of targeted therapies such as TKIs.

## Results

### Drug screens identify tyrosine kinase inhibitors as suppressors of selenocysteine recoding

**Fig. 1:**
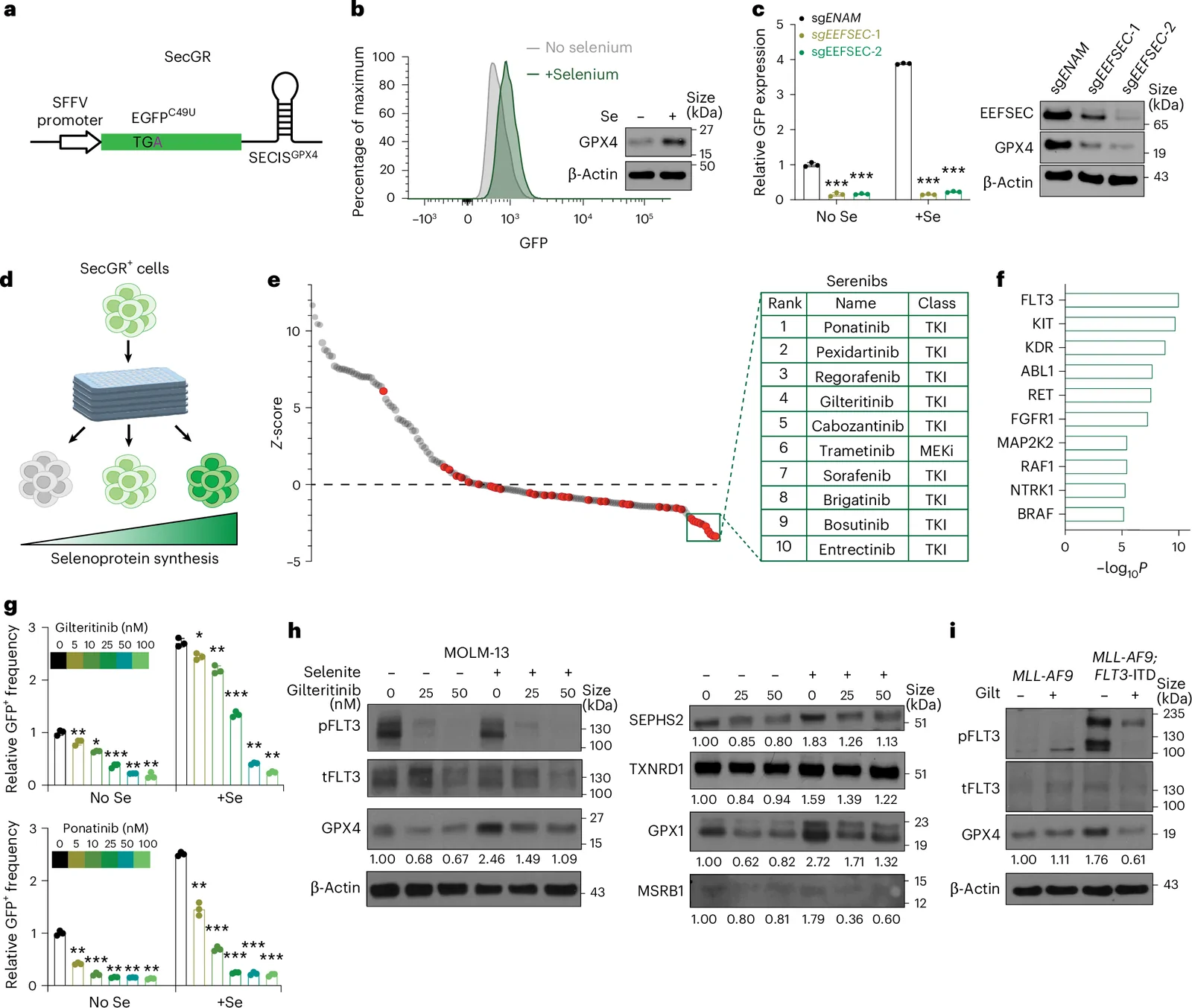
Identification of tyrosine kinase inhibitors as selenocysteine-recoding inhibitors through drug screening. **a**, Schematic of the SecGR comprising a modified EGFP with a stop codon (TGA) replacing cysteine 49 (C49U), followed by a *GPX4* SECIS element. **b**, Changes in SecGR (GFP) signal and GPX4 protein levels in a SecGR MOLM-13 cell line following treatment with 40 nM sodium selenite (Se). **c**, Changes in SecGR signal (left) and GPX4 protein (right) following CRISPR–Cas9-mediated *EEFSEC* deletion. The SecGR signals were normalized to cells treated with single guide RNA (sgRNA) targeting *ENAM* (sgENAM) in the absence of Se supplementation; sgEEFSEC, sgRNA targeting *EEFSEC*. **d**, Schematic of the drug screening performed with a SecGR MOLM-13 cell line. **e**, *Z*-scores of the 166 FDA-approved chemotherapies that increased (positive *z*-score) or decreased (negative *z*-score) GFP signal in SecGR^+^ cells (*n* = 3; left). The top ten compounds that reduced SecGR signal (serenibs) are shown (right). Red dots indicate TKIs. MEKi, MEK inhibitor. **f**, DrugEnrichr analysis against ‘Drug Repurposing Hub Target’. FLT3 was identified as the primary target of serenibs. *P* values were computed using a Fisher exact test. **g**, Effect of varying doses of gilteritinib (top) and ponatinib (bottom) on the SecGR signal in the presence or absence of 40 nM sodium selenite. Values were normalized to the ‘No Se, 0 nM’ group (treated with dimethylsulfoxide (DMSO) as the vehicle control). Exact *P* values: control versus gilteritinib, 0.003 (5 nM), 0.02 (10 nM), <0.001 (25 nM), 0.004 (50 nM) and 0.006 (100 nM); control versus gilteritinib in the presence of sodium selenite, 0.04 (5 nM), 0.006 (10 nM), <0.001 (25 nM), 0.002 (50 nM) and 0.001 (100 nM); control versus ponatinib, 0.009 (5 nM), <0.001 (10 nM) and 0.003 (25, 50, 100 nM); control versus ponatinib in the presence of sodium selenite, 0.006 (5 nM) and <0.001 (10, 25, 50, 100 nM). **c**,**g**, Data are represented as the mean ± s.d. of *n* = 3 biological replicates. *P < 0.05, **P < 0.01 and ***P < 0.001 for comparisons with the respective selenite or no selenite control; two-way analysis oof variance (ANOVA) with Dunnett’s post-hoc test for multiple comparisons. **h**, Effects of gilteritinib on the levels of phospho-FLT3 (pFLT3), and the selenoproteins GPX4, SEPHS2, TXNRD1, GPX1 and MSRB1 in MOLM-13 cells with or without sodium selenite supplementation. tFLT3, total FLT3. **i**, Effects of gilteritinib on phospho-FLT3 and GPX4 protein expression in murine AML cells transformed with *MLL-AF9* with or without *FLT3*-ITD. **c**,**h**,**i**, The relative selenoprotein levels are shown below the immunoblots. Representative immunoblot images with samples and controls run on the same gel are shown. Each of these experiments was performed multiple times. Source data

**Extended Data Fig. 1:**
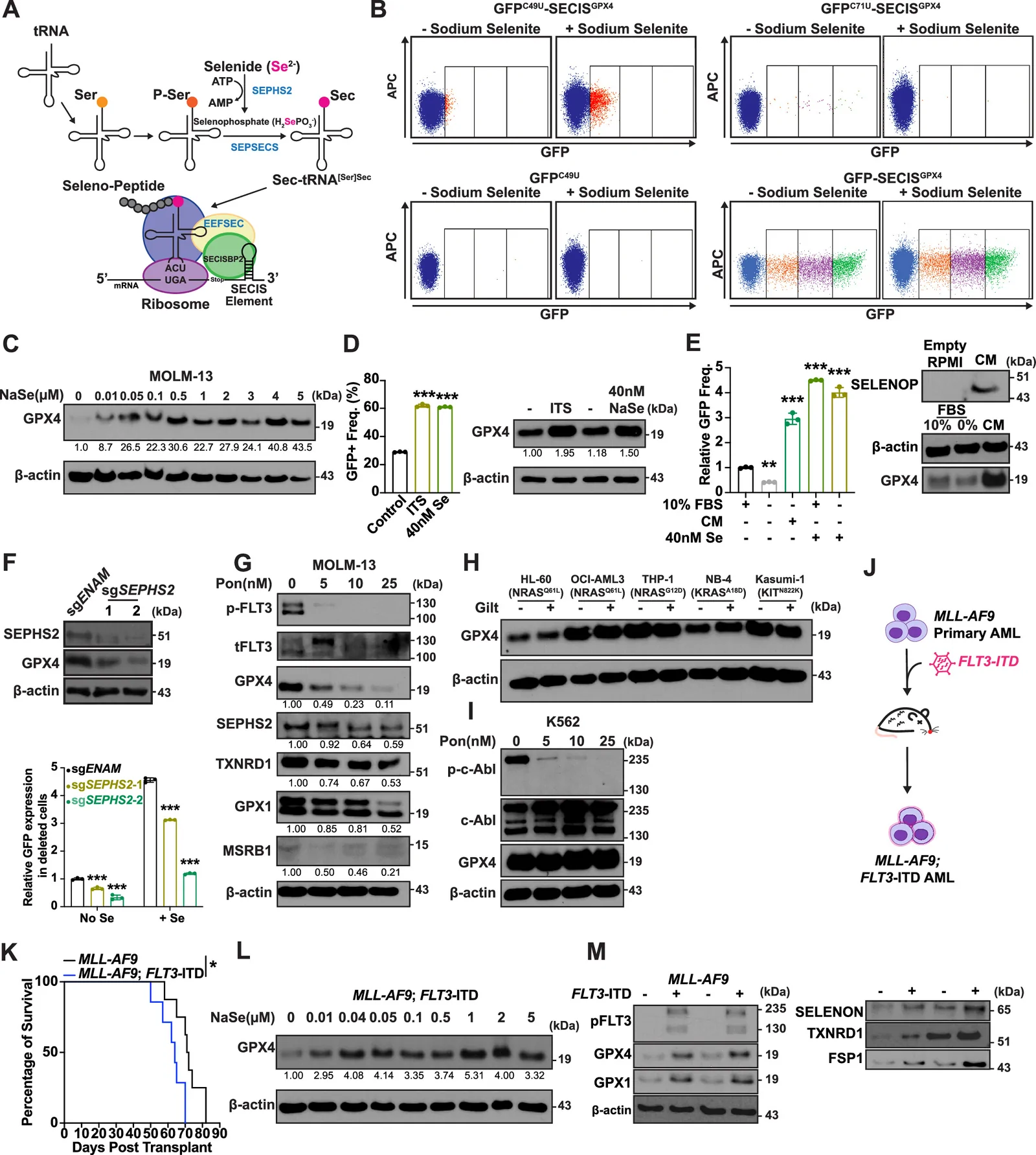
Selenium induces selenoprotein biosynthesis in AML. **a**, Schematic representation of selenoprotein biosynthesis and the enzymes involved in the process. **b**, Validation of the SecGR constructs that carry GFP variants (C49U and C71U) with or without SECIS from GPX4. **c**, GPX4 protein levels of MOLM-13 cells treated with varying concentrations of sodium selenite (Se) for 24 h. **d**, GFP signal and GPX4 expression after treating SecGR+ MOLM-13 cells with insulin-transferrin-selenium (ITS) supplement or 40 nM sodium selenite. ***P < 0.001 by one-way ANOVA with Dunnett’s test for multiple comparisons. Results were derived from biological replicates (*n* = 3). **e**, SecGR cells treated with RPMI-1640 with or without FBS, sodium selenite (Se), or conditioned media (CM) containing SELENOP. SELENOP-containing CM increased SecGR signal and GPX4 protein level. **P < 0.01, ***P < 0.001 by two-way ANOVA with Dunnett’s test for multiple comparisons. *P* value for comparison with cells cultured in regular media (left bar) are 0.003 (Se withdrawal), <0.001 (CM, 10%FBS+Se, Se only). Results were derived from biological replicates (*n* = 3). **f**, SecGR signal and GPX4 protein levels after deletion of *SEPHS2* with CRISPR–Cas9 in SecGR+ cells. ***P < 0.001 by two-way ANOVA with Dunnett’s test for multiple comparisons. Results were derived from biological replicates (*n* = 3). **g**, Selenoprotein expression in MOLM-13 cells upon ponatinib treatment for 24 h under 40 nM Se supplementation. **h**, GPX4 protein expression levels in AML cell lines without *FLT3* mutations with or without gilteritinib (50 nM) treatment. **i**, GPX4 protein expression levels in K562 cells with or without ponatinib treatment. **j**,**k**, Schematic representation of the generation (**j**) and survival curves (**k**) of *MLL-AF9*; *FLT3*-ITD murine AML compared with *MLL-AF9* AML. Survival comparison done by log-rank test (*P* = 0.01). Results were derived from biological replicates, *MLL-AF9* (*n* = 8) and *MLL-AF9*; *FLT3*-ITD (*n* = 7) AMLs. **l**, GPX4 protein levels in *MLL-AF9*; *FLT3*-ITD murine AML cells after 24-h exposure to varying concentrations of selenite ex vivo. **m**, Protein expression of selenoproteins and ferroptosis suppressor protein 1 (FSP1) in murine *MLL-AF9* or *MLL-AF9*; *FLT3*-ITD AML cells. Immunoblot images are representative images of samples ran on the same gel as controls. Each of these experiments was performed multiple times. All data are represented as the mean ± s.d. Source data

To identify clinically relevant compounds that suppress Sec recoding and selenoprotein biosynthesis, we devised a drug screening strategy that uses Sec recoding as the readout. Selenocysteine recoding was monitored by a modified green fluorescent protein (GFP) construct that carries a UGA termination codon replacing a cysteine-encoding codon, followed by a SECIS element derived from the *GPX4* 3′ UTR (Fig. 1a). We determined that replacing the cysteine residue at codon position 49 with UGA (C49U) provides the most efficient measure of Sec insertion, yielding no background GFP expression in the absence of a SECIS element (Extended Data Fig. 1a,b). This construct, termed Sec GFP reporter (SecGR), was used to generate an SecGR^+^ AML cell line from a Cas9-expressing MOLM-13 cell line^26^. Consistent with previous studies^27,28^, sodium selenite (indicated as ‘Se’) treatment of SecGR cells increased GPX4 protein expression, paralleled with increased GFP fluorescence (Fig. 1b). The addition of as little as 10 nM selenite increased the levels of GPX4 protein in MOLM-13 cells, with a plateau observed above 50 nM (Extended Data Fig. 1c). We thus added 40 nM selenite, which is similar to what the insulin-transferrin-selenium supplement for cell culture provides (37 nM selenite; Extended Data Fig. 1d). Similar increased SecGR signal and GPX4 expression were observed in SecGR cells following exposure to conditioned media enriched in SELENOP (Extended Data Fig. 1e). We and others have demonstrated that deletion of genes involved in the selenoprotein synthesis pathway—such as *SEPHS2*, *EEFSEC*, *SEPSECS* or *LRP8*—reduces GPX4 protein in AML and other cancer cells^16,18^. To further validate SecGR cells, we deleted *SEPHS2* and *EEFSEC* using CRISPR–Cas9, treated the cells with or without sodium selenite and assessed GFP expression. Both *SEPHS2* and *EEFSEC* deletion reduced baseline GFP reporter expression and attenuated its induction by sodium selenite (Fig. 1c and Extended Data Fig. 1f). These results establish that SecGR cells faithfully report Sec recoding and provide a strategy to study how AML cells regulate Sec recoding in response to stressors.

We then explored whether any of the anticancer drugs approved by the US Food and Drug Administration (FDA) impinge on Sec recoding and selenoprotein biosynthesis. We treated SecGR cells with 166 FDA-approved drugs (Fig. 1d). This allowed us to identify drugs that increase Sec recoding from baseline (termed Sec recoding activators, sereacts) as well as drugs that suppress Sec recoding (termed Sec recoding inhibitors, serenibs; Fig. 1e). Anticancer drugs that most significantly increased Sec recoding were anthracyclines, antifolates and alkylating agents (Supplementary Table 1). Although the mechanism by which these anticancer drugs increase Sec recoding requires further investigation, their identification aligns with previous studies demonstrating that they induce ferroptosis and/or oxidative stress^29–32^, possibly triggering an adaptive response to increase Sec recoding to combat ferroptosis.

Significant enrichment of TKIs among the serenibs was observed. Among the 38 TKIs in the panel, nine were within the top ten serenibs that reduced SecGR (Fig. 1e). These nine TKIs target tyrosine kinases dysregulated in haematologic malignancies such as FLT3, ALK, ABL1, KIT and CSF1R. DrugEnrichr analysis^33^ of the top ten serenibs identified a significant enrichment of inhibitors against tyrosine kinases dysregulated in haematologic malignancies, including FLT3 (*P* < 1 × 10^−10^), KIT (*P* < 1 × 10^−9^) and KDR/FLK1 (*P* < 1 × 10^−8^; Fig. 1f). Notably, four of five TKIs with known FLT3 inhibitory activity (ponatinib, gilteritinib, sorafenib and pexidartinib) were within the top ten serenibs, suggesting a role for FLT3 in Sec recoding. Both gilteritinib and ponatinib treatment reduced the SecGR signals in SecGR^+^ cells in a dose-dependent manner (Fig. 1g).

Gilteritinib and ponatinib are potent inhibitors of tyrosine kinases mutated in haematologic malignancies. Gilteritinib is approved for *FLT3*-mutant relapsed or refractory AML and ponatinib is indicated for *BCR-ABL1*^+^ leukaemias. We treated a panel of myeloid leukaemia cell lines with gilteritinib and ponatinib to determine whether they reduce Sec recoding specifically in *FLT3*-mutant or *BCR-ABL1*^+^ cells. MOLM-13 cells carry *FLT3*-ITD, whereas K562 cells carry the *BCR-ABL1* translocation. Both HL-60 and OCI-AML3 cells carry the *NRAS* Q61L mutation. The THP-1, NB4 and Kasumi-1 cell lines carry the *NRAS* G12D, *KRAS* A18D and *KIT* N822K mutations, respectively. Gilteritinib and ponatinib reduced the levels of GPX4 protein, along with several other selenoproteins, in *FLT3-*ITD^+^ MOLM-13 cells but not in the *NRAS-*, *KRAS*- or *KIT*-mutant cells (Fig. 1h and Extended Data Fig. 1g,h). Ponatinib reduced the levels of phospho-ABL1, but not GPX4, in K562 cells (Extended Data Fig. 1i). To further probe the effects of active FLT3 signalling on GPX4 protein expression, we generated murine *MLL-AF9*-induced AML cells with or without the *FLT3*-ITD mutation (Extended Data Fig. 1j,l). As shown previously^34^, *FLT3*-ITD expression accelerated the onset of leukaemogenesis by *MLL-AF9*-induced AML (Extended Data Fig. 1k). Gilteritinib reduced GPX4 protein and phospho-FLT3 in murine *MLL-AF9*; *FLT3*-ITD AML cells but these proteins were not appreciably reduced in *MLL-AF9* AML cells (Fig. 1i). Interestingly, *FLT3*-ITD expression in *MLL-AF9* AML cells increased the expression of several other selenoproteins (Extended Data Fig. 1m). These results suggest that FLT3 inhibition reduces GPX4 protein in *FLT3*-mutant AML.

### Tyrosine kinase inhibitors induce ferroptosis in acute myeloid leukaemia

**Fig. 2:**
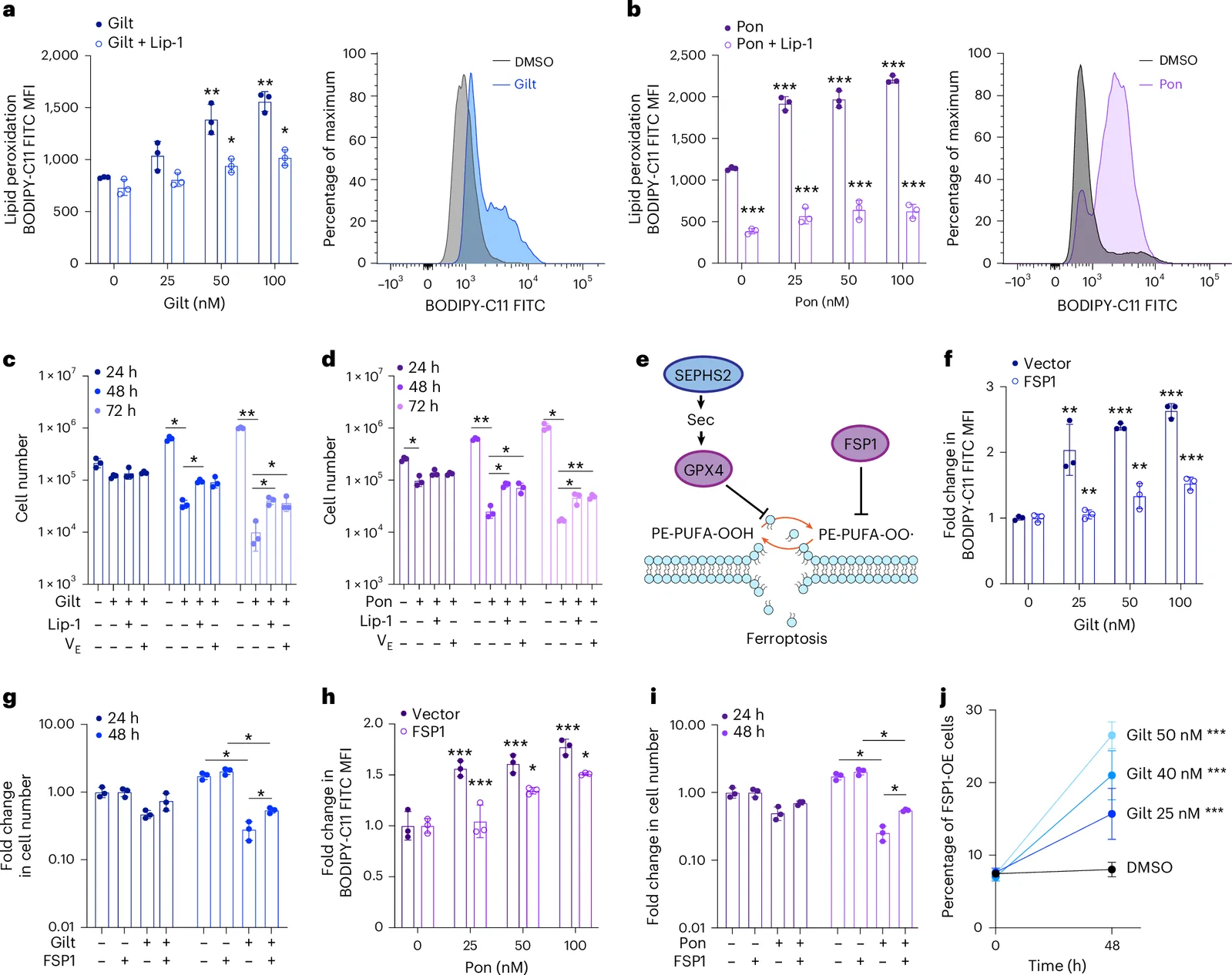
Gilteritinib and ponatinib cause ferroptosis. **a**,**b**, Effects of gilteritinib (**a**) and ponatinib (**b**) on lipid peroxidation, determined using BODIPY-C11 staining, in MOLM-13 cells in the presence or absence of liproxstatin-1 (Lip-1). Statistical comparisons of the TKI treatment group versus control and TKI + liproxstatin-1 versus TKI were made using a two-way ANOVA with Tukey’s post-hoc test for multiple comparisons. **a**, Exact *P* values: gilteritinib versus control, 0.41 (25 nM), 0.01 (50 nM) and 0.002 (100 nM); gilteritinib plus liproxstatin-1 versus gilteritinib, 0.95 (0 nM), 0.32 (25 nM), 0.03 (50 nM) and 0.01 (100 nM). **b**, *P* < 0.001 for all ponatinib versus control and ponatinib versus ponatinib plus liproxstatin-1 comparisons. **c**,**d**, Effects of gilteritinib (**c**) and ponatinib (**d**) with or without liproxstatin-1 (Lip-1) or vitamin E (V_E_) on the growth of MOLM-13 cells. Statistical comparisons were performed using a two-way ANOVA with Tukey’s post-hoc test (gilteritinib) and false detection rate method of the Benjamini–Hochberg test (ponatinib) for multiple comparisons. **c**, *P* = 0.22, 0.61, 0.22, 0.02. 0.04, 0.12, 0.002, 0.04 and 0.04 for bars 2–4, 6–8 and 10–12, respectively. **d**, *P* =0.008, 0.17, 0.08, 0.001, 0.02, 0.04, 0.008, 0.04 and 0.005 for bars 2–4, 6–8 and 10–12, respectively. **e**, Schematic of two pathways (GPX4 and FSP1) that suppress ferroptosis. PE, phosphatidylethanolamine. **f**,**h**, Effects of *FSP1* expression on the levels of BODIPY-C11 signals induced by gilteritinib (**f**) and ponatinib (**h**). Values were normalized to those of control treatment (0 nM, treatment with DMSO) of vector- or FSP1-expressing cells. Statistical comparisons were performed using a two-way ANOVA adjusted for multiple comparisons with Tukey’s post-hoc test. **f**, Vector-expressing cells treated with gilteritinib versus control (0 nM), *P* = 0.003 (25 nM) and <0.001 (50 and 100 nM); *FSP1*-expressing cells treated with gilteritinib versus vector-expressing cells, *P* = 0.99 (0 nM), 0.003 (25 nM), 0.001 (50 nM) and *P* < 0.001 (100 nM). **h**, Vector-expressing cells treated with ponatinib versus control (0 nM), *P* < 0.001 (25, 50 and 100 nM); *FSP1*-expressing cells versus vector-expressing cells, *P* = 0.99 (0 nM), <0.001 (25 nM) and 0.03 (50 and 100 nM). **g**,**i**, Effect of *FSP1* expression on the growth of MOLM-13 cells treated with or without 50 nM gilteritinib (**g**) or 25 nM ponatinib (**i**). Statistical comparisons were performed using a two-way ANOVA with Holm–Šídák’s post-hoc test for multiple comparisons. The same control group of DMSO-treated cells expressing vector or FSP1 was utilized in both panels. **g**, *P* > 0.99, *P* = 0.24 and *P* = 0.39 for bars 2–4, respectively; and *P* = 0.0463 for bars 6–8. **i**, *P* = 0.3311, 0.3311, 0.265, 0.0428, 0.0404 and 0.0428 for bars 2–4 and 6–8, respectively. **j**, Competitive culture assays using MOLM-13 cells with or without *FSP1* overexpression (FSP1-OE) treated with the indicated concentrations of gilteritinib. Two-way ANOVA with Dunnett’s post-hoc test for multiple comparisons. **a**–**d**,**f**–**j**, Data are represented as the mean ± s.d. of *n* = 3 biological replicates. *P < 0.05, **P < 0.01, ***P < 0.001. FITC, fluorescein isothiocyanate; gilt, gilteritinib; Lip-1, liproxstatin-1; MFI, mean fluorescence intensity; pon, ponatinib. Source data

**Extended Data Fig. 2:**
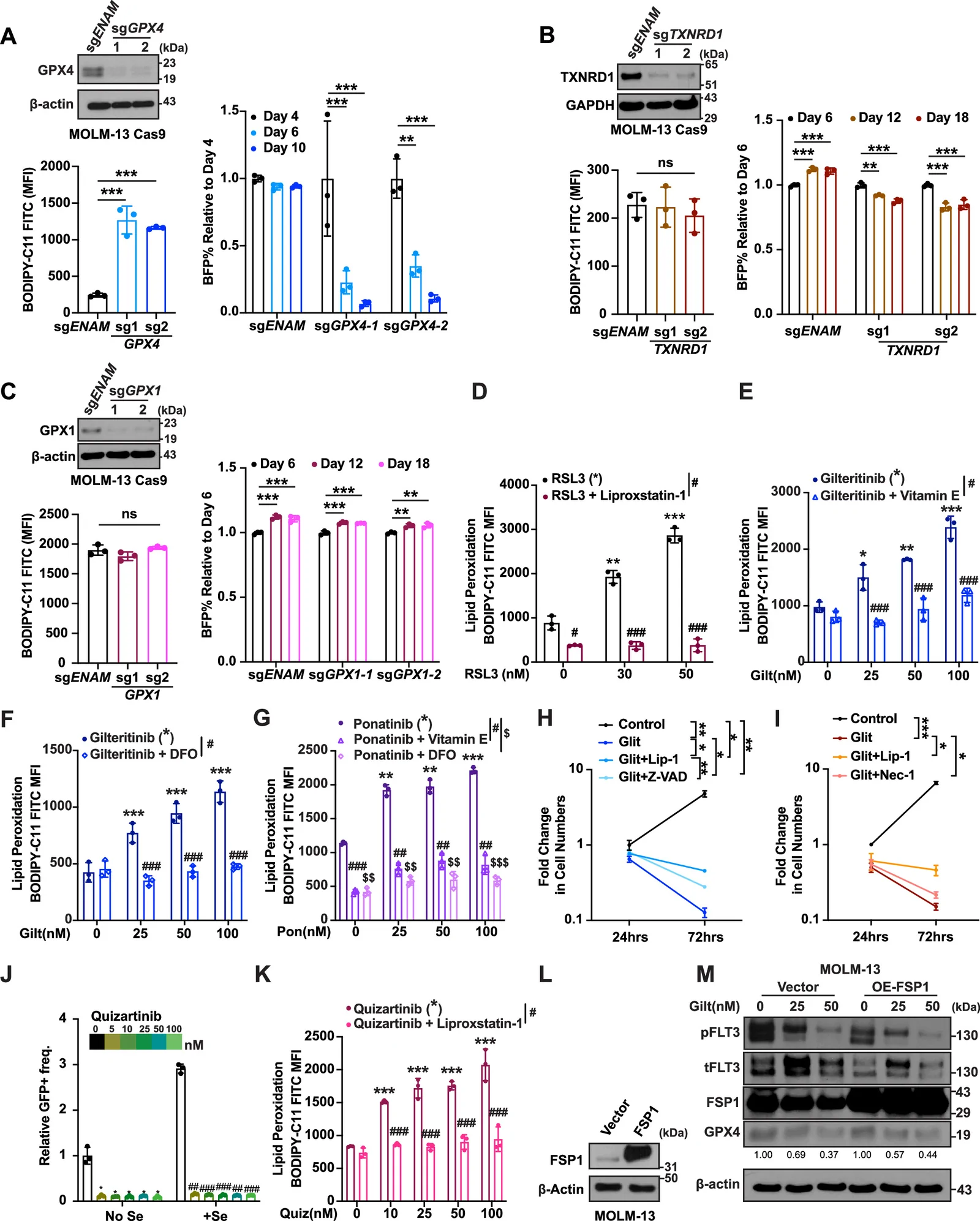
TKIs induce ferroptosis in AML. **a**–**c**, The effect of deleting GPX4 (**a**), *TXNRD1* (**b**) or *GPX1* (**C**) in Cas9-expressing MOLM-13 cells. Immunoblotting confirmed reduced protein expression of deleted genes. Lipid peroxidation levels via BODIPY-C11 (MFI) of cells with gene deletion. Competitive growth assay to trace the changes in deleted (BFP + ) and undeleted (BFP-) cell ratio after gene deletion. Bars indicate differences relative to Day 4 (**a**) or Day 6 (**b**,**c**) BFP positivity for each sgRNA. Statistical analysis for Bodipy-C11 measurements were done by comparing each specific sgRNA to the control (sg*ENAM*) using one-way ANOVA with Dunnett test for multiple comparisons (***P < 0.001). Statistical analyses for BFP tracing assay were done using two-way ANOVA with Tukey’s post-hoc test for multiple comparisons (**P < 0.01; and ***P < 0.001). *P* values for BODIPY-C11y are (**a**) *GPX4* sg1 and sg2 (*P* < 0.001); (**b**) *TXNRD1* sg1 (*P* = 0.98), sg2 (*P* = 0.67); (**c**) *GPX1* sg1 (*P* = 0.82), sg2 (*P* = 0.97). *P* value for BFP tracing assays are (**a**) sg*ENAM*: Day 6 (*P* = 0.88), Day 10 (*P* = 0.89); sg*GPX4*-1: Day 6 and Day 10 (*P* < 0.001); sg*GPX4*-2: Day 6 (*P* = 0.002), Day 10 (*P* < 0.001). (**b**) sg*ENAM*: Day 12, Day 18 (*P* < 0.001); sg*TXNRD1*-1: Day 12 (*P* = 0.003), Day 18 (*P* < 0.001); sg*TXNRD1*-2: Day 12 and Day 18 (*P* < 0.001). (**c**) sg*ENAM*: Day 12 and Day 18 (*P* < 0.001); sg*GPX1*-1: Day 12 and Day 18 (*P* < 0.001); sg*GPX1*-2: Day 12 (*P* = 0.004), Day 18 (*P* = 0.003). Results were derived from biological replicates (*n* = 3). **d**–**g**, Lipid peroxidation levels induced by RSL-3 (**d**), gilteritinib (**e**,**f**) or ponatinib (**g**) were suppressed by 1 µM of liproxstatin-1 (Lip-1; **d**), 1 µM of vitamin E (V_E_) (**e**,**g**) or 10 µM of deferoxamine (DFO; **f**,**g**). **P < 0.01; and ***P < 0.001 by two-way ANOVA comparing RSL-3, gilteritinib or ponatinib treated and untreated. ^#^ and ^$^*P* < 0.05; ^##^ and ^$$^*P* < 0.01; and ^###^ and ^$$$^*P* < 0.001 by two-way ANOVA comparing with or without Lip-1 (**e**) vitamin E (**e**,**g**) or DFO (**f**,**g**). Two-way ANOVAs performed with Tukey’s post-hoc test to adjust for multiple comparisons. *P* values for control versus RSL-3 (**d**) are 30 nM (*P* = 0.003), 50 nM (*P* < 0.001); RSL-3 vs RSL-3 plus Lip-1 are 0 nM (*P* = 0.02), 30 nM and 50 nM (*P* < 0.001); control versus gilteritinib (**e**) are 25 nM (*P* = 0.02), 50 nM (*P* = 0.002), 100 nM (*P* < 0.001); gilteritinib vs gilteritinib plus V_E_: 0 nM (*P* = 0.21), 25 nM, 50 nM and 100 nM (*P* < 0.001); (**f**) all comparisons indicating *** are *P* < 0.001; control versus ponatinib (**g**) are 25 nM (*P* = 0.008), 50 nM (*P* = 0.010), 100 nM (*P* < 0.001); ponatinib versus ponatinib plus V_E_ are 0 nM (*P* < 0.001), 25 nM (*P* = 0.010), 50 nM (*P* = 0.003), 100 nM (*P* = 0.010); ponatinib versus ponatinib plus DFO are 0 nM (*P* = 0.003), 25 nM (*P* = 0.006), 50 nM (*P* = 0.008), 100 nM (*P* < 0.001). Results were derived from biological replicates (*n* = 3). **h**,**i**, MOLM-13 cells were treated with DMSO (control), gilteritinib, gilteritinib plus liproxstatin-1 (Lip-1), or gilteritinib plus Z-VAD (**h**) or plus necrostatin-1 (Nec-1; **i**) and assessed for viability through fold change in cell number overtime. *P < 0.05; **P < 0.01; and ***P < 0.001 by two-way ANOVA with Tukey’s (**h**) or Benjamini, Krieger and Yekutieli (**i**) post-hoc test for multiple comparison. *P* values for comparison in **h** for 24 h are control versus gilteritinib (*P* = 0.12), control versus gilteritinib plus Lip-1 (*P* = 0.23), control versus gilteritinib plus Z-VAD (*P* = 0.44), gilteritinib versus gilteritinib plus Lip-1 (*P* = 0.02), gilteritinib versus gilteritinib plus Z-VAD (*P* = 0.33), gilteritinib plus Lip-1 versus gilteritinib plus Z-VAD (*P* = 0.91); for 72 h are control vs gilteritinib (*P* = 0.008), control versus gilteritinib plus Lip-1 (*P* = 0.01), control versus gilteritinib plus Z-VAD (*P* = 0.009), gilteritinib versus gilteritinib plus Lip-1 (*P* = 0.01), gilteritinib versus gilteritinib plus Z-VAD (*P* = 0.03), gilteritinib plus Lip-1 versus gilteritinib plus Z-VAD (*P* = 0.002). *P* values for comparison in **i** for 24 h are control versus gilteritinib (*P* = 0.006), control versus gilteritinib plus Lip-1 (*P* = 0.05), control versus gilteritinib plus Nec-1 (*P* = 0.01), gilteritinib versus gilteritinib plus Lip-1 (*P* = 0.48), gilteritinib versus gilteritinib plus Nec-1 (*P* = 0.50), gilteritinib plus Lip-1 versus gilteritinib plus Nec-1 (*P* = 0.62); for 72 h are control vs gilteritinib, control versus gilteritinib plus Lip-1, and control versus gilteritinib plus Nec-1 (*P* < 0.001), gilteritinib versus gilteritinib plus Lip-1 (*P* = 0.03), gilteritinib versus gilteritinib plus Nec-1 (*P* = 0.03), gilteritinib plus Lip-1 versus gilteritinib plus Nec-1 (*P* = 0.04). Results were derived from biological replicates (*n* = 3). **j**, Changes in SecGR signal upon quizartinib (quiz) treatment, with or without 40 nM selenite. *P < 0.05; ** and ^##^*P* < 0.01; and *** and ^###^*P* < 0.001 by two-way ANOVA with Dunnett’s post-hoc test comparing quizartinib treated and untreated. *P* values for control versus quizartinib are 0.03 (5 nM), 0.04 (10 nM), 0.03 (25 nM), 0.04 (50 nM) and 0.02 (100 nM). *P* values for control versus quizartinib in the presence of sodium selenite are 0.001 (5 nM), 0.001 (50 nM) and <0.001 (10, 25 and 100 nM). Results were derived from biological replicates (*n* = 3). **k**, Lipid peroxidation levels induced in MOLM-13 cells with quizartinib treatment and rescued by 1 µM Lip-1. *** / ^###^*P* < 0.001 by two-way ANOVA with Tukey’s post-hoc test were used for multiple comparisons adjustments. Results were derived from biological replicates (*n* = 3). **l**, Confirmation of FSP1 protein expression in MOLM-13 cells transduced with a *FSP1*-expression retroviral vector. **m**, Changes in GPX4 protein levels in MOLM-13 cells transduced with vector or *FSP1*-expressing construct with or without gilteritinib treatment. Immunoblot images are representative images of samples ran on the same gel as controls. Each of these experiments was performed multiple times. All data are represented as the mean ± s.d. Source data

Consistent with the ability of TKIs to reduce GPX4 protein levels, gilteritinib and ponatinib significantly increased lipid peroxides, as measured by BODIPY-C11 staining of MOLM-13 cells (Fig. 2a,b). The increased BODIPY-C11 signal indicative of ferroptosis was suppressed by liproxstatin-1, vitamin E and the iron chelator deferoxamine (Fig. 2a,b and Extended Data Fig. 2a–g). If the anti-leukemic effects of the TKIs are at least partially through ferroptosis, then ferroptosis inhibitors should ameliorate the toxicity of the TKIs. Consistent with this idea, the ferroptosis inhibitors liproxstatin-1 and vitamin E partially rescued the attenuated survival of AML cells after gilteritinib or ponatinib treatment, whereas apoptosis or necroptosis inhibitors only modestly rescued gilteritinib sensitivity (Fig. 2c,d and Extended Data Fig. 2h,i). Quizartinib, another TKI approved for newly diagnosed *FLT3*-ITD AML, also reduced Sec recoding, as measured by SecGR and increased lipid peroxidation (Extended Data Fig. 2j,k).

### Ferroptosis evasion renders *FLT3*-mutant AML resistant to TKIs

Recent studies have established FSP1 as a GPX4-independent mechanism to suppress ferroptosis^35,36^ (Fig. 2e). Our findings that TKIs reduce GPX4 and cause ferroptosis suggest that *FSP1* expression may alleviate ferroptotic cell death caused by TKIs. *FSP1* overexpression indeed reduced lipid peroxidation and rendered AML cells partially resistant to gilteritinib and ponatinib (Fig. 2f–i and Extended Data Fig. 2l) despite the levels of GPX4 protein remaining suppressed by FLT3 inhibition (Extended Data Fig. 2m).

The ability of *FSP1* to render AML partially resistant to gilteritinib provided an opportunity to test whether gilteritinib treatment selects for cells that are resistant to ferroptosis. We thus performed a competitive culture assay in which a small population (8%) of MOLM-13 cells overexpressing *FSP1* were co-cultured with cells without *FSP1* overexpression and treated with gilteritinib. The gilteritinib treatment exerted a selective pressure on *FSP1*-overexpressing cells in a dose-dependent manner, such that the *FSP1*-overexpressing cells expanded approximately threefold following gilteritinib treatment (Fig. 2j). These results establish ferroptosis as one of the mechanisms by which TKIs suppress *FLT3*-mutant AML and that ameliorating ferroptosis confers resistance to TKI therapies.

### On-target inhibition of FLT3 by tyrosine kinase inhibitors causes ferroptosis

**Fig. 3:**
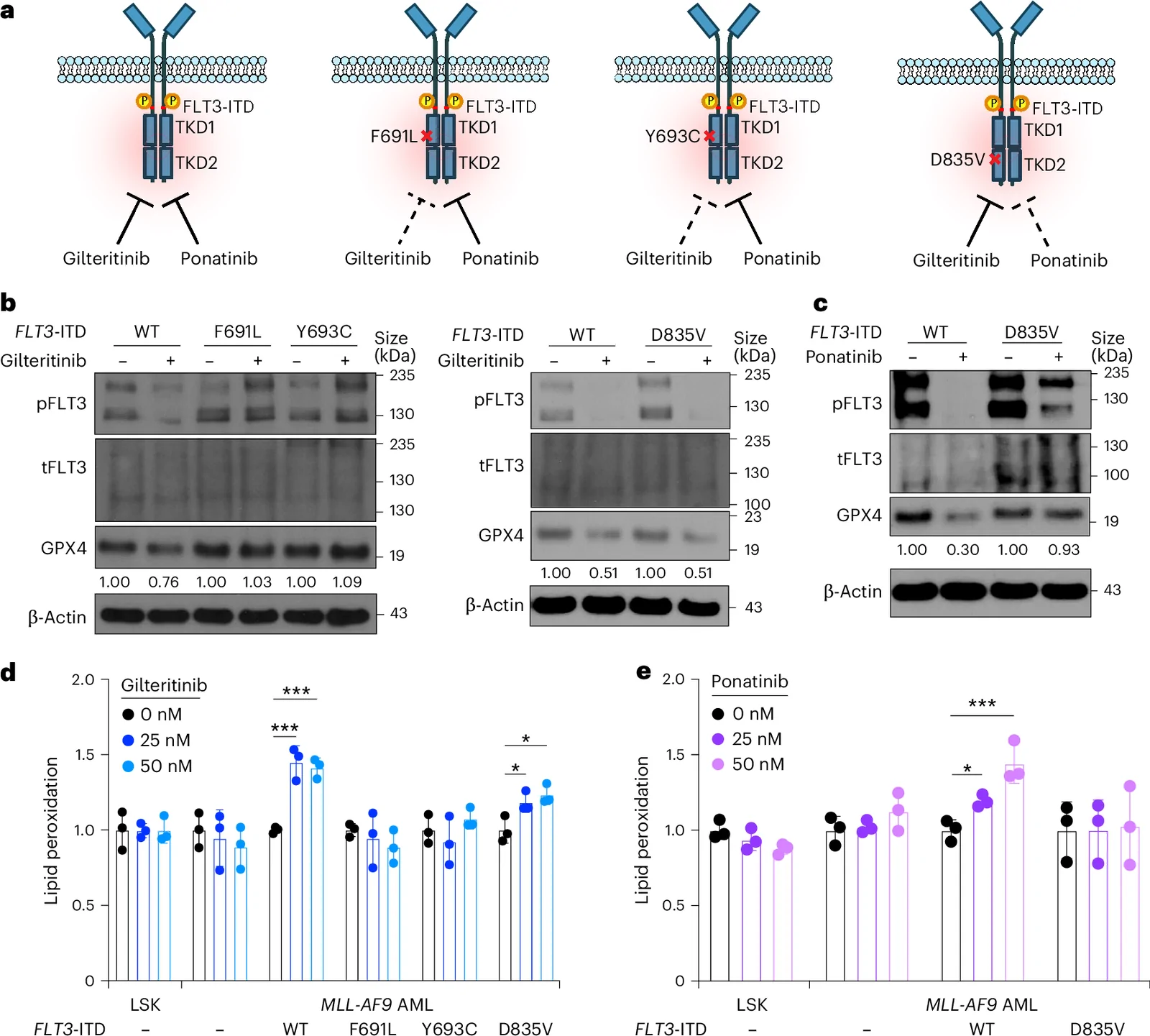
Tyrosine kinase inhibitors act on FLT3 to induce ferroptosis. **a**, Schematic of the FLT3 mutations and their clinically reported responses to TKIs. **b**, Effect of gilteritinib (50 nM) treatment on GPX4 protein expression in murine AML cells expressing the indicated *FLT3-*ITD variants. **c**, Effects of ponatinib (50 nM) treatment on GPX4 protein expression in murine AML cells expressing the indicated *FLT3-*ITD with or without the D835V mutation. **b**,**c**, Representative immunoblot images with samples and controls run on the same gel are shown. Each of these experiments was performed multiple times. The relative GPX4 levels are shown below the immunoblots. **d**,**e**, Levels of lipid peroxidation in haematopoietic progenitor cells (LSK) and murine AML cells induced by *MLL-AF9* with or without the indicated *FLT3*-ITD constructs following treatment with gilteritinib (**d**) or ponatinib (**e**). Lipid peroxidation is represented as the BODIFY-C11 mean fluorescence intensity (MFI) normalized to the control treatment (0 nM, DMSO treatment) of each cell type. **d**, Drug treatment versus DMSO (0 nM): LSK cells, *P* = 0.99 (25 and 50 nM); *MLL-AF9*, *P* = 0.5 (25 nM) and 0.31 (50 nM); *MLL-AF9;* *FLT3*-ITD, *P* < 0.001 (25 and 50 nM); *MLL-AF9;* *FLT3*-ITD^F691L^, *P* = 0.50 (25 nM) and 0.30 (50 nM); *MLL-AF9;* *FLT3*-ITD^Y693C^, *P* = 0.56 (25 and 50 nM); *MLL-AF9;* *FLT3*-ITD^D835V^*, P* = 0.03 (25 nM) and 0.01 (50 nM). **e**, LSK cells, *P* = 0.37 (25 nM) and 0.17 (50 nM); *MLL-AF9*, *P* = 0.75 (25 nM) and 0.16 (50 nM); *MLL-AF9;* *FLT3*-ITD, *P* = 0.01 (25 nM) and *P* < 0.001 (50 nM); *MLL-AF9;* *FLT3*-ITD^D835V^, *P* = 0.98 (25 nM) and 0.90 (50 nM). All data are represented as the mean ± s.d. of *n* = 3 biological replicates. Statistical comparisons were performed using a two-way ANOVA with Holm-Šídák’s post-hoc test for multiple comparisons; *P < 0.05, ***P < 0.001. Source data

**Extended Data Fig. 3:**
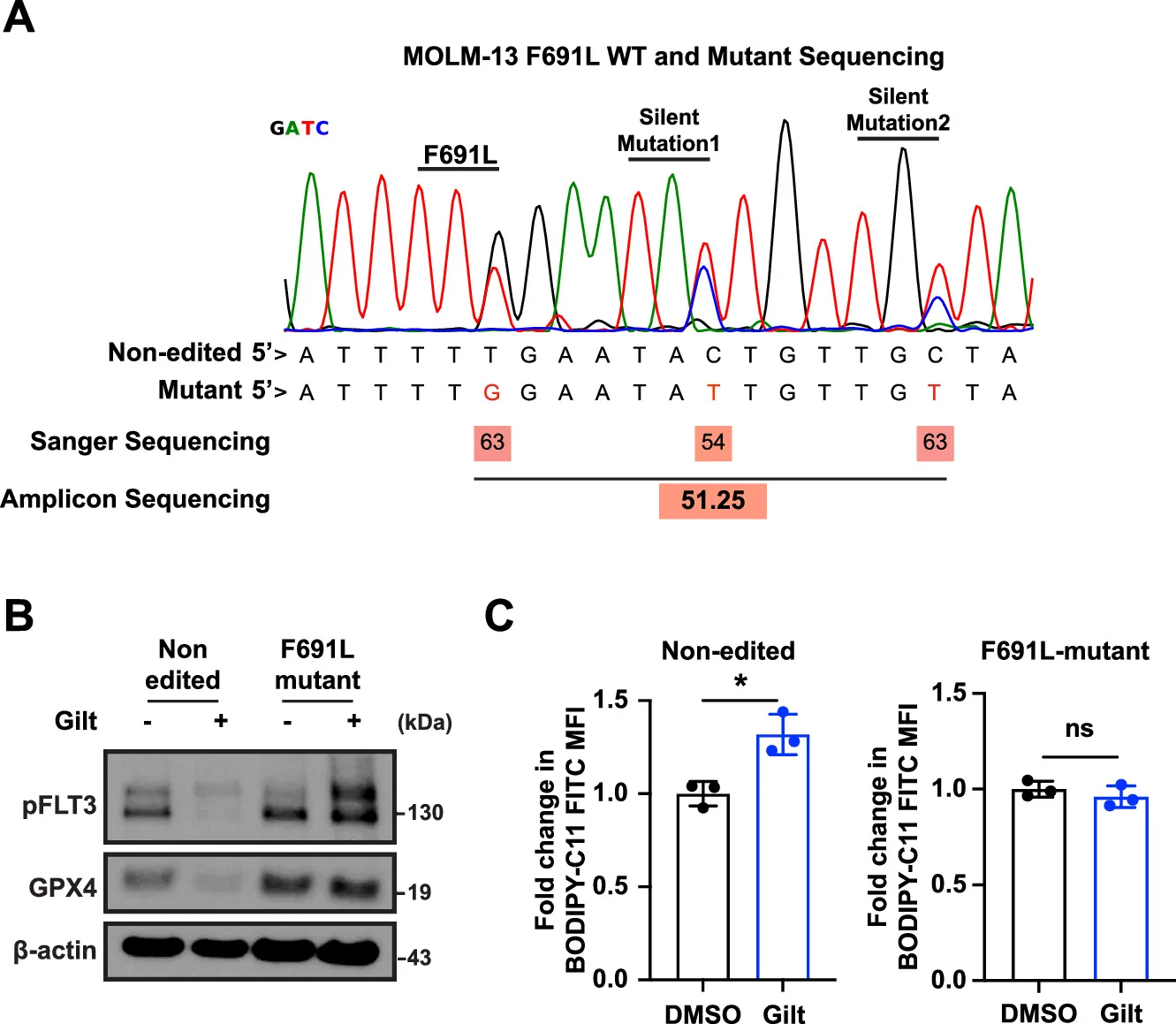
TKIs act on FLT3 to induce ferroptosis. **a**, *FLT3*-ITD^F691L^ MOLM-13 cells were generated using CRISPR-mediated homology-dependent repair (HDR). Sanger sequencing chromatograms and quantification of editing frequency by Sanger and high-throughput sequencing validating the generation of *FLT3*-ITD^F691L^ MOLM-13 cells. The numbers below the nucleotide sequence indicate the variant allele frequency. We introduced two additional silent mutations located in the sgRNA binding site of the HDR template (c.2145C>T and c.2151C>T) to prevent recutting by Cas9. **b**, Immunoblotting of non-edited and *FLT3*-ITD^F691L^ cells treated with gilteritinib (Gilt, 25 nM) showing reduced phospho-FLT3 and GPX4 in non-edited but not in *FLT3*-ITD^F691L^ cells. **c**, Lipid peroxidation measurement using BODIPY-C11 in non-edited and *FLT3*-ITD^F691L^ cells after Gilt (25 nM) treatment. *P < 0.05 by using unpaired, two-tailed Student’s *t*-test. *P* values for comparison to DMSO (control) are 0.01 (non-edited) and 0.38 (*FLT3*-ITD^F691L^). Results were derived from biological replicates (*n* = 3). Immunoblot images are representative images of samples ran on the same gel as controls. Each of these experiments was performed multiple times. All data are represented as the mean ± s.d. Source data

The results from a panel of myeloid leukaemia cell lines suggested that *FLT3*-mutant AML depends on FLT3 activity to maintain GPX4 protein and suppress ferroptosis. However, it remains possible that TKIs induce ferroptosis by acting on off targets. To firmly establish that the TKIs we identified as serenibs act on FLT3 to reduce Sec recoding, we generated AML cells carrying clinically reported *FLT3* mutations that confer resistance to TKIs. *FLT3* mutations in the activation loop, such as D835V/Y confer resistance to the type II FLT3 inhibitors ponatinib, sorafenib, quizartinib and pexidartinib (but not gilteritinib), whereas gatekeeper mutations such as F691L, Y693C and D698N confer partial resistance to the type I FLT3 inhibitor gilteritinib as well as type II inhibitors^37–39^ (Fig. 3a). We thus generated murine AML models co-expressing *MLL-AF9* and *FLT3*-ITD with or without the activation loop or gatekeeper mutations. Gilteritinib reduced FLT3 phosphorylation of wild-type and the mutant D835V *FLT3*-ITD but not the F691L or Y693C mutants (Fig. 3b and Extended Data Fig. 3b). Ponatinib reduced FLT3 phosphorylation of wild-type *FLT3*-ITD but only modestly affected the D835V mutant (Fig. 3c). Thus, these cell models of AML recapitulate the clinically reported effects of *FLT3* mutations on TKI resistance.

We then assessed the effects of the FLT3 inhibitors on GPX4. As shown in Fig. 3b, gilteritinib reduced GPX4 in *FLT3*-ITD and *FLT3*-ITD^D835V^ cells but not *FLT3*-ITD^F691L^ or *FLT3*-ITD^Y693C^ cells. Ponatinib reduced GPX4 protein in *FLT3*-ITD-expressing AML cells but not *FLT3*-ITD^D835V^-expressing cells (Fig. 3c). Consistent with the finding that the expression of GPX4 protein is reduced by gilteritinib in *FLT3*-ITD but not *FLT3* wild-type *MLL-AF9* AML cells (Fig. 1i), gilteritinib increased lipid peroxidation in *MLL-AF9;* *FLT3-*ITD AML cells but not *MLL-AF9* AML cells or untransformed haematopoietic progenitor (lineage^−^Sca-1^+^c-kit^+^, LSK) cells (Fig. 3d). The effect of gilteritinib on lipid peroxidation was abolished by the F691L and Y693C mutations but not D835V, consistent with the observation that these former mutations render *FLT3*-ITD resistant to gilteritinib (Fig. 3d). Ponatinib also increased lipid peroxidation in *MLL-AF9;* *FLT3-*ITD but not *MLL-AF9* AML or LSK cells, and this effect was abolished by the *FLT3*-ITD^D835V^ mutation (Fig. 3e). To model FLT3-inhibitor resistance in a more physiological setting, we introduced a FLT3 F691L mutation in the *FLT3*-ITD allele of MOLM-13 cells using CRISPR-mediated homology-dependent repair. *FLT3*-ITD^F691L^ MOLM-13 cells were resistant to gilteritinib, retained FLT3 phosphorylation and were protected from lipid peroxidation after gilteritinib treatment (Extended Data Fig. 3a–c). Thus, *FLT3* mutations that confer TKI resistance also render cells insensitive to the effects of TKIs in reducing GPX4 protein and inducing lipid peroxides. These results establish that gilteritinib and ponatinib act on oncogenic FLT3 to reduce GPX4 expression and cause ferroptosis.

### FLT3 inhibition suppresses GPX4 protein translation

**Extended Data Fig. 4:**
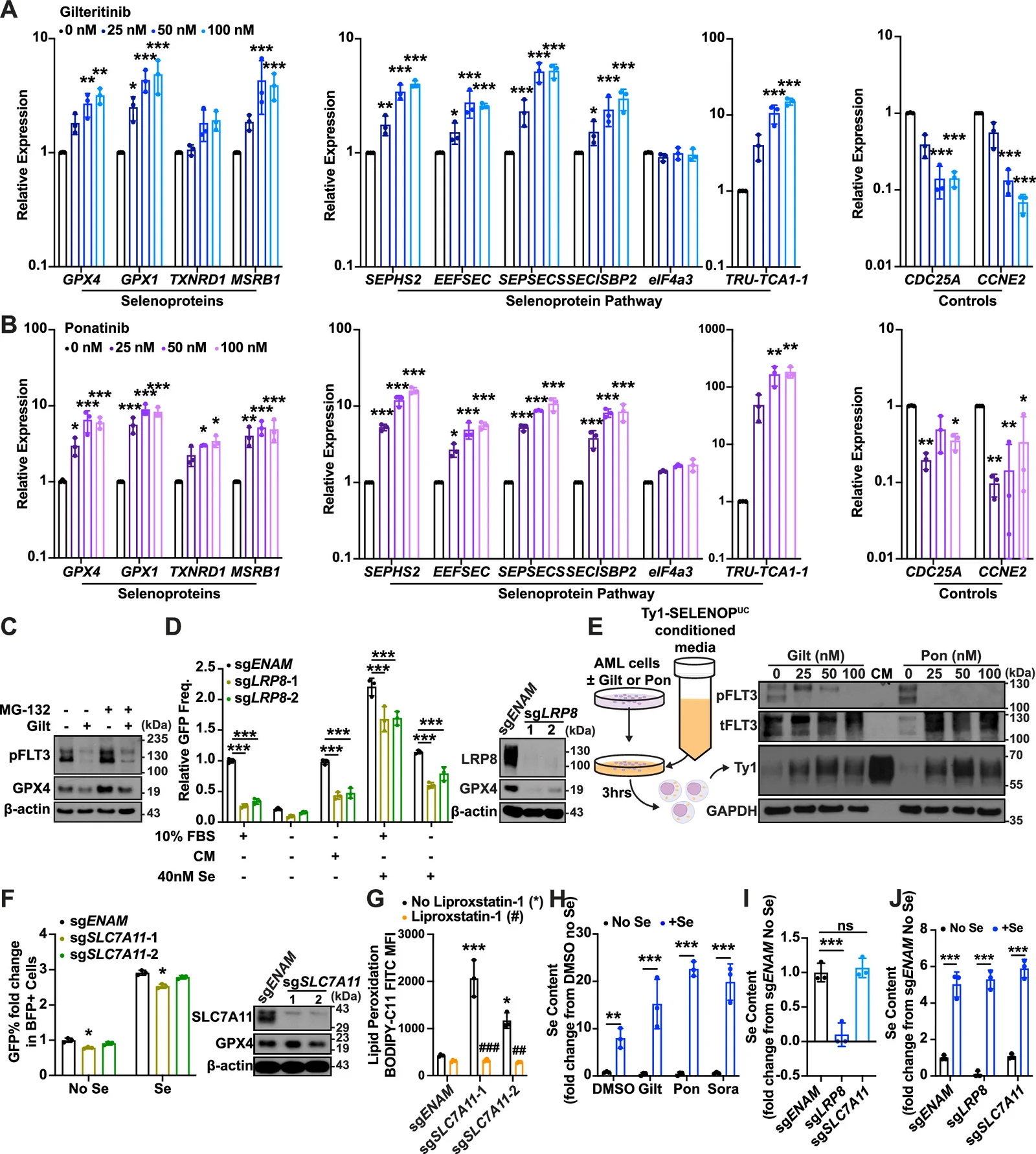
TKIs do not affect the transcription of selenoprotein biosynthesis gene or affect selenium uptake. **a**,**b**, Expression levels of genes in the selenoprotein biosynthesis pathway (*SEPHS2*, *EEFSEC*, *SEPSECS*, *SECISBP2*, *TRU-TCA1-1* and *EIF4A3*) and of selenoproteins (*GPX4*, *GPX1*, *TXNRD1* and *MSRB1*) when MOLM-13 cells were treated with varying concentrations of gilteritinib (**a**) or ponatinib (**b**) for 24 h. *CDC25A* and *CCNE2*, known downstream targets of FLT3 signalling, are included as controls. *P < 0.05, **P < 0.01 and ***P < 0.001 by two-way ANOVA with Dunnett’s post-hoc test for multiple comparisons, except for the use of one-way ANOVA for *TRU-TCA1-1*. *P* values of comparisons made between drug doses and the respective controls (0 nM) are (**a**) *GPX4*: 25 nM (*P* = 0.27), 50 nM (*P* = 0.008), 100 nM (*P* = 0.001); *GPX1*: 25 nM (*P* = 0.02), 50 nM and 100 nM (*P* < 0.001); *TXNRD1*: 25 nM (*P* > 0.99), 50 nM (*P* = 0.27), 100 nM (*P* = 0.19); *MSRB1*: 25 nM (*P* = 0.23), 50 nM and 100 nM (*P* < 0.001); *SEPHS2*: 25 nM (*P* = 0.001), 50 nM and 100 nM (*P* < 0.001); *EEFSEC*: 25 nM (*P* = 0.03), 50 nM and 100 nM (*P* < 0.001); *SEPSECS*: all comparisons *P* < 0.001; *SECISBP2*: 25 nM (*P* = 0.02), 50 nM and 100 nM (*P* < 0.001); *eIF4a3*: 25 nM (*P* = 0.93), 50 nM and 100 nM (*P* > 0.99); *TRU-TCA1-1*: 25 nM (*P* = 0.18), 50 nM and 100 nM (*P* < 0.001); *CDC25A*: all comparisons *P* < 0.001; *CCNE2*: all comparisons *P* < 0.001; (**b**) *GPX4*: 25 nM (*P* = 0.04), 50 nM and 100 nM (*P* < 0.001); *GPX1*: all comparisons *P* < 0.001; *TXNRD1*: 25 nM (*P* = 0.26), 50 nM (*P* = 0.04), 100 nM (*P* = 001); *MSRB1*: 25 nM (*P* = 0.002), 50 nM and 100 nM (*P* < 0.001); *SEPHS2*: all comparisons *P* < 0.001; *EEFSEC*: 25 nM (*P* = 0.04), 50 nM and 100 nM (*P* < 0.001); *SEPSECS*: all comparisons *P* < 0.001; *SECISBP2*: all comparisons *P* < 0.001; *eIF4a3*: 25 nM (*P* = 0.87), 50 nM (*P* = 0.62), 100 nM (*P* = 0.57); *TRU-TCA1-1*: 25 nM (*P* = 0.37), 50 nM (*P* = 0.002), 100 nM (*P* = 0.001); *CDC25A*: 25 nM (*P* = 0.010), 50 nM (*P* = 0.07), 100 nM (*P* = 0.03); *CCNE2*: 25 nM (*P* = 0.006), 50 nM (*P* = 0.008), 100 nM (*P* = 0.02). Results were derived from biological replicates (*n* = 3). **c**, MOLM-13 cells were treated with gilteritinib (25 nM) or MG-132 (200 nM) for 24 h and assessed for phospho-FLT3 and GPX4 levels. **d**, The effect of *LRP8* deletion on SELENOP-mediated increase in SecGR. *LRP8* was deleted with lentiviral sgRNA transduction of SecGR cells. *LRP8* deletion reduced SecGR signal when cells were cultured in media containing 10% FBS or CM. ***P < 0.001 by two-way ANOVA with Tukey’s post-hoc test for multiple comparisons. *P* values of comparisons made between *LRP8* sgRNAs and control (sg*ENAM*) in each media conditions are 10% FBS media, 100% CM, 0% FBS media + 40 nM Se, empty media + 40 nM Se: all comparisons *P* < 0.001; empty media: *LRP8* sg1 (*P* = 0.25), sg2 (*P* = 0.72). Results were derived from biological replicates (*n* = 3). **e**, Schematic representation (left) and the results of the SELENOP1 uptake assay (right). Detection of intracellular Ty1-tagged SELENOP1^UC^ was increased after gilteritinib or ponatinib treatment. CM; conditioned media. **f**, The effects of deleting *SLC7A11* on SecGR signal. *P < 0.05 by two-way ANOVA with Dunnett’s test for multiple comparisons. *P* values of *SLC7A11* sgRNAs and control (sg*ENAM*) are *SLC7A11* sg1 (*P* = 0.03), sg2 (*P* = 0.24). *P* values for genes deleted in the presence of selenium are *SLC7A11* sg1 (*P* = 0.01), sg2 (*P* = 0.17). Results were derived from biological replicates (*n* = 3). **g**, Lipid peroxidation levels in *SLC7A11*-deleted cells measured by BODIPY-C11. *P < 0.05, ** and ^##^*P* < 0.01; and *** and ^###^*P* < 0.001 by two-way ANOVA with Tukey’s post-hoc for multiple comparisons. *P* values for control (sg*ENAM*) versus sg*SLC7A11* are <0.001 (sg1), 0.01 (sg2); gene deletion in the presence of Lip-1 are 0.44 (sg*ENAM*), <0.001 (sg*SLC7A11*-1), 0.003 (sg*SLC7A11*-2). Results were derived from biological replicates (*n* = 3). **h**, MOLM-13 cells treated with DMSO (control), gilteritinib (Gilt), ponatinib (Pon) or sorafenib (Sora), with or without sodium selenite (Se) supplementation were analysed with ICP-MS for cellular selenium content. 5 × 10^6^ cells were collected per measurement. *P < 0.05 and ***P < 0.001 by two-way ANOVA with Tukey’s post-hoc test for multiple comparisons. *P* values for No selenite (Se) versus with selenite treatment are 0.003 (DMSO), <0.001 (gilteritinib, ponatinib and sorafenib). Results were derived from biological replicates (*n* = 3). **i**,**j**, ICP-MS analyses of cellular selenium content for control (sg*ENAM*), *LRP8*-deleted or *SLC7A11*-deleted MOLM-13 cells (**i**), with or without sodium selenite presence (**j**). ***P < 0.001 with one-way ANOVA with Dunnett’s post-hoc test for multiple comparisons (**i**) or two-way ANOVA with Tukey’s post-hoc test for multiple comparisons (**j**). *P* values for comparisons in (**i**) are <0.001 (sg*ENAM* vs sg*LRP8*) and 0.82 (sg*ENAM* vs sg*SLC7A11*); in (**j**) all comparisons have *P* < 0.001. Results were derived from biological replicates (*n* = 3). Immunoblot images are representative images of samples ran on the same gel as controls. Each of these experiments was performed multiple times. All data are represented as the mean ± s.d. Source data

The reduction of SecGR driven by a heterologous promoter suggests that FLT3 inhibition reduces selenoprotein biosynthesis post-transcriptionally. Gilteritinib or ponatinib did not reduce (but rather increased) the messenger RNA levels of several selenoproteins such as *GPX4*, *GPX1*, *SEPHS2*, *TXNRD1* and *MSRB1* (Extended Data Fig. 4a,b). Furthermore, these treatments did not reduce, but rather increased, the transcript level of genes involved in selenoprotein biosynthesis, that is, *SEPHS2*, *EEFSEC*, *SEPSECS*, *SECISBP2* and *TRU-TCA1-1* (encoding tRNA^[Ser]Sec^; Extended Data Fig. 4a,b). Gilteritinib reduced GPX4 even in the presence of MG-132 to inhibit the proteasome, which suggests that gilteritinib reduces GPX4 independently of proteasomal degradation (Extended Data Fig. 4c).

A recent study demonstrated that the inhibition of selenium uptake via *LRP8* deletion causes ribosome stalling at the *GPX4* UGA codon that is recoded to Sec in a selenium-replete condition^18^. In agreement with this, we observed reduced SecGR signal and GPX4 protein levels following *LRP8* deletion (Extended Data Fig. 4d). LRP8 binds and internalizes SELENOP, a Sec-rich protein that serves as a selenium carrier. We thus tested whether FLT3 inhibition suppress SELENOP uptake. We produced recombinant Ty1-tagged SELENOP that carries ten Sec-to-Cys substitutions (SELENOP^UC^) and undergoes receptor-dependent endocytosis^40^. MOLM-13 cells were subjected to treatment with or without gilteritinib and Ty1-SELENOP^UC^ uptake was assessed by immunoblotting. We found that Ty1-SELENOP^UC^ uptake by MOLM-13 cells was not reduced but instead enhanced by treatment with gilteritinib or ponatinib, indicating that FLT3 activity is not required for SELENOP uptake (Extended Data Fig. 4e). On the contrary, disruption of system x_c_^−^ by deleting *SLC7A11* induced ferroptosis but only modestly reduced SecGR signals without reducing GPX4 protein (Extended Data Fig. 4f,g). Assessment of elemental Se in MOLM-13 cells by inductively coupled plasma mass spectrometry (ICP-MS) revealed that FLT3 inhibitors failed to reduce cellular Se augmented by selenite treatment (Extended Data Fig. 4h). Deletion of *LRP8* but not *SLC7A11* reduced cellular selenium (Extended Data Fig. 4i,j). These results suggest that FLT3 inhibitors reduce GPX4 protein largely independent of selenium uptake.

### FLT3 inhibition reduces selenocysteine recoding

**Fig. 4:**
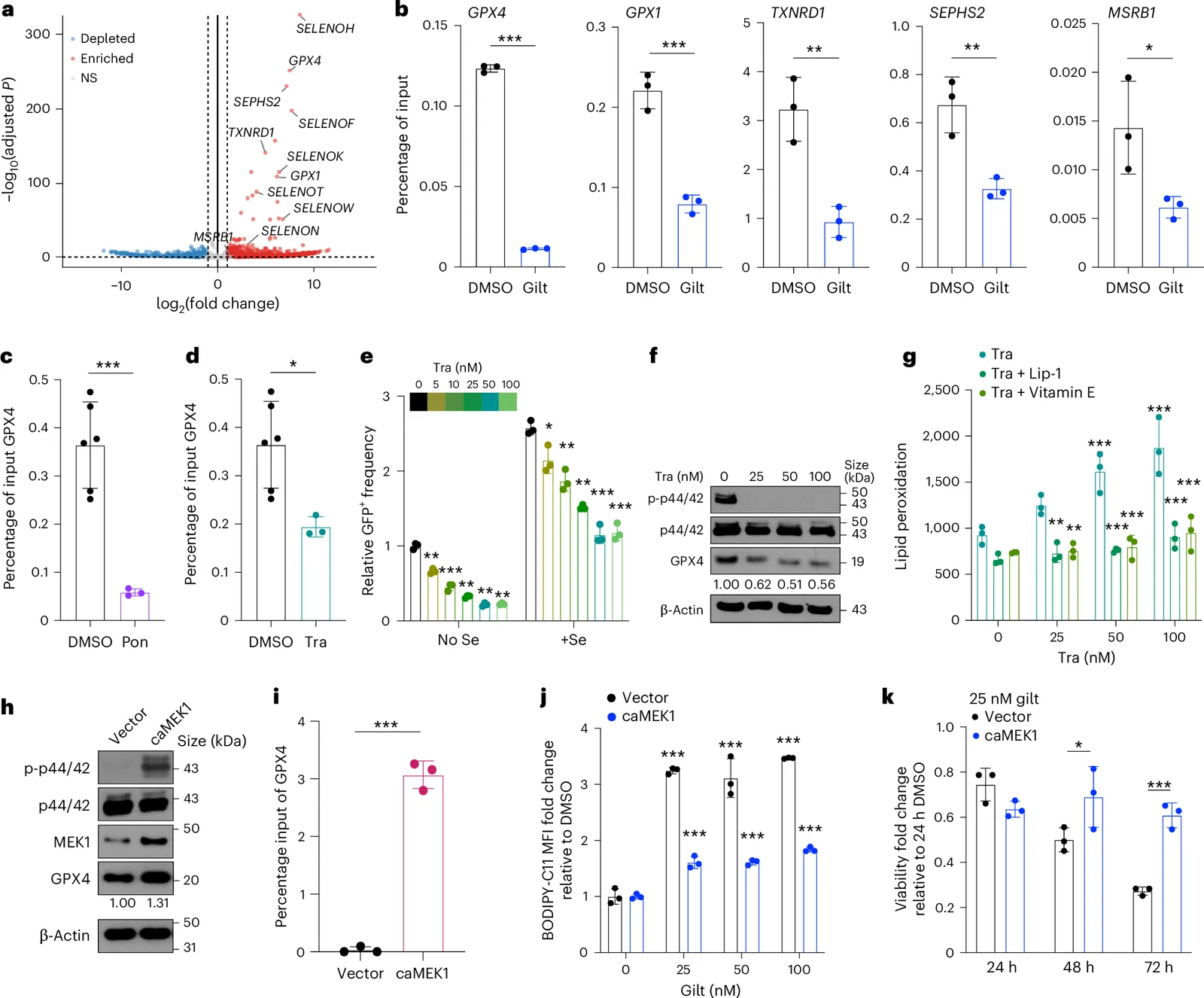
FLT3 inhibition reduces SECISBP2 binding to SECIS. **a**, Volcano plot of genes identified in RIP-seq of SECISBP2 in MOLM-13 cells. DESeq2 was used to identify enrichment trends across samples. Vertical dashed lines indicate log_2_(fold change) of −1 and 1; horizontal dashed line indicates −log_10_(0.05). Results were derived from *n* = 2 independent biological replicates; NS, not significant. **b**, Levels of the indicated selenoprotein mRNA, detected by SECISBP2 RNA-IP, in MOLM-13 cells following treatment with 50 nM gilteritinib. For comparisons between control (DMSO) and drug treatment, *P* <0.001 (*GPX4* and *GPX1*), *P* = 0.005 (*TXNRD1*), *P* = 0.008 (*SEPHS2*) and *P* = 0.04 (*MSRB1*). **c**,**d**, Levels of SECISBP2-bound *GPX4* mRNA in MOLM-13 cells following treatment with 50 nM ponatinib (**c**) or trametinib (**d**). Results were derived from *n* = 6 (DMSO treatment) and 3 (drug treatment) biological replicates. **d**, *P* = 0.02. **b**–**d**, Statistical comparisons were performed using an unpaired two-tailed Student’s *t*-test. **e**, Effects of trametinib, with or without selenium treatment, on SecGR signal. Cells in the 0 nM group were treated with DMSO. Values were normalized to the ‘No Se, 0 nM (DMSO treated)’ group. Control versus trametinib, *P* = 0.008 (5 nM), 0.001 (25 and 50 nM), 0.002 (100 nM) and <0.001 (10 nM); control versus trametinib in the presence of selenium, *P* = 0.04 (5 nM), 0.008 (10 nM), 0.009 (25 nM) and <0.001 (50 and 100 nM); two-way ANOVA with Dunnett’s post-hoc test for multiple comparisons. **f**, Effects of trametinib on phospho-ERK1/2 (p-p44/42) and GPX4 protein levels in MOLM-13 cells. **g**, Effects of trametinib on lipid peroxidation, indicated by BODIPY-C11 signal, which was suppressed by liproxstatin-1 and vitamin E. Trametinib versus control, *P* = 0.11 (25 nM) and <0.001 (50 nM and 100 nM); trametinib versus trametinib plus liproxstatin-1, *P* = 0.13 (0 nM), 0.004 (25 nM) and <0.001 (50 and 100 nM); trametinib versus trametinib plus vitamin E, *P* = 0.32 (0 nM), 0.006 (25 nM) and <0.001 (50 and 100 nM); two-way ANOVA with Tukey’s post-hoc test for multiple comparisons. **h**, Expression of caMEK1 increases GPX4 protein along with phospho-ERK1/2 levels in MOLM-13 cells. **f**,**h**, Representative immunoblot images with samples and controls run on the same gel are shown. Each of these experiments was performed multiple times. The relative GPX4 levels are shown below the immunoblots. **i**, Levels of *GPX4* mRNA bound to SECISBP2 in MOLM-13 cells with or without the expression of caMEK1. Statistical comparisons were performed using an unpaired two-tailed Student’s *t*-test. **j**,**k**, Expression of caMEK1 reduces gilteritinib-induced lipid peroxidation, as determined by BODIPY-C11 staining (**j**), and renders cells partially resistant to gilteritinib (**k**). Statistical comparisons were performed using a two-way ANOVA with Dunnett’s post-hoc test for multiple comparisons (**j**) and two-way ANOVA with Bonferroni post-hoc test for multiple comparisons (**k**). **j**, *P* < 0.001 for all comparisons (control (0 nM) versus gilteritinib of vector-expressing and vector versus caMEK1-expressing cells). **k**, Vector versus caMEK1-expressing cells, *P* = 0.26 (24 h), *P* = 0.02 (48 h), *P* < 0.001 (72 h). **b**,**e**,**g**,**h**–**k**, Results were derived from *n* = 3 biological replicates. All data are represented as the mean ± s.d. *P < 0.05, **P < 0.01, ***P < 0.001. Gilt, gilteritinib; lip-1, liproxstatin-1; pon, ponatinib; tra, trametinib. Source data

**Extended Data Fig. 5:**
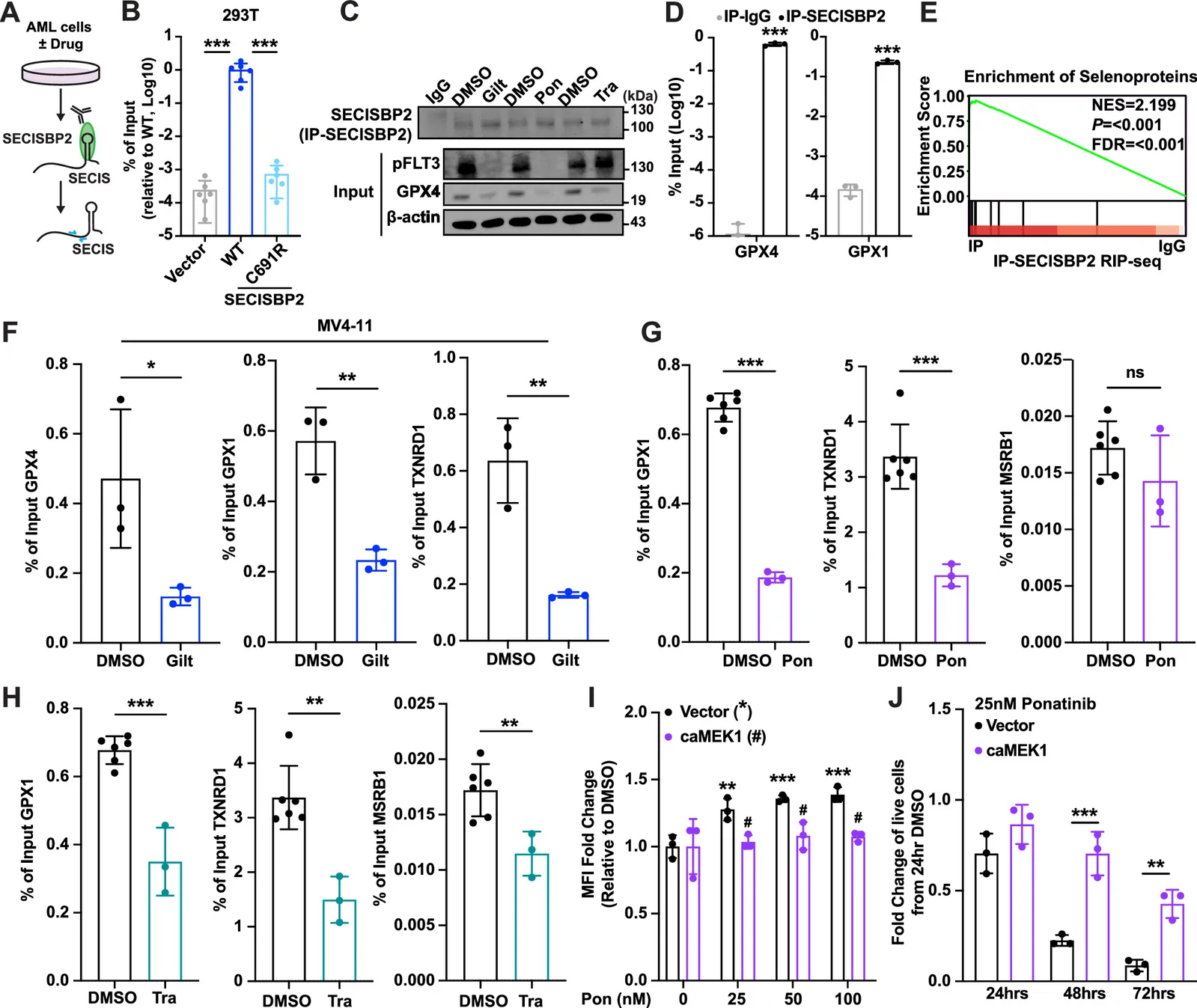
TKIs reduce SECISBP2 binding to SECIS of selenoprotein mRNA. **a**, Schematic representation of SECISBP2 RNA-IP assays to detect selenoprotein mRNAs bound to SECISBP2**. b**, RNA-IP assay to detect the binding of SECISBP2 to the SECIS element of *GPX4* mRNA in 293T cells transfected with vector, FLAG-SECISBP2 or FLAG-SECISBP2-C691R. ***P < 0.001 by one-way ANOVA with Dunnett’s post-hoc test for multiple comparisons. *P* values of all comparisons are <0.001. Results were derived from biological replicates (*n* = 6). **c**. Immunoprecipitation of SECISBP2 in MOLM-13 cells after treatment with gilteritinib (Gilt), ponatinib (Pon), or trametinib (Tra). Immunoblotted for SECISBP2 in the immunoprecipitated samples, pFLT3 and GPX4 in the input samples. **d**, RNA-IP assay to detect the binding of SECIS elements of *GPX4* and *GPX1* mRNA in MOLM-13 cells bound to SECISBP2. IP performed with IgG or anti-SECISBP2 antibodies. ***P < 0.001 by unpaired, two-tailed Student’s t-test. *P* values for all comparisons are <0.001. Results were derived from biological replicates (*n* = 3). **e**, GSEA against a custom list of 25 selenoprotein genes showing that RNA-IP sequencing (RIP-seq) of SECISBP2 is highly enriched for selenoprotein genes compared with IgG control. Normalized enrichment score (NES) calculated using the GSEA package in R. *P* values for enrichment are calculated from permutation testing and adjusted for multiple-testing correction using Benjamini–Hochberg FDR (p.adjust). **f**, RNA-IP assays in MV4-11 cells treated with gilteritinib to detect SECISBP2 binding to *GPX4, GPX1, and TXNRD1* SECIS. *P < 0.05 by unpaired, two-tailed Student’s t-test. *P* values are 0.04 (*GPX4*), 0.004 (*GPX1*), 0.005 (*TXNRD1*). Results were derived from biological replicates (*n* = 3). **g**,**h**, RNA-IP assays in MOLM-13 cells treated with ponatinib (Pon, **g**) or trametinib (Tra, **h**) to detect SECISBP2 binding to *GPX1, TXNRD1* and *MSRB1* SECIS. **P < 0.01 and ***P < 0.001 by unpaired, two-tailed Student’s t-test. *P* values for ponatinib (**g**) treated samples are <0.001 (*GPX1* and *TXNRD)*, 0.20 (*MSRB1*); (**h**) trametinib treated samples are <0.001 (*GPX1*), 0.002 (*TXNRD1*), 0.009 (*MSRB1*). Results were derived from biological replicates, DMSO (control, *n* = 6) and drug treatment (*n* = 3). **i**, Lipid peroxidation levels assessed by BODIPY-C11 staining of MOLM-13 infected with a control (vector) or caMEK1-expressing vector treated with ponatinib. Values are normalized to control (0 nM, DMSO treatment) treatment of vector- or caMEK1-expressing cells. *P < 0.05, **P < 0.01; ***P < 0.001 and ^###^*P* < 0.01 by two-way ANOVA with Dunnett’s post-hoc test for multiple comparisons. *P* values for drug effect on vector are 0.008 (25 nM) and <0.001 (50 and 100 nM); and comparison of vector and caMEK1 are >0.99 (0 nM), 0.04 (25 nM), 0.02 (50 nM), 0.01 (100 nM). Results were derived from biological replicates (*n* = 3). **j**, Cell viability of MOLM-13 with or without caMEK1 expression treated with ponatinib. Values are normalized to those of control (0 nM, DMSO treatment) treatment of vector- or caMEK1-expressing cells. Control data is available in raw data file. **P < 0.01; and ***P < 0.001 by two-way ANOVA with Bonferroni post-hoc test for multiple comparisons. *P* values for comparison between vector and caMEK1-expressing cells are 0.13 (24 h), <0.001 (48 h), 0.001 (72 h). Results were derived from biological replicates (*n* = 3). Immunoblot images are representative images of samples ran on the same gel as controls. Each of these experiments was performed multiple times. All data are represented as the mean ± sd. Source data

Binding of SECISBP2 to SECIS to recode UGA to Sec is the rate-limiting step in selenoprotein biosynthesis^15^. We thus examined whether TKIs affect SECISBP2 binding to SECIS by RNA-immunoprecipitation (RNA-IP), in which we immunoprecipitated SECISBP2 and quantified bound selenoprotein mRNA by quantitative PCR, at sites proximal to the SECIS element (Extended Data Fig. 5a). First, we confirmed that SECISBP2 binding to the SECIS element is dependent on its RNA-binding domain, as a C691R mutation in this domain completely abolished its binding to *GPX4* mRNA (Extended Data Fig. 5b). RNA-IP sequencing (RIP-seq) of SECISBP2 in MOLM-13 cells further validated that SECISBP2 preferentially binds to selenoprotein mRNA in AML cells. Notably, GPX4 was among the most significantly enriched mRNA bound to SECISBP2 (Fig. 4a and Extended Data Fig. 5c–e). We then performed SECISBP2 RNA-IP with MOLM-13 and MV4-11 cells treated with gilteritinib. SECISBP2 binding to *GPX4* mRNA, along with other selenoprotein mRNA such as *GPX1*, *TXNRD1*, *SEPHS2* and *MSRB1*, was reduced by gilteritinib treatment (Fig. 4b and Extended Data Fig. 5f). These results suggest that FLT3 inhibition reduces the translation of a subset of selenoproteins, including GPX4, by interfering with SECISBP2 binding to the SECIS element.

To explore whether the other serenibs identified in our drug screening also suppress SECISBP2 binding to SECIS, we treated MOLM-13 cells with ponatinib and trametinib, a MEK inhibitor identified as a serenib (Fig. 1e), and performed SECISBP2 RNA-IP. Similar to gilteritinib, ponatinib and trametinib reduced SECISBP2 binding to *GPX4* and other selenoprotein mRNA, corroborating their effects on GPX4 protein (Fig. 4c,d and Extended Data Fig. 5g,h).

The identification of the MEK inhibitor trametinib as a serenib that reduces GPX4 protein levels—similar to FLT3 inhibitors—suggested that MEK may act downstream of FLT3 to promote Sec recoding, given that FLT3 is known to activate the MEK–MAPK pathway. Consistent with the screening results, trametinib reduced SecGR signal (Fig. 4e) and GPX4 protein, and increased lipid peroxidation (Fig. 4f,g). To determine the role of active MEK pathway in Sec recoding, we expressed constitutively active MEK1 (caMEK1) carrying S218D/S222D mutations (Fig. 4h). Constitutively active MEK1 promoted SECISBP2 binding to *GPX4* mRNA, increased GPX4 protein, reduced lipid peroxidation triggered by gilteritinib or ponatinib treatment and rendered cells less sensitive to these FLT3 inhibitors (Fig. 4i–k and Extended Data Fig. 5i,j). Collectively, these results suggest that the MEK pathway is among the pathways downstream of FLT3 that promote SECISBP2 binding the SECIS elements of genes encoding several selenoproteins, including *GPX4*, to augment Sec recoding and ferroptosis evasion. However, our results only provide support for a functional link between MEK and SECISBP2, and the mechanism by which the MEK pathway regulates SECISBP2 binding to SECIS remains unclear.

### GPX4 is a vulnerability of *FLT3*-ITD AML

**Fig. 5:**
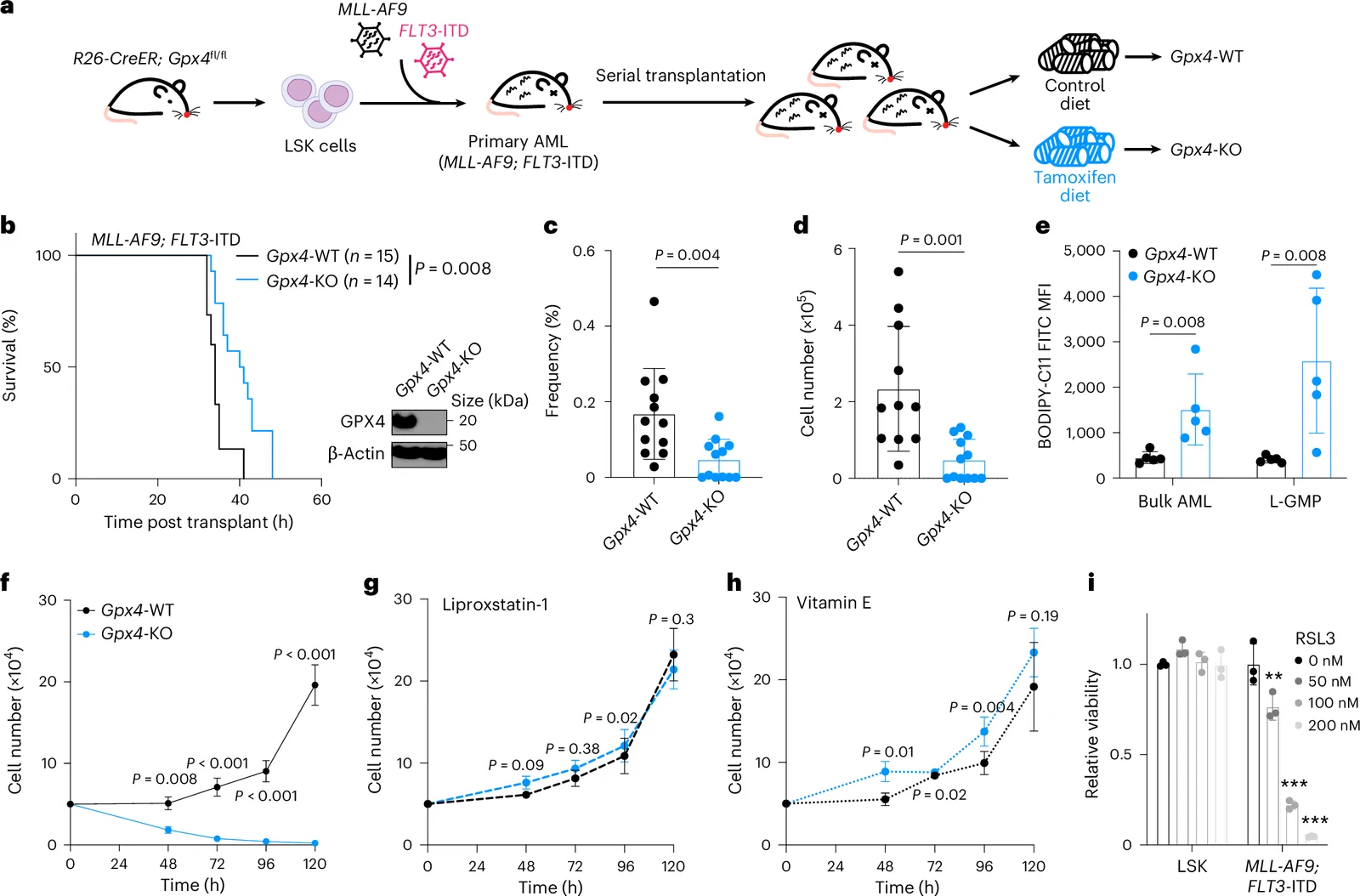
*FLT3*-ITD AML depends on GPX4. **a**, Experimental strategy to delete *Gpx4* from established *MLL-AF9;* *FLT3-*ITD AML in mice. **b**, Survival curve of mice in the *Rosa26–CreER;* *Gpx4*^fl/fl^ background transplanted with *MLL-AF9;* *FLT3-*ITD AML and fed chow with or without tamoxifen (left). Representative immunoblot of the GPX4 protein levels in AML cells isolated from recipient mice (*Gpx4-*WT, *n* = 15; *Gpx4-*KO, *n* = 14; right). Representative immunoblot images with samples and controls run on the same gel are shown. Each of these experiments was performed multiple times. A log-rank test was used for comparison of survival difference. **c**,**d**, Frequency (**c**) and total number (**d**) of L-GMPs in the bone marrow of AML recipient mice treated as in **b**. Statistical comparison performed using an unpaired two-tailed Student’s *t*-test (**c**, *n* = 12 biological replicates for both groups; **d**, *n* = 10 *Gpx4-*WT and 12 *Gpx4*-KO biological replicates). **e**, Levels of lipid peroxidation, as measured by BODIPY-C11, in bulk AML and L-GMPs of mice with *Gpx4*-WT and -KO *MLL-AF9;* *FLT3-*ITD AML as in **b**. Statistical comparisons were performed using a two-way ANOVA with Bonferroni’s post-hoc test for multiple comparisons. Results were derived from *n* = 5 biological replicates. **f**–**h**, *Gpx4*-WT and -KO *MLL-AF9;* *FLT3-*ITD AML cells isolated from mice treated as in **b** were cultured without (**f**) or with liproxstatin-1 (**g**) and vitamin E (**h**). Statistical comparisons were performed using a two-way ANOVA with Tukey’s post-hoc test for multiple comparisons. Results were derived from *n* = 3 biological replicates. **i**, Effects of RSL-3 on the viability of LSK and *MLL-AF9;* *FLT3-*ITD AML cells. Drug treatment versus control (DMSO, 0 nM): LSK cells, *P* = 0.19 (50 nM), *P* = 0.98 (100 nM) and *P* > 0.99 (200 nM); *MLL-AF9;* *FLT3-*ITD AML cells, *P* = 0.008 (50 nM) and ***P < 0.001 (100 and 200 nM); one-way ANOVA with Dunnet’s post-hoc test for multiple comparisons. Results were derived from *n* = 3 biological replicates. All data are represented as the mean ± s.d. Source data

**Extended Data Fig. 6:**
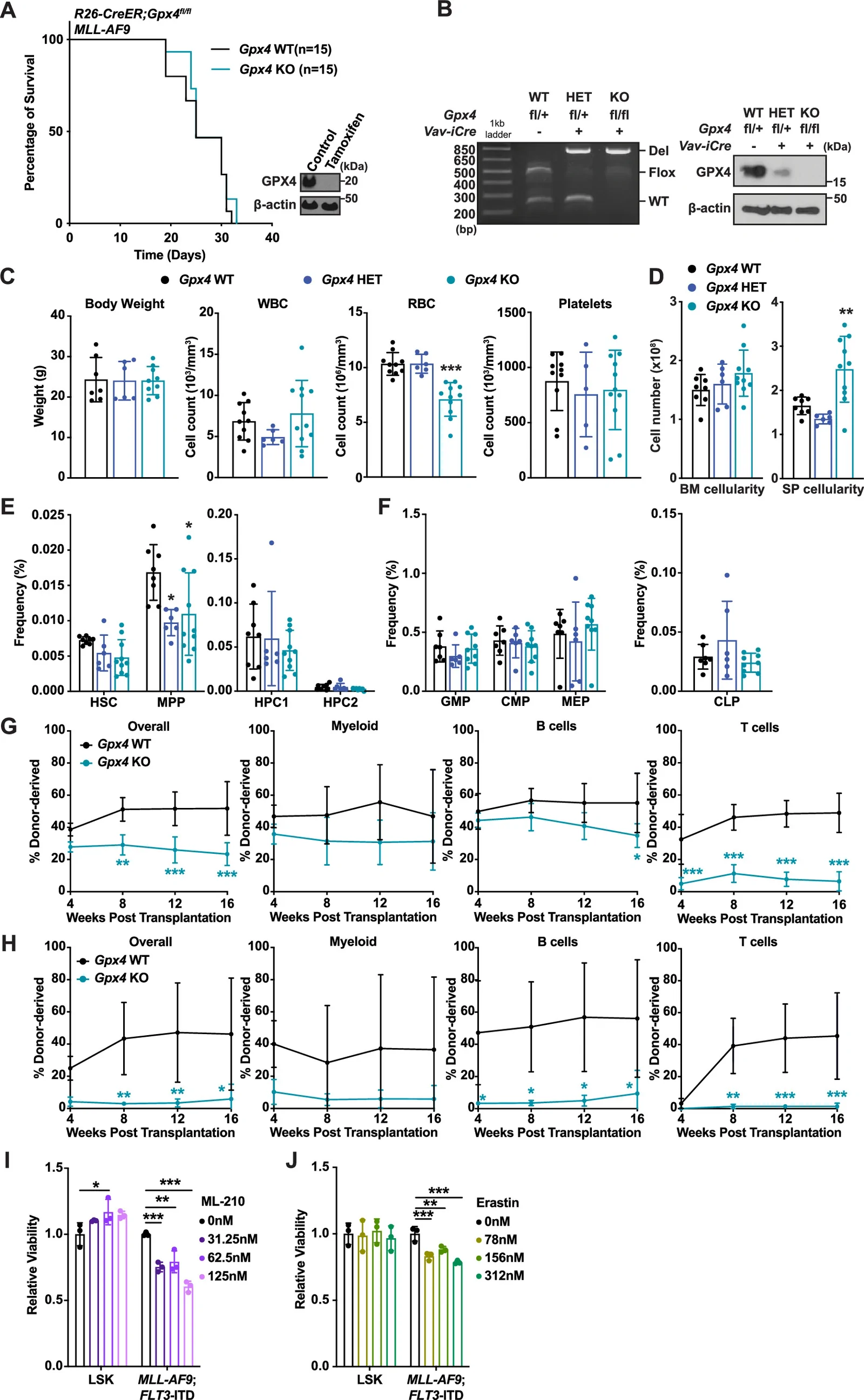
*Gpx4* is dispensable for haematopoiesis. **a**, Survival curves from a secondary transplantation assay with *Gpx4* WT or KO *MLL-AF9*-induced AML. *MLL-AF9*-induced AML generated in the *Rosa26*–*CreER*; *Gpx4*^fl/fl^ were fed with control or tamoxifen diet. AML cells isolated from these primary recipient mice were then used for the secondary transplantation assay, as shown in the schematic of Fig. 5a. GPX4 protein levels in AML cells isolated from moribund mice are shown in right. Statistical analysis of difference between survival curves were determined by log-rank test, *P* = 0.4552. Results were derived from biological replicates, *n* = 15 per group. **b**, Results from PCR genotyping gel (left) and immunoblotting (right) for GPX4 protein with mice carrying the indicated genotypes. **c**. Comparison of *Vav1-iCre; Gpx4*^*+/+*^ (*Gpx4* WT), *Vav1-iCre*; *Gpx4*^fl/+^ (*Gpx4* HET) and *Vav1-iCre*; *Gpx4*^fl/fl^ (*Gpx4* KO) mice for body weight, white blood cell (WBC) counts, red blood cell counts (RBC), and platelets. ***P < 0.001 by one-way ANOVA with Dunnett’s test for multiple comparisons. *P* values for comparisons made against *Gpx4* WT for body weight are >0.99 (*Gpx4* HET and KO); WBC: 0.37 (HET), 0.70 (KO); RBC: 0.99 (HET), <0.001 (KO); platelets: 0.74 (HET), 0.82 (KO). Results were derived from biological replicates for body weight: *Gpx4* WT (*n* = 7), HET (*n* = 6), KO (*n* = 9); WBC: *Gpx4* WT (*n* = 10), HET (*n* = 6), KO (*n* = 11); RBC: *Gpx4* WT (*n* = 10), HET (*n* = 6), KO (*n* = 11); Platelets: *Gpx4* WT (*n* = 10), HET (*n* = 5), KO (*n* = 11). **d**, Cellularity of the bone marrow and spleens of *Gpx4* WT, HET and KO mice. **P < 0.01 by one-way ANOVA with Dunnett’s test for multiple comparisons. *P* values for comparisons made against *Gpx4* WT for bone marrow are 0.81 (HET), 0.16 (KO); spleen: 0.46 (HET), 0.004 (KO). Results were derived from biological replicates, *Gpx4* WT (*n* = 8), HET (*n* = 6), KO (*n* = 10). **e**,**f**, Frequencies of haematopoietic stem and progenitor cell populations in the bone marrow within the lineage^−^Sca-1^+^c-kit^+^ (LSK) fraction (**e**) and myeloid- or lymphoid-restricted progenitor cells (**f**). Statistical analysis one-way ANOVA with Tukey’s post-hoc test for multiple comparisons. *P* values for comparisons made against *Gpx4* WT in (**e**) HSC are 0.27 (HET), KO 0.06 (KO); MPP: 0.02 (HET), 0.03 (KO); HPC1: >0.99 (HET), 0.65 (KO); HPC2: >0.99 (HET), 0.29 (KO). (**F**) GMP: 0.45 (HET), 0.95 (KO); CMP: 0.95 (HET), 0.72 (KO); MEP: 0.89 (HET), 0.80 (KO); CLP: 0.38 (HET), 0.85 (KO). Results were derived from biological replicates, in (**e**), *Gpx4* WT (*n* = 8), HET (*n* = 6), KO (*n* = 10); in (**f**) *Gpx4* WT (*n* = 6), HET (*n* = 6), KO (*n* = 9). **g**, Competitive bone marrow transplantation assays with *Gpx4* WT and KO mice as donors. Donor-derived chimerism in overall peripheral blood mononucleated cells, myeloid, B and T cell populations are shown. *P < 0.05, **P < 0.01; and ***P < 0.001 by mixed effects model of two-way ANOVA with Šídák’s post-hoc test for multiple comparisons. *P* values for comparisons made between *Gpx4* WT and KO for overall chimerism are 0.18 (4 weeks), 0.003 (8 weeks), <0.001 (12 weeks and 16 weeks); Myeloid: 0.76 (4 weeks), 0.63 (8 weeks), 0.19 (12 weeks), 0.47 (16 weeks); B cells: 0.84 (4 weeks), 0.42 (8 weeks), 0.11 (12 weeks), 0.01 (16 weeks); T cells: all comparisons *P* < 0.001. Results were derived from biological replicates, six recipients per group. **h**, Secondary bone marrow transplantation using 5×10^5^ whole bone marrow cells from recipient mice as in **g**. Donor-derived chimerism in overall peripheral blood mononucleated cells, myeloid, B and T cell populations are shown. *P < 0.05, **P < 0.01 and ***P < 0.001 by mixed effects model of two-way ANOVA with Šídák’s post-hoc test for multiple comparisons. *P* values for comparisons made between *Gpx4* WT and KO for overall chimerism are 0.34 (4 weeks), 0.010 (8 weeks), 0.009 (12 weeks), 0.02 (16 weeks); Myeloid: 0.34 (4 weeks), 0.40 (8 weeks), 0.30 (12 weeks), 0.32 (16 weeks); B cells: 0.04 (4 weeks), 0.02 (8 weeks), 0.01 (12 weeks), 0.03 (16 weeks); T cells: >0.99 (4 weeks), 0.002 (8 weeks), <0.001 (12 weeks and 16 weeks). Results were derived from biological replicates, five recipients per group. **i**,**j**, Viability of murine LSK and *MLL-AF9*; *FLT3*-ITD AML cells treated with ML-210 (**i**) or erastin (**j**). *P < 0.05, **P < 0.01 and ***P < 0.001 by one-way ANOVA with Dunnet’s post-hoc test for multiple comparisons. *P* values for comparison of different doses with control (DMSO, 0 nM) in (**i**) are 0.21 (31.25 nM), 0.04 (2.5 nM), 0.07 (125 nM) for LSK cells; <0.001 (31.25 nM), 0.002 (62.5 nM), <0.001 (125 nM) for *MLL-AF9;* *FLT3-*ITD AML cells; in (**j**) are >0.99 (78 nM), 0.98 (156 nM), 0.95 (312 nM) for LSK cells; <0.001 (78 nM), 0.007 (156 nM), <0.001 (312 nM) for *MLL-AF9;* *FLT3-*ITD AML cells. Results were derived from biological replicates (*n* = 3). Immunoblot and gel images are representative images of samples ran on the same gel as controls. Each of these experiments was performed multiple times. All data are represented as the mean ± s.d. Source data

Previous studies have shown the importance of GPX4 in suppressing ferroptosis in several cancer cell line models. However, the role of GPX4 has not been established in animal models of cancer. We thus established murine models of AML by expressing *MLL-AF9* with or without *FLT3*-ITD in a background allowing for conditional deletion of *GPX4* via *Rosa26–CreER* (*Rosa26–CreER;* *Gpx4*^fl/fl^; hereafter referred to as *Gpx4*^iKO^). After generating *MLL-AF9-* and *MLL-AF9*; *FLT3*-ITD-induced AML cells in a *Gpx4*^iKO^ background, we serially transplanted these cells into recipient mice, followed by CreER activation via treatment with or without tamoxifen (referred to as *Gpx4-*KO and -WT mice, respectively; Fig. 5a). As shown earlier, recipient mice transplanted with *Gpx4*-WT *MLL-AF9*; *FLT3*-ITD-induced AML cells had shorter leukaemia-free survival than those transplanted with *MLL-AF9-*induced AML (Extended Data Fig. 1k). *Gpx4* deletion from established *MLL-AF9*; *FLT3*-ITD AML delayed leukaemogenesis (Fig. 5b) and reduced the leukemic granulocyte-monocyte progenitor cells (L-GMPs), the leukaemia-initiating cells in the *MLL-AF9*-induced AML model^41^ (Fig. 5c,d). Furthermore, *Gpx4* deletion increased lipid peroxidation in L-GMPs and bulk AML cells (Fig. 5e), and reduced the viability of *MLL-AF9*; *FLT3*-ITD AML cells ex vivo, which was significantly rescued by liproxstatin-1 or vitamin E (Fig. 5f–h). In contrast, *Gpx4* deletion from *MLL-AF9*-induced AML cells did not affect leukaemogenesis (Extended Data Fig. 6a). Consistent with the heightened dependency of *FLT3*-ITD AML to GPX4, *FLT3*-ITD increased the expression of GPX4 in *MLL-AF9*-induced AML (Fig. 1i). These results establish that *Gpx4* is a genetic dependency of *FLT3*-mutant AML.

Despite the requirement of *Gpx4* in AML, *Gpx4* was largely dispensable for normal haematopoiesis. Conditional deletion of *Gpx4* from the haematopoietic system of *Vav1-iCre;* *Gpx4*^fl/fl^ (*Gpx4*-KO) mice reduced the numbers of red blood cells but not white blood cells or platelets in the peripheral blood or the cellularity of their bone marrow and spleens compared with *Vav1-iCre;* *Gpx4*^+/+^ (*Gpx4*-WT) and *Vav1-iCre;* *Gpx4*^+/fl^ (*Gpx4*-HET) mice at 15–20 weeks of age (Extended Data Fig. 6b–d). *Gpx4* deletion only modestly affected the frequencies of haematopoietic stem (HSC) and progenitor cells (Extended Data Fig. 6e,f). To assess how *Gpx4* deletion affects HSC function, we performed competitive whole bone marrow transplantation assays with *Gpx4*-WT and -KO mice. *Gpx4*-KO whole bone marrow cells exhibited reduced B and T cell reconstitution but showed no defects in long-term myeloid reconstitution potential (Extended Data Fig. 6g). Following secondary transplantation, reduced overall reconstitution, mainly driven by reduced B and T cell reconstitution, was observed for *Gpx4*-KO cells (Extended Data Fig. 6h). Together, these results along with a previous study^42^ indicate that *Gpx4* is largely dispensable for HSC function but may regulate the long-term self-renewal capacity of HSCs.

To directly compare whether normal haematopoietic stem and progenitor cells and AML exhibit differences in GPX4 dependence, we compared the effects of GPX4 inhibition on untransformed murine haematopoietic progenitor cells (LSK cells) as well as LSK cells transformed with *MLL-AF9* and *FLT3*-ITD expression. Interestingly, murine AML cells transformed with *MLL-AF9* and *FLT3*-ITD were more sensitive to GPX4 inhibition by RSL-3 and ML-210 (ref. ^21^), and cystine uptake blockade by erastin^18^ compared with LSK cells, suggesting that GPX4 inhibition exploits a therapeutic window of AML (Fig. 5i and Extended Data Fig. 6i,j). These results further emphasise the therapeutic potential of strategies that reduce GPX4 in AML therapy without harming normal haematopoiesis.

### Vitamin E mitigates the efficacy of gilteritinib therapy

**Fig. 6:**
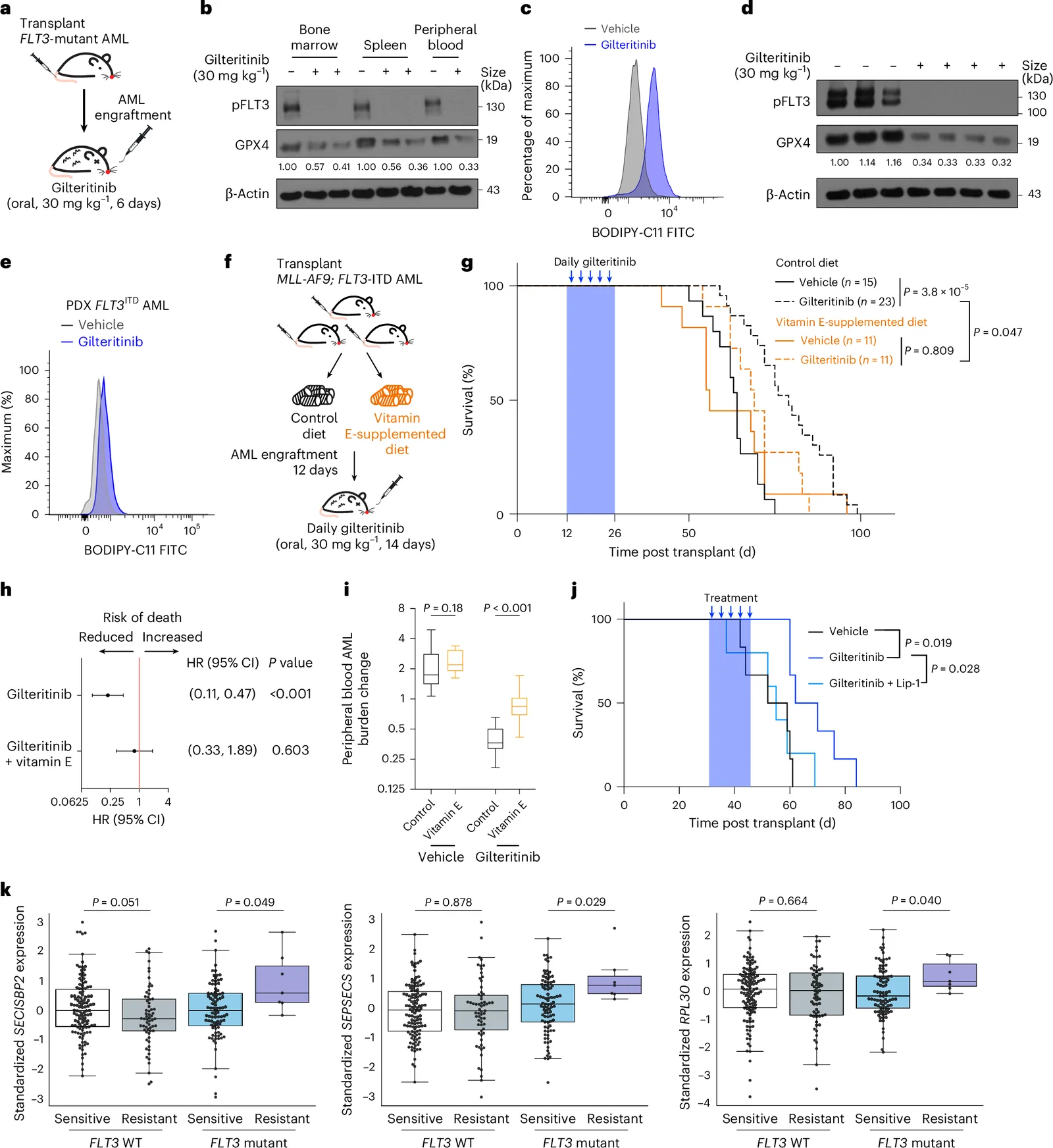
Dietary vitamin E attenuates the efficacy of gilteritinib therapy. **a**, Experimental strategy to test the effects of short-term gilteritinib treatment on *FLT3*-mutant AML. **b**, Changes in phospho-FLT3 and GPX4 protein expression in *MLL-AF9;* *FLT3*-ITD AML cells isolated from the bone marrow, spleen and peripheral blood of mice treated with vehicle (−) or gilteritinib (+). **c**, Representative histogram of BODIPY-C11 levels in *MLL-AF9;* *FLT3*-ITD AML cells of mice treated with vehicle or gilteritinib as in **b**. **d**, Changes in phospho-FLT3 and GPX4 protein expression in bone-marrow AML cells isolated from the *Vav1-NUP98-HOXD13;* *Flt3*^ITD/+^ knock-in AML model after vehicle or gilteritinib treatment. **b**,**d**, The relative GPX4 levels are shown below the immunoblots. Representative immunoblot images with samples and controls run on the same gel are shown. Each of these experiments was performed multiple times. **e**, Representative histogram of BODIPY-C11 levels in human AML cells isolated from *FLT3*-ITD + AML PDX mice treated with vehicle or gilteritinib. **f**, Experimental strategy to test the effects of vitamin E-supplemented diet on gilteritinib treatment in *MLL-AF9*; *FLT3*-ITD murine AML. **g**, Survival curves of *MLL-AF9*; *FLT3*-ITD AML recipient mice treated with or without gilteritinib and fed a vitamin E-supplemented or control diet as in **f**. The mice were pretreated with control or vitamin E-supplemented diet for two weeks before AML transplantation and then treated with gilteritinib or vehicle daily between days 12 to 26 post transplantation. Results were derived from *n* = 15 (control diet with vehicle), 23 (control diet with gilteritinib) and 11 (vitamin E diet with vehicle and vitamin E diet with gilteritinib) biological replicates. **h**, Forest plot showing the hazard ratios (HRs) of mice treated with gilteritinib with or without vitamin E-supplemented diet using data from **g**. The HR and 95% CI values were calculated using the Cox regression model. *P* values were derived from general linear hypothesis testing with an *F*-test. **i**, Fold change in AML cell frequencies in the peripheral blood of mice fed a control or vitamin E-supplemented diet and treated with gilteritinib for six days relative to pre-treatment. Comparisons within each treatment group (vehicle or gilteritinib) were performed using a two-tailed Student’s *t*-test. Results were derived from biological replicates; *n* = 12 mice fed control diet and treated with vehicle or gilteritinib; *n* = 9 and 10 mice fed vitamin E-supplemented diet and treated with vehicle or gilteritinib, respectively. **j**, Survival curves of *MLL-AF9*; *FLT3*-ITD AML recipient mice treated with vehicle, gilteritinib or gilteritinib in combination with liproxstatin-1 (Lip-1) on days 24–37 after transplantation. Results were derived from *n* = 6 (vehicle and gilteritinib treatment) and 5 (gilteritinib plus Lip-1 treatment) biological replicates. **g**,**j**, Statistical analyses of the survival curves were performed using a log-rank test. **k**, Levels of *SECISBP2* (left), *SEPSECS* (middle) and *RPL30* (right) expression in *FLT3* WT and mutant samples stratified according to whether or not they were resistant to gilteritinib. Each dot represents one sample. Gene expression values were normalized to the transcripts per million, log-transformed (log(*x* + 1)) and *z*-score standardized. Statistical significance was determined using a two-tailed Student’s *t*-test. *FLT3* WT sensitive, *n* = 118; *FLT3* WT resistant, *n* = 63; *FLT3* mutant sensitive, *n* = 89; *FLT3* mutant resistant, *n* = 7. **i**,**k**, The centre lines of the box plots show the median, the box borders represent the interquartile range and the whiskers extend to ±1.5× the interquartile range. All data are represented as the mean ± s.d. Source data

**Extended Data Fig. 7:**
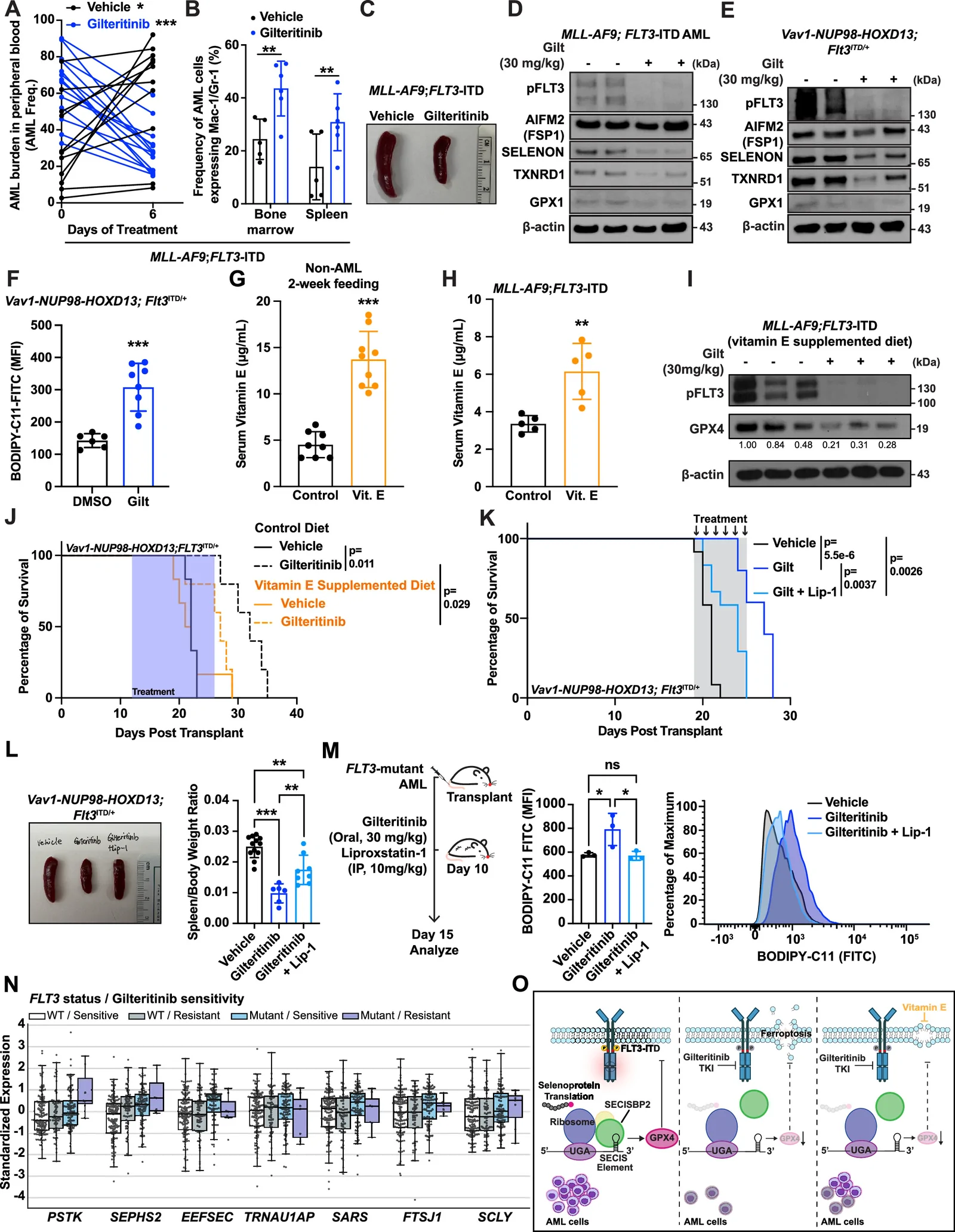
Gilteritinib reduces GPX4 protein in AML. **a**, Changes in peripheral blood AML frequencies of *MLL-AF9*; *FLT3*-ITD AML recipient mice before and after six days of vehicle or gilteritinib treatment. **P < 0.01 and ***P < 0.001 by unpaired, two-tailed Student’s *t*-test for comparison of before and after treatment. *P* values are 0.02 (vehicle) and <0.001 (gilteritinib). Results were derived from biological replicates, vehicle (*n* = 11) and gilteritinib treatment (*n* = 14). **b**, Frequencies of Mac-1^+^Gr-1^+^ AML cells in the bone marrow or spleen of *MLL-AF9*; *FLT3*-ITD AML recipient mice treated with vehicle or gilteritinib for six days. **P < 0.01 and *P < 0.05 by two-way ANOVA with Šídák’s post-hoc test for multiple comparisons. *P* values are 0.003 (bone marrow) and 0.006 (spleen). Results were derived from biological replicates, vehicle (*n* = 5) and gilteritinib treatment (*n* = 6). **c**, A representative image of the spleens of *MLL-AF9*; *FLT3*-ITD AML recipient mice after six days of vehicle or gilteritinib treatment in vivo. **d**,**e**, Immunoblotting of the indicated proteins in *MLL-AF9*; *FLT3-*ITD (**d**) or *Vav1-NUP98-HOXD13;* *FLT3*^ITD/+^ (**e**) mouse models after treatment with vehicle (Gilt −) or gilteritinib (Gilt +) for six days. **f**, Lipid peroxidation levels (BODIPY-C11) for ex vivo treatment of *Vav1-Nup98-HOXD13;* *Flt3*^*ITD/+*^ AML cells with gilteritinib. ***P < 0.001 by unpaired, two-tailed Student’s *t*-test. *P* value for comparison is <0.001. Results were derived from biological replicates, vehicle (*n* = 6), gilteritinib (*n* = 8). **g**,**h**, Serum vitamin E levels in wild-type (**g**) or AML recipient (**h**) mice after two weeks of feeding regular or vitamin E-supplemented diet (Vit. E). **P < 0.01; ***P < 0.001 by unpaired, two-tailed Student’s t-test. *P* value in (**g**) comparison is <0.001; and in (**h**) is 0.004. Results were derived from biological replicates, mice fed with control diet (*n* = 8) and mice fed with Vitamin E-supplemented diet (*n* = 9) in g; and five mice per group in **h**. **i**, Protein levels of phopsho-FLT3 and GPX4 in *MLL-AF9;* *FLT3*-ITD AML cells sorted from mice treated with vitamin E-supplemented diet and treated with or without gilteritinib for six days. **j**, Survival curves of *Vav-1-NUP98-HOXD13;* *FLT3*^ITD/+^ AML recipient mice on control- or vitamin E-supplemented diet treated with vehicle or gilteritinib (groups: control diet vehicle treatment (*n* = 6), control diet gilteritinib treatment (*n* = 5), vitamin E-supplemented diet vehicle treatment (*n* = 6), vitamin E-supplemented diet gilteritinib treatment (*n* = 5)). Blue shading indicates the period during which gilteritinib treatment was administered (day 12–26 post transplantation). Survival curve comparisons were done using the log-rank test. Results were derived from biological replicates. **k**, Survival curves of *Vav-1-NUP98-HOXD13;* *FLT3*^ITD/+^ AML recipients treated with vehicle, gilteritinib or combination of gilteritinib and liproxstatin-1 (Lip-1) (groups: vehicle treatment (*n* = 12), gilteritinib treatment (*n* = 11), gilteritinib and liproxstatin-1 treatment (*n* = 12)). Survival curve comparisons were done using the log-rank test. Results were derived from biological replicates. **l**, Representative images and weight quantification of spleens from *Vav-1-NUP98-HOXD13;* *FLT3*^ITD/+^ AML mice after receiving treatments described in **k**. **P < 0.01; ***P < 0.001 by two-way ANOVA with Tukey’s post-hoc test. *P* value for vehicle versus gilteritinib is <0.001, vehicle versus gilteritinib plus Lip-1 is 0.001, gilteritinib versus gilteritinib plus Lip-1 is 0.005. Results were derived from biological replicates, vehicle (*n* = 12), gilteritinib (*n* = 6), gilteritinib and liproxstatin-1 (*n* = 8). **m**, Experimental scheme to assess the effects of gilteritinib treatment on ferroptosis with the *Vav-1-NUP98-HOXD13;* *FLT3*^ITD/+^ AML model. AML cells isolated from AML recipient mice from each treatment group were analysed with BODIPY-C11 staining. *P < 0.05; **P < 0.01; ***P < 0.001 by two-way ANOVA or controlling for FDR. *P* value for vehicle versus gilteritinib is 0.03, vehicle versus gilteritinib plus Lip-1 is 0.90, gilteritinib versus gilteritinib plus Lip-1 is 0.02. Results were derived from biological replicates (*n* = 3). **n**, Gene expression from AML patients from BeatAML2 dataset. Box plots showing the expression levels of additional genes in the selenoprotein biosynthesis pathway in *FLT3* WT and mutant samples, as shown in Fig. 6k, divided into gilteritinib resistant and non-resistant samples. Statistical significance was determined using two-tailed Student’s *t*-test. *FLT3*^WT^-sensitive *n* = 118, *FLT3*^WT^-Resistant *n* = 63, *FLT3*^mutant^-Sensitive *n* = 89, *FLT3*^mutant^-Resistant *n* = 7. **o**, FLT3 signalling promotes selenoprotein biosynthesis by acting on the rate-limiting step in which SECISBP2 binds SECIS in 3′ UTR. Inhibition of FLT3 in *FLT3*-mutant cells lowers selenoprotein expression, especially GPX4, triggering ferroptosis. Antioxidants such as vitamin E suppresses ferroptosis, potentially compromising efficacy of FLT3 inhibitor therapies. Immunoblot images are representative images of samples ran on the same gel as controls. Each of these experiments was performed multiple times. All data are represented as the mean ± s.d. Source data

We then determined whether TKIs reduce GPX4 protein in AML in vivo. Recipient mice transplanted with *MLL-AF9;* *FLT3-*ITD AML cells were treated with gilteritinib for six days. GPX4 protein expression in AML cells isolated from the bone marrow, spleen and peripheral blood was then analysed (Fig. 6a). As expected, gilteritinib treatment reduced the frequency of AML cells in blood, promoted myeloid differentiation and ameliorated splenomegaly (Extended Data Fig. 7a–c). Gilteritinib treatment also reduced the levels of phospho-FLT3 and GPX4 protein in AML cells residing in the bone marrow, spleen and blood, and increased lipid peroxidation of AML cells in vivo (Fig. 6b,c and Extended Data Fig. 7d). Gilteritinib-induced GPX4 protein reduction and ferroptosis induction were also observed in AML from *Vav1-NUP98-HOXD13;* *Flt3*^ITD/+^ knock-in mice^43^ (Fig. 6d and Extended Data Fig. 7e,f). In addition, a *FLT3*-ITD + AML patient-derived xenograft (PDX) model^44^ treated with gilteritinib for six days showed increased lipid peroxidation in circulating human AML cells (Fig. 6e), indicating that gilteritinib induces ferroptosis in human AML.

The findings that the sensitivity to gilteritinib was partially suppressed by vitamin E raised the possibility that dietary vitamin E may modify the sensitivity of AML to gilteritinib in vivo. To test this, we fed mice transplanted with *MLL-AF9*; *FLT3*-ITD or *Vav1-NUP98-HOXD13;* *Flt3*^ITD/+^ AML vitamin E-supplemented (1,000 IU kg^−1^) or control diet (75 IU kg^−1^) and treated the mice with or without gilteritinib for two weeks (days 12–26 post transplant). This dietary vitamin E supplementation increased the amount of vitamin E in the plasma by approximately twofold after two weeks, similar to how vitamin E supplementation (440–1,320 mg per day) increases its plasma levels in humans^45^ (Extended Data Fig. 7g,h). Although vitamin E supplementation did not affect the onset of leukaemogenesis, the two-week gilteritinib regimen significantly extended leukaemia-free survival (Fig. 6f,g). However, the efficacy of gilteritinib was significantly blunted by the vitamin E-supplemented diet (Fig. 6g and Extended Data Fig. 7j). Cox proportional hazard analysis revealed that gilteritinib reduced mortality by AML (Fig. 6h; hazard ratio (HR), 0.22; 95% confidence interval (CI), 0.11–0.47). However, gilteritinib did not significantly reduce the mortality of AML recipient mice that were fed a vitamin E-supplemented diet (HR, 0.79; 95% CI, 0.33–1.89). Immunoblotting confirmed that gilteritinib reduced phosphorylation of FLT3 and GPX4 protein in AML developed in mice fed with vitamin E-supplemented diet (Extended Data Fig. 7i). In a separate experiment, we fed AML recipient mice with control or vitamin E-supplemented diet and then treated them with vehicle or gilteritinib for six consecutive days. Although gilteritinib reduced the frequency of AML cells in the blood, this effect was mitigated in mice that were fed the vitamin E-supplemented diet (Fig. 6i). Consistent with the idea that ferroptosis suppression mitigates the effects of gilteritinib, co-treatment with liproxstatin-1 also reduced gilteritinib efficacy (Fig. 6j and Extended Data Fig. 7k–m). Collectively, these results establish that ferroptosis plays a fundamental role in the therapeutic efficacy of gilteritinib treatment in a preclinical setting, which is dampened by excess uptake of vitamin E from the diet.

To determine whether enhanced Sec recoding is associated with TKI resistance in patients with AML, we analysed the BeatAML2 dataset^46^. Of the 805 samples from patients with AML analysed in this study, 278 samples with matching gilteritinib sensitivity and gene expression data were stratified based on gilteritinib sensitivity and *FLT3* mutational status. Whereas *FLT3*-WT AML did not exhibit differences in the expression levels of genes involved in the selenoprotein biosynthesis pathway, *FLT3*-mutant AML that were resistant to gilteritinib (top 75 percentile) had significantly increased expression of some of these genes, including *SECISBP2*, *SEPSECS* and *RPL30* (Fig. 6k and Extended Data Fig. 7n). These results suggest that upregulation of selenoprotein biosynthesis is associated with FLT3-inhibitor resistance, reinforcing the model that the selenoprotein biosynthesis pathway and ferroptosis evasion renders AML resistant to FLT3 inhibition.

## Discussion

Since the discovery of *FLT3*-ITD mutations in AML in 1996 (ref. ^47^), much effort has been devoted to developing FLT3 inhibitors given the high frequency and poor prognosis associated with these mutations^2^. Although this effort has led to the development and approval of several inhibitors for *FLT3*-mutated AML, these therapies offer limited single-agent efficacy, yield transient responses and invariably result in relapse. More fundamentally, questions remain regarding the role of FLT3 activation in the pathogenesis of AML. Here we identify Sec recoding as one molecular mechanism augmented by active FLT3 that confers AML with ferroptosis resistance (Extended Data Fig. 7o).

The major pathways activated by oncogenic FLT3 include the STAT5A, PI3K–AKT and MEK–MAPK pathways that collectively promote cell growth, cell-cycle progression, differentiation blockade and apoptosis evasion through targets such as MYC, Cyclin D or BIM^48^. Active FLT3 also suppresses transcriptional factors that promote myeloid differentiation, such as C/EBPα and PU.1. In agreement with this, FLT3 inhibition causes myeloid differentiation in patients, rapidly clearing peripheral blood blasts and causing a surge in neutrophils^49–51^. However, this differentiation process is delayed in the marrow and fails to eradicate leukaemia-initiating cells^52^. Adding to the list of cellular responses caused by FLT3 inhibition, we identify ferroptosis as a mechanism by which FLT3 inhibition induces AML cell death. Our current and previous studies establish that oncogenic *FLT3* promotes ferroptosis evasion by upregulating *SEPHS2* expression^16^ and by promoting selenoprotein translation, most notably that of GPX4. FLT3 inhibition reduces the binding of SECISBP2 to the SECIS element of selenoprotein mRNA, the rate-limiting step in recoding UGA stop codons to Sec^15^, thereby reducing selenoprotein translation and increasing lipid peroxidation and ferroptosis. Our results also suggest that upregulation of the selenoprotein biosynthesis pathway, such as by increasing the expression of *SECISBP2*, may contribute to FLT3-inhibitor resistance by mitigating ferroptosis caused by these therapies. However, FLT3 inhibitors may promote ferroptosis through other mechanisms, such as by reducing GSH and GPX4 activity, as previously reported^53^. It is also possible that loss of other selenoproteins, such as TXNRD1 and GPX1, may contribute to ferroptosis induction by FLT3 inhibitors^54,55^.

We demonstrate that the efficacy of gilteritinib treatment against *FLT3*-ITD AML is attenuated by dietary vitamin E. The use of antioxidants or vitamin supplements is common in the general population and in patients with cancer^56,57^. Despite the wide-held perception that these supplements have health benefits, including anticancer and detoxification effects, and do no harm, randomized clinical trials generally have not found beneficial effects of supplements in preventing cancer. Some trials have instead found that supplements including β-carotene, vitamin E and selenium increase the rates of some cancers^58^. Furthermore, given that cytotoxic chemotherapies often engage oxidative stress to kill cancer cells, there is a concern that antioxidants may impede the effectiveness of these therapies^59^. A prospective study in patients with breast cancer treated with a standard breast cancer chemotherapy regimen found that the use of antioxidants, such as vitamins A, C and E, carotenoids and coenzyme Q10, was associated with an increased risk of recurrence^60^. Our study extends these findings and suggests that dietary antioxidants may mitigate the efficacy of not only cytotoxic chemotherapies but also that of some targeted therapies, including TKIs. A broader implication of our study is that multiple TKIs beyond those used to treat haematologic cancers may be rendered ineffective by supplements that impinge on ferroptosis, such as vitamins E and K or coenzyme Q10 (refs. ^35,36,61^). Further clinical studies are warranted before making recommendations regarding the use or avoidance of antioxidants during TKI therapy against AML.

Although previous studies have shown that a TKI, sorafenib, promotes ferroptosis by inhibiting system x_c_^−^ in solid tumour cell lines, this mechanism has recently been challenged^62–65^. Sorafenib is a multi-kinase inhibitor that has activity against Raf, VEGFR, PDGFR, KIT and FLT3, and some of these kinases may be responsible for system x_c_^−^ inhibition^63,66^. Consistent with the finding it causes ferroptosis, we identify sorafenib as a serenib that reduces Sec recoding and GPX4 protein levels in *FLT3*-ITD + AML. The system x_c_^−^ inhibitor erastin, on the other hand, did not affect Sec recoding in AML cells (data not shown), which provides further support for the possibility that sorafenib suppresses Sec recoding in a system x_c_^−^-independent mechanism in AML. Whether sorafenib promotes ferroptosis through different mechanisms (system x_c_^−^ inhibition versus Sec recoding) in a cell type-dependent manner requires further investigation.

## Methods

This research complies with all relevant ethical regulations. All procedures were approved by the Baylor College of Medicine (BCM) Institutional Animal Care and Use Committees (protocol AN-5858). All procedures involving the use of human samples were approved by the BCM Institutional Review Board committee (protocol H-43877). This study did not involve new collection of human specimens. No participant compensation was provided, as it was not applicable to this study.

### Animals

Mice were housed in AAALAC-accredited, specific-pathogen-free animal care facilities at the BCM with 12-h light–dark cycle and received standard chow ad libitum. The mouse alleles used in this study were: *Gpx4*^fl^ (*Gpx4*^tm1.1Qra^/J; JAX, 027964), *Vav1-iCre* (B6.Cg-*Commd10*^Tg(Vav1-icre)A2Kio^/J; JAX, 008610) and *Rosa26*–*CreER* (B6.129-*Gt(ROSA)26Sor*^tm1(cre/ERT2)Tyj^/J; JAX, 008463) on a C57BL/6 background. CD45.1 (B6.SJL-*Ptprc*^a^ *Pepc*^b^/BoyJ; JAX, 002014) and C57BL/6 mice (JAX, 000664) were used as transplant recipient mice. NOD.Cg-*Prkdc*^scid^ *Il2rg*^tm1Wjl^ Tg(CMV-IL-3, CSF2, KITLG)^1Eav/MloySzJ^ (NSG-SGM3) mice (JAX, 013062)^67^ were used for transplanting human AML cells. The mice were fed PicoLab Select Rodent 50 IF/6F 5V5R diet (LabDiet, containing 100 IU kg^−1^ vitamin E) unless otherwise indicated. Vitamin E-supplemented (1,000 IU kg^−1^; TD.170464) and control diet (75 IU kg^−1^; TD.94045), tamoxifen (0.4 g kg^−1^ tamoxifen citrate; TD.130860) and its control diet (TD.07570) were all purchased from Teklad. The mice were fed vitamin E-supplemented diet starting two weeks before transplantation, as we have found that two weeks is sufficient to increase the plasma vitamin E levels by approximately twofold (Extended Data Fig. 7g). Tamoxifen and its control diets were given to mice at the time of AML transplantation. Gilteritinib for in vivo use was formulated in 3% ethanol with 1% Tween-80. The mice were treated with 30 mg kg^−1^ gilteritinib daily by oral gavage. Liproxstatin-1 was dissolved in DMSO, diluted in 1×PBS and administered intraperitoneally (10 mg kg^−1^). Mice of both sexes were used.

### Acute myeloid leukaemia models

The pMIG-MLL-AF9 plasmid and the 3×Ty1- or 3×FLAG-tagged versions were described previously^16,68^. The pMSCV-MLL-AF9-P2A-RFP670 vector was generated by replacing GFP of pMIG-MLL-AF9-3×Ty1 with RFP670, followed by replacing IRES with a P2A sequence by Gibson assembly. Retroviral vectors were packaged by co-transfecting 293T cells with pMIG or other MSCV vectors and pCL-Eco plasmid using Lipofectamine 3000 (ThermoFisher Scientific). Haematopoietic stem and progenitor cells (LSK cells) were sorted and incubated in X-Vivo 15 (Lonza) supplemented with 50 ng ml^−1^ stem cell factor, 50 ng ml^−1^ thrombopoietin, 10 ng ml^−1^ interleukin (IL)-3 and 10 ng ml^−1^ IL-6 (all from Peprotech) for 24 h. After incubation, the cells were spin-infected (600*g* for 30 min at 20 °C) with retroviral supernatant supplemented with polybrene (8 μg ml^−1^) in retronectin (Clontech)-coated plates. The cells (25,000) were transplanted into lethally irradiated C57BL/6 mice. For secondary transplantations, 1–10 × 10^3^ GFP^+^ cells from primary recipient mice were transplanted into sublethally irradiated (600 cGy) recipients. AML cells (1–5 × 10^4^) from *Vav1-NUP98-HOXD13;* *Flt3*^*ITD*^ knock-in mice^43^ were transplanted into sublethally irradiated recipient mice. The AML PDX model^44^, which was derived from a 66-year-old male patient with refractory AML that received previous treatments with azacitidine and midostaurin, was a gift from M. Konopleva. Sanger sequencing identified *FLT3*-ITD, *DNMT3A*, *IDH1* and *NPM1*c mutations. AML PDX cells (1 × 10^6^) were transplanted into sublethally (250 cGy) irradiated NSG-SGM3 mice. Moribund mice were humanely euthanized before reaching experimental end points, in accordance with protocols approved by the IACUC.

### Serum vitamin E measurement

Serum was collected by leaving whole blood at room temperature for 30 min, followed by a 10-min centrifugation at 1,500*g*. Vitamin E levels were measured by a colorimetric assay according to the manufacturer’s protocol (E-BC-K033-S, Elabscience).

### Competitive bone marrow transplantation

Experimental bone marrow cells were mixed with an equal number (5 × 10^5^) of competitor bone marrow cells (CD45.1) and transplanted via the tail vein into lethally irradiated (two doses of 500 cGy) CD45.1 recipient mice. Peripheral blood was collected from the recipient mice at the indicated time point after transplantation and donor chimerism was assessed in Mac-1^+^Gr-1^+^ myeloid cells, B220^+^ B cells and CD3^+^ T cells in the CD45.1^+^ and CD45.2^+^ cell populations by flow cytometry. Secondary transplantation was performed by transplanting 5 × 10^5^ bone marrow cells from primary recipient mice into lethally irradiated secondary recipient mice.

### Flow cytometry and haematopoietic stem and progenitor cell isolation

Bone marrow cells were either flushed from the long bones (tibias and femurs) or isolated by crushing, using a mortar and pestle, the long bones, pelvic bones, vertebrae and sternum in Hank’s buffered salt solution (HBSS) without calcium and magnesium, supplemented with 2% heat-inactivated bovine serum (ThermoFisher Scientific). Splenocytes were dissociated by mashing the entire spleen between frosted slides. Cells were triturated and filtered through a nylon screen (100 μm; Sefar America) or a 48-μm cell strainer (ThermoFisher Scientific) to obtain a single-cell suspension.

Haematopoietic progenitor populations analysed include HSCs (LSK CD150^+^CD48^−/low^), multipotent progenitor populations (LSK CD150^−^CD48^−/low^), haematopoietic progenitor cell 1 (HPC1) (LSK CD150^−^CD48^+^), HPC2 (LSK CD150^+^CD48^+^), GMPs (lineage^−^Sca-1^−^c-kit^+^CD34^+^CD16/32^+^), megakaryocyte erythroid progenitors (lineage^−^Sca-1^−^c-kit^+^CD34^−^CD16/32^−^), common myeloid progenitors (lineage^−^Sca-1^−^c-kit^+^CD34^+^CD16/32^−^) and common lymphoid progenitors (Lin^−^Sca1^low^c-kit^+^IL7-R^+^Flk2^+^). For HSCs, HPC1, HPC2 and multipotent progenitor populations, bone marrow cells were incubated with PE-Cy5-conjugated anti-CD150 (TC15-12F12.2), PE/Cy7-conjugated anti-CD48 (HM48-1), APC-conjugated anti-Sca-1 (Ly6A/E; D7) and APC/Cy7-conjugated anti-c-kit (2B8) in addition to antibodies against the following PE-conjugated lineage markers: Ter-119 (TER-119), B220 (RA3-6B2), Gr-1 (RB6-8C5), CD2 (RM2-5), CD3 (145-2C11) and CD8 (53-6.7). For GMPs, megakaryocyte erythroid progenitors and common myeloid progenitors, cells were incubated with eFluor 660-conjugated CD34 (ThermoFisher, RAM34), PE/Cy7-conjugated CD16/32 (clone 93) and PE/Cy5-conjugated Sca-1 along with the c-kit and lineage markers used for HSCs. For common lymphoid progenitors, PE/Cy5-conjugated CD135 (A2F10) and PE/Cy7-conjugated CD127 (IL-7Ra, A7R34) were stained along with c-kit, Sca-1 and lineage markers used for HSCs. For some experiments, Brilliant Violet 421 (BV421)-conjugated lineage marker antibodies were used. For LSK isolation, bone marrow cells were pre-enriched by selecting c-kit^+^ cells using paramagnetic microbeads and autoMACS (Miltenyi Biotec), and stained using biotin-conjugated c-kit along with APC/Cy7-conjugated streptavidin secondary antibody. All flow cytometry antibodies were acquired from BioLegend if not stated otherwise.

To analyse Mac-1^+^Gr-1^+^ myeloid cells, B220^+^ B cells and CD3^+^ T cells, bone marrow cells were stained with PerCP/Cy5.5-conjugated B220, APC/Cy7-conjugated Mac-1 (M1/70), APC-conjugated CD3 and PE/Cy7-conjugated Gr-1 antibodies.

*MLL-AF9* driven AML cells from bone marrow and spleens were incubated with antibodies for lineage (CD2, CD3, CD8, Gr-1, Ter-119 and B220), anti-Sca-1, anti-c-kit, anti-CD16/32, anti-CD34 and APC-conjugated anti-CD45.2 to isolate L-GMPs (CD45.2^+^lineage^−^Sca-1^−^c-kit^+^CD16/32^+^CD34^+^) as previously described^69^.

To analyse lipid peroxidation levels in L-GMPs, cells were stained with BV421-conjugated lineage markers, biotin-conjugated CD34 developed with AlexFluor700-conjugated streptavidin, BV510-conjugated anti-CD16/32, APC/Cy7-conjugated anti-c-kit and BV785-conjugated anti-Sca-1. The cells were then stained with 2.5 µM BODIPY 581/591 C11 (ThermoFisher Scientific) at 37 °C for 30 min according to the manufacturer’s protocol and washed with PBS. Non-viable cells were excluded from sorts and analyses using the viability dye 4′,6-diamidino-2-phenylindole (1 μg ml^−1^) or LIVE/DEAD far red (Invitrogen). Unless otherwise noted, antibodies were obtained from BioLegend, BD Biosciences or eBioscience. Flow cytometry was performed using FACSAria II, FACSCanto II, LSR II or LSRFortessa flow cytometers (BD Biosciences). Flow cytometry data were analysed using the FACSdiva v8.0.1 (BD Biosciences) and FlowJo v10.10 (LLC) software. Flow cytometry gating strategies are shown in Supplementary Information.

### Cell lines and cell culture

MOLM-13, THP-1, Kasumi-1, OCI-AML3, K562, NB4 and MV4-11 cell lines were cultured in RPMI-1640 medium supplemented with 10% fetal bovine serum (FBS) and 1% penicillin–streptomycin (ThermoFisher Scientific). HL-60 cells were cultured in IMDM medium containing 20% FBS and 1% penicillin–streptomycin. OCI-AML2 cells were cultured in αMEM with 10% FBS and 1% penicillin–streptomycin. Unless otherwise noted, 40 nM sodium selenite was added to the media. Leukaemia cell lines were acquired from M. Goodell, R. Rau, D. Lacorazza or the American Type Culture Collection. HEK293T cells (American Type Culture Collection) and HepG2 cells (from S. Hartig) were cultured in DMEM medium containing 10% FBS and 1% penicillin–streptomycin. PDX cells were cultured in X-Vivo 10 (Lonza) with 20% BIT9500 (Stem Cell Technologies); human stem cell factor, IL-3, FLT3L and IL-6 (10 ng ml^−1^ each; Peprotech) and 1% penicillin–streptomycin (Gibco). Mouse LSK cells were sorted and cultured in X-Vivo 15 (Lonza) supplemented with 50 ng ml^−1^ mouse stem cell factor, 50 ng ml^−1^ mouse thrombopoietin, 10 ng ml^−1^ mouse IL-3 and 10 ng ml^−1^ mouse IL-6 (all from Peprotech). Murine AML cells were cultured in RPMI-1640 medium containing 10% FBS, 1% penicillin–streptomycin and 10 ng ml^−1^ mouse IL-3. The cells were cultured at 37 °C with 5% CO_2_ and 20% O_2_. Gilteritinib (MedChemExpress, HY-12432), ponatinib (Apex, catalogue number 11494), quizartinib (MedChemExpress, HY-13001), trametinib (Cayman Chemical, catalogue number 16292), (1S,3R)-RSL-3 (Cayman Chemical, catalogue number 19288), deferoxamine (mesylate) (Cayman Chemical, catalogue number 14595) and liproxstatin-1 (MedChemExpress, HY-12726) were dissolved in tissue-culture-grade DMSO.

### Vector construction, gene transduction and CRISPR–Cas9-mediated gene ablation

The codons that encode cysteine 49 or 71 of EGFP were substituted to Sec (UGA; referred to as C49U and C71U) and cloned into pCS2 or pLeGO-iC2 (Addgene, catalogue number 27345), followed by the SECIS element of human *GPX4, GPX1* or *SEPHS2*. Control vectors were generated by cloning an intact EGFP, followed by SECIS elements. FLAG-tagged human *SECISBP2* sequence with or without the C691R mutation (synthesised by Integrated DNA Technologies) and human *FSP1* complementary DNA were cloned into a MSCV-IRES-RFP670 vector. The pCW107-MEK1-S218/222D vector, to express constitutively active MEK1, was obtained from Addgene (catalogue number 64604). Lentiviral vectors were packaged using psPAX2 (Addgene, catalogue number 12260) and pMD2.G (Addgene, catalogue number 12259), whereas retroviral vectors were packaged using pCL-Eco. Single clones were picked for MOLM-13 cells expressing EGFP^C49U^-SECIS^GPX4^ (referred to as SecGR cells) and control EGFP for all downstream experiments including the drug screening. Single guide RNA sequences (Supplementary Table 4) were cloned into the pKLV2-U6gRNA5(BbsI)-PGKpuro2ABFP-W vector (Addgene, catalogue number 67974) and packaged into lentiviral vectors as described earlier. Cells expressing Cas9 were infected with sgRNA lentiviral particles and BFP expression were monitored by flow cytometry.

### CRISPR–Cas9-mediated homology-directed repair

MOLM-13 cells were electroporated with the RNP complex containing Cas9 protein (PNA Bio), sgRNA and homology-directed repair template (Integrated DNA Technologies). The sgRNA sequences are provided in Supplementary Table 4. Electroporation was done using the Neon transfection system (Invitrogen) with 1,350 V, 20 ms, one pulse. Sequences surrounding the F691 codon were PCR amplified and Sanger sequenced to verify successful editing to F691L.

### Competitive culture assay

MOLM-13 cells were transduced with a *FSP1*-expressing retroviral vector that co-expresses RFP670. The fraction of RFP670-expressing cells was analysed four days after infection, followed by DMSO or gilteritinib treatment for 48 h. The frequencies of RFP670-expressing cells were monitored by flow cytometry.

### Drug screening

Approved Oncology Drugs Set X (plates 4893–4895) was obtained from the National Cancer Institute. SecGR cells were plated into 96-well plates (10,000 cells per well) and exposed to the drugs (final concentration of 500 nM) in triplicate. We included eight wells of DMSO and eight wells of DMSO plus 5 µM sodium selenite as controls. After 24 h of drug treatment, the cells were analysed using the BD LSR II or Canto II flow cytometers equipped with High Throughput Sampler attachments. GFP^+^ cell frequencies for each well were normalized to the *z*-scores.

$$z{\text -}{\mathrm{score}}=\frac{\mathrm{{sample}-{negative}\,{control}\,{mean}}}{\mathrm{{standard}\,{deviation}\,{of}\,{negative}\,{control}}}$$

### Quantitative PCR

RNA was isolated using TRIzol (ThermoFisher Scientific) according to the manufacturer’s instructions. Random primers and a SuperScript VILO cDNA synthesis kit (ThermoFisher Scientific) were used to generate cDNA. Quantitative PCR was performed using SYBR Premium Ex Taq (Tli RNaseH Plus) ROX Plus kits (Clontech) on a ViiA7 real-time PCR system (ThermoFisher Scientific). Each sample was normalized to β-actin. Data were analysed using the 2^−ΔΔCt^ method. Primers to quantify cDNA levels are listed in Supplementary Table 4.

### RNA-immunoprecipitation assay

AML cells for RNA-IP were treated with the respective drugs and 40 nM sodium selenite. The cells were harvested 24 h later for immunoprecipitation. 293T cells were transiently transfected with an empty vector, 3×FLAG-SECISBP2 or 3XFLAG-SECISBP2-C691R and collected 48 h later. The cells were lysed in IP lysis buffer (25 mM Tris–HCl pH 7.5, 150 mM NaCl, 1 mM EDTA, 1% NP-40 and 5% glycerol) and clarified by centrifugation. The cleared lysate was immunoprecipitated using protein-A magnetic beads (NEB) complexed with anti-SECISBP2 (Proteintech, 12798-1-AP), anti-FLAG M2 magnetic beads (MilliporeSigma) or control IgG for 2 h at 4 °C. The beads were washed five times with the IP lysis buffer. After the last wash, TRIzol was added to the beads–immunocomplex for RNA isolation. Reverse transcription and quantitative PCR were performed as described earlier. All samples were amplified with the same numbers of PCR cycles. The primers used are listed in Supplementary Table 4. RIP-seq was performed by reverse transcribing and amplifying the co-precipitated RNA according to the SMART-Seq2 protocol. Amplified cDNA was fragmented and barcoded using Nextera XT DNA Library Preparation Kit (Illumina) and sequenced on the NovaSeq X Plus platform (Illumina).

### Analysis of RNA-immunoprecipitation sequencing data

Sequencing reads were mapped to the GRCh38 human reference genome after adaptor trimming and filtering out low quality bases. The data processing, normalization and enrichment analyses were performed using DESeq2. Gene set enrichment analysis was performed using the GSEA package available in R (version 4.4.3). The gene set enrichment analysis terms for selenoproteins were curated to include the 25 known human selenoproteins to directly assess for enrichment in IP samples.

### SELENOP uptake assays

Human *SELENOP* cDNA with ten Sec-to-Cys mutations (SELENOP^UC^) was synthesised by Integrated DNA Technologies and cloned in pcDNA3. A 2×Ty1 tag was cloned after the signal sequence as reported previously^40^. The pcDNA3-2×Ty1-SELENOP^UC^ plasmid was transfected into 293T cells. The cells were cultured for 48 h in a serum-free RPMI medium and the 2×Ty1-SELENOP^UC^-containing conditioned medium was collected. MOLM-13 cells were then cultured in 2×Ty1-SELENOP^UC^-containing conditioned medium supplemented with 10% FBS for 3 h before immunoblotting. The cells were pretreated with FLT3 inhibitors for 20 h before exposure to the 2×Ty1-SELENOP^UC^-containing conditioned medium.

### Preparation of SELENOP-containing conditioned media

SELENOP-containing conditioned media were prepared according to a previous study^70^. Briefly, HepG2 cells cultured on 10-cm dish until they reached 70–80% confluency were serum-starved for 12–24 h. The medium was then changed to serum-free RPMI containing 100 nM sodium selenite to induce SELENOP expression, as reported previously^70^. Conditioned medium was collected after 48 h and proteins were enriched using an Amicon Ultra-15 column (30-kDa molecular weight cutoff, UFC9030).

### Immunoblotting

Cells or tissues were homogenized and incubated with 10% trichloroacetic acid on ice for 15 min before centrifugation. The resulting pellets were washed twice with ice-cold acetone, dried and solubilized with a solubilization buffer (9 M urea, 2% Triton X-100 and 1% dithiothreitol), followed by the addition of an LDS loading buffer (ThermoFisher Scientific) as previously described^71^. Proteins were separated on a bis-Tris polyacrylamide gel and transferred to a polyvinylidene fluoride membrane. Antibodies used for immunoblotting include those raised to SEPHS2 (1:1,000; ThermoFisher Scientific, PA5-27950), EEFSEC (1:1,000; ThermoFisher Scientific, PA5-31764), GPX4 (1:4,000; Abcam, ab125066), GAPDH (1:5,000; Cell Signaling Technology, catalogue number 2118), phospho-FLT3 (Tyr589/591) (1:1,000; Cell Signaling Technology, catalogue number 3464), FLT3 (1:1,000; Cell Signaling Technology, catalogue number 3462), phospho-c-Abl (1:1,000; Cell Signaling Technology, catalogue number 2865), c-Abl (1:1,000; Cell Signaling Technology, catalogue number 2862), TXNRD1 (1:1,000; Proteintech, 11117-1-AP), GPX1 (1:1,000; ThermoFisher Scientific, JJ092-07), MSRB1 (1:1,000; Proteintech, 15333-1-AP), SELENON (1:200; Santa Cruz, sc-365824), FSP1/AIFM2 (1:1,000; Proteintech, 20886-1-AP), LRP8 (1:1,000; Invitrogen, MA5-38208), p-p44/42 (1:1,000; Cell Signaling Technology, 4370S), p44/42 (1:1,000; Cell Signaling Technology, 4695S), MEK1 (1:1,000; Cell Signaling Technology, 9124S), p-c-ABL (1:1,000; Cell Signaling Technology, 2865S), Ty1 (1:1,000; ThermoFisher Scientific, MA5-23513), SLC7A11 (1:1,000; Cell Signaling Technology, 12691S) and β-actin (1:5,000; MilliporeSigma, AC-15, A5441).

### ICP-MS analysis

MOLM-13 cells (5 × 10^6^) were cultured with or without sodium selenite and gilteritinib, pelleted and washed with PBS. The cell pellets were solubilized with 67–70% nitric acid (OmniTrace, Sigma, NX0407) for 2 h, diluted to a sufficient volume to permit analysis and analysed on the 8900 ICP-QQQ-MS system.

### BeatAML2 analysis

Data of 805 patients with AML from the BeatAML2 dataset were used for further analysis^46^. Among the available drug datasets, 287 samples had area-under-the-curve data for gilteritinib; 278 of these also had matching gene expression data. Following the approach in the original studies^46,72^, we defined drug resistance using a percentile cutoff. Although the original study used 80th percentile as the cutoff, we chose 75th percentile to improve the balance of group sizes and to increase statistical power. This resulted in 70 samples classified as gilteritinib-resistant and 207 as non-resistant. Raw count gene expression data were normalized as transcripts per million, transformed by log(*x* + 1) and then standardized by the *z*-score. Because gilteritinib targets FLT3, we performed a stratified analysis based on *FLT3* mutation status. Within each FLT3 subgroup (FLT3 mutation-positive or -negative), we compared the expression of genes involved in selenoprotein biosynthesis between resistant and non-resistant samples using a two-sided Student’s *t*-test. Before performing the Student’s *t*-test, we used Shapiro–Wilk test to check the normality of the standardized data, confirming that they followed an approximately normal distribution. *P* < 0.05 was considered statistically significant.

### Statistics and reproducibility

Statistical analyses were performed using GraphPad Prism 10 (GraphPad Software). Survival analyses were performed using the survival package in R (version 4.4.3). Survival comparisons were assessed using a log-rank test with *P* values adjusted for multiple comparisons in Fig. 6f using the Benjamini–Hochberg method. The Cox proportional hazards regression model and general linear hypothesis testing for interaction effect were used to estimate the HR and 95% CI values. The primary outcome was survival time. The model included treatment (vehicle versus gilteritinib), diet (control versus vitamin E-supplemented) and interaction term (treatment × diet) to assess the effect on survival. Animals were assigned to treatment groups based on computer-generated random numbers. To ensure the reproducibility of our findings, data were derived from multiple independent experiments performed on different days. Sample sizes were chosen based on observed effect sizes and standard errors from previous experiments. Data were assumed to be normally distributed with equal variance across groups but this was not formally tested. The statistical details and sample number (*n*) of experiments are described in the Figure legends. *P* ≤ 0.05 was considered significant. The animal studies were conducted in a non-blinded manner. The investigators were not blinded to the conditions of the experiments during data collection and analysis. No data points were excluded from analyses.

### Reporting summary

Further information on research design is available in the Nature Portfolio Reporting Summary linked to this article.

## Online content

Any methods, additional references, Nature Portfolio reporting summaries, source data, extended data, supplementary information, acknowledgements, peer review information; details of author contributions and competing interests; and statements of data and code availability are available at https://doi.org/10.1038/s41556-026-02016-5.

## Supplementary information

Supplementary Information Supplementary Figs. 1–3.

Reporting Summary

Supplementary Tables 1–4. Supplementary Table 1. Drug screening results with the SecGR cell line. Supplementary Table 2. RIP-seq of differentially expressed genes. Supplementary Table 3. BeatAML patient data analysis for selenoprotein synthesis pathway. Supplementary Table 4. Primers used in the study.

## Source data

Source Data Fig. 1 Unprocessed western blots or gels.

Source Data Fig. 1 Statistical source data.

Source Data Fig. 2 Statistical source data.

Source Data Fig. 3 Unprocessed western blots or gels.

Source Data Fig. 3 Statistical source data.

Source Data Fig. 4 Unprocessed western blots or gels.

Source Data Fig. 4 Statistical source data.

Source Data Fig. 5 Unprocessed western blots or gels.

Source Data Fig. 5 Statistical source data.

Source Data Fig. 6 Unprocessed western blots or gels.

Source Data Fig. 6 Statistical source data.

Source Data Extended Data Fig. 1 Unprocessed western blots or gels.

Source Data Extended Data Fig. 1 Statistical source data.

Source Data Extended Data Fig. 2 Unprocessed western blots or gels.

Source Data Extended Data Fig. 2 Statistical source data.

Source Data Extended Data Fig. 3 Unprocessed western blots or gels.

Source Data Extended Data Fig. 3 Statistical source data.

Source Data Extended Data Fig. 4 Unprocessed western blots or gels.

Source Data Extended Data Fig. 4 Statistical source data.

Source Data Extended Data Fig. 5 Unprocessed western blots or gels.

Source Data Extended Data Fig. 5 Statistical source data.

Source Data Extended Data Fig. 6 Unprocessed western blots or gels.

Source Data Extended Data Fig. 6 Statistical source data.

Source Data Extended Data Fig. 7 Unprocessed western blots or gels.

Source Data Extended Data Fig. 7 Statistical source data.

## Data Availability

The RIP-seq data supporting the findings in this study have been deposited in the Gene Expression Omnibus (GEO) under accession code GSE325271. Source data are provided with this paper.
