# Supplementary material for: Targeting oncogenic FLT3 uncovers a ferroptosis vulnerability through selenocysteine recoding in acute myeloid leukaemia
Source: Nat Cell Biol. 2026 Aug 7;28(8):1715–27. doi: 10.1038/s41556-026-02016-5 (PMC13457094; doi:10.1038/s41556-026-02016-5)

# Extended Data Figure 1

## Ext. Data. Fig. 1C

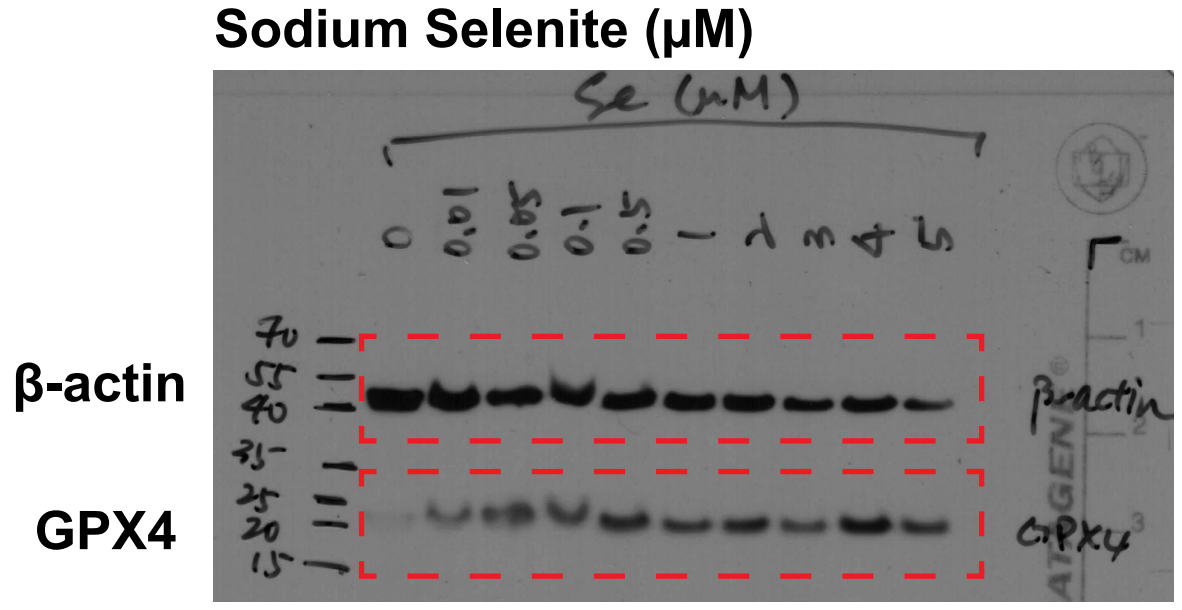


## Ext. Data. Fig. 1D

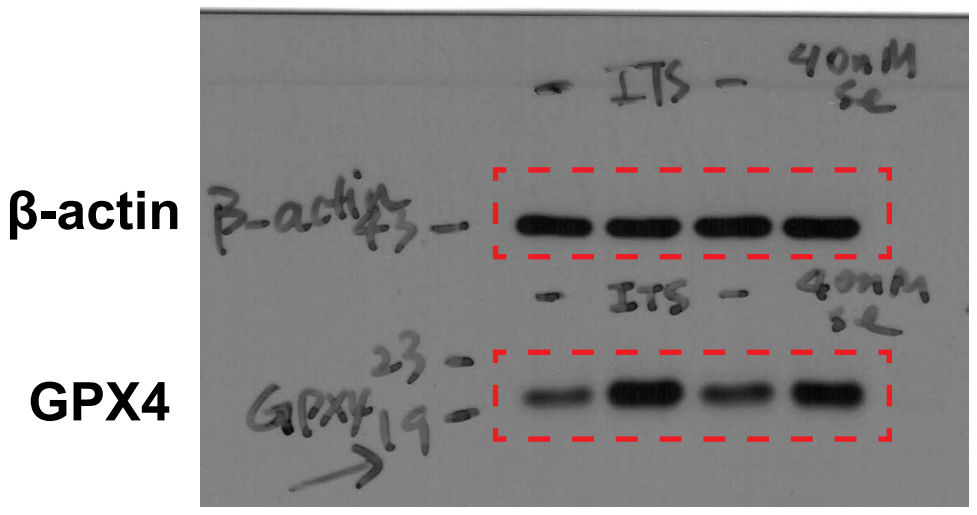


## Ext. Data. Fig. 1E

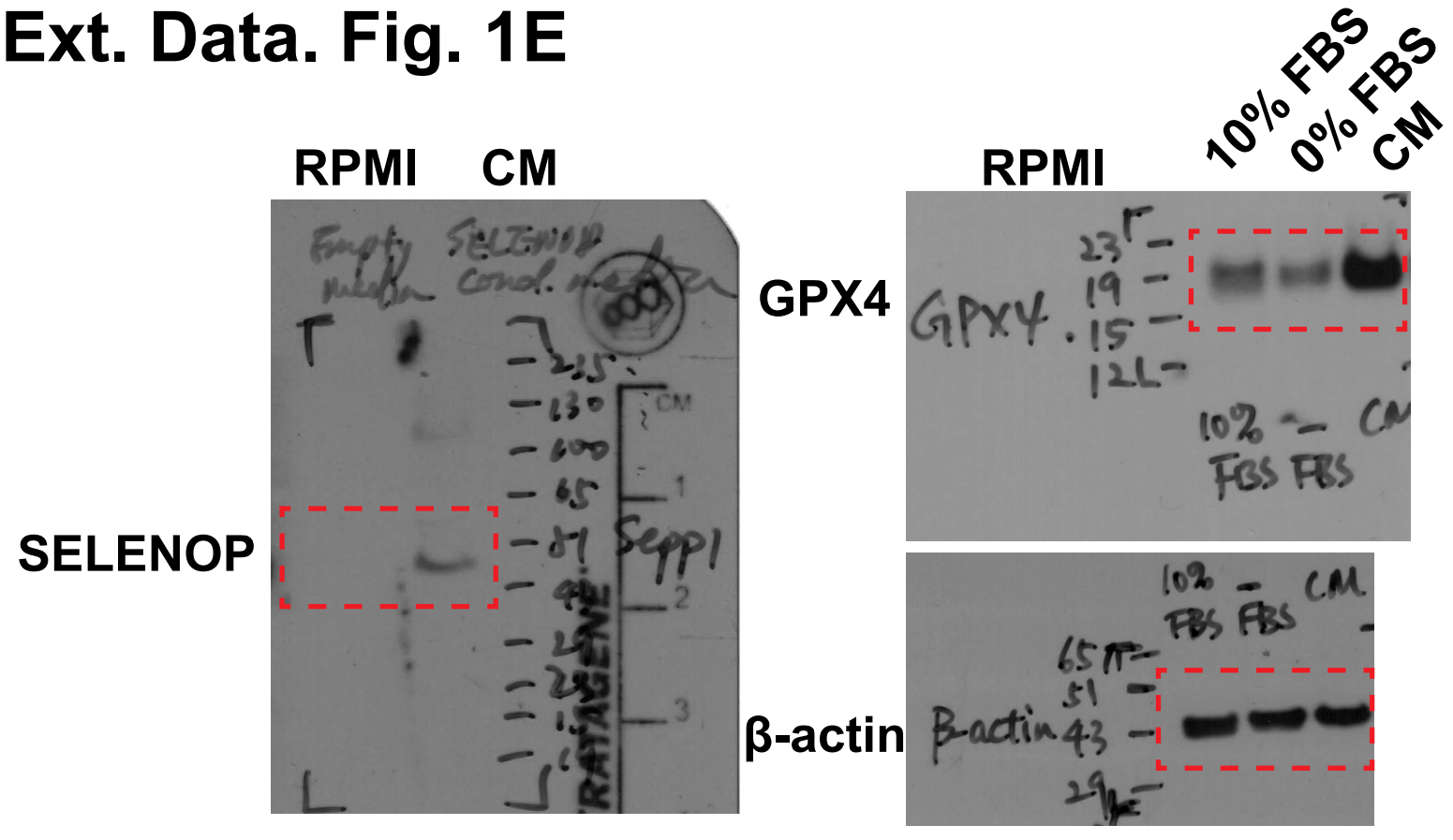


## Ext. Data. Fig. 1F

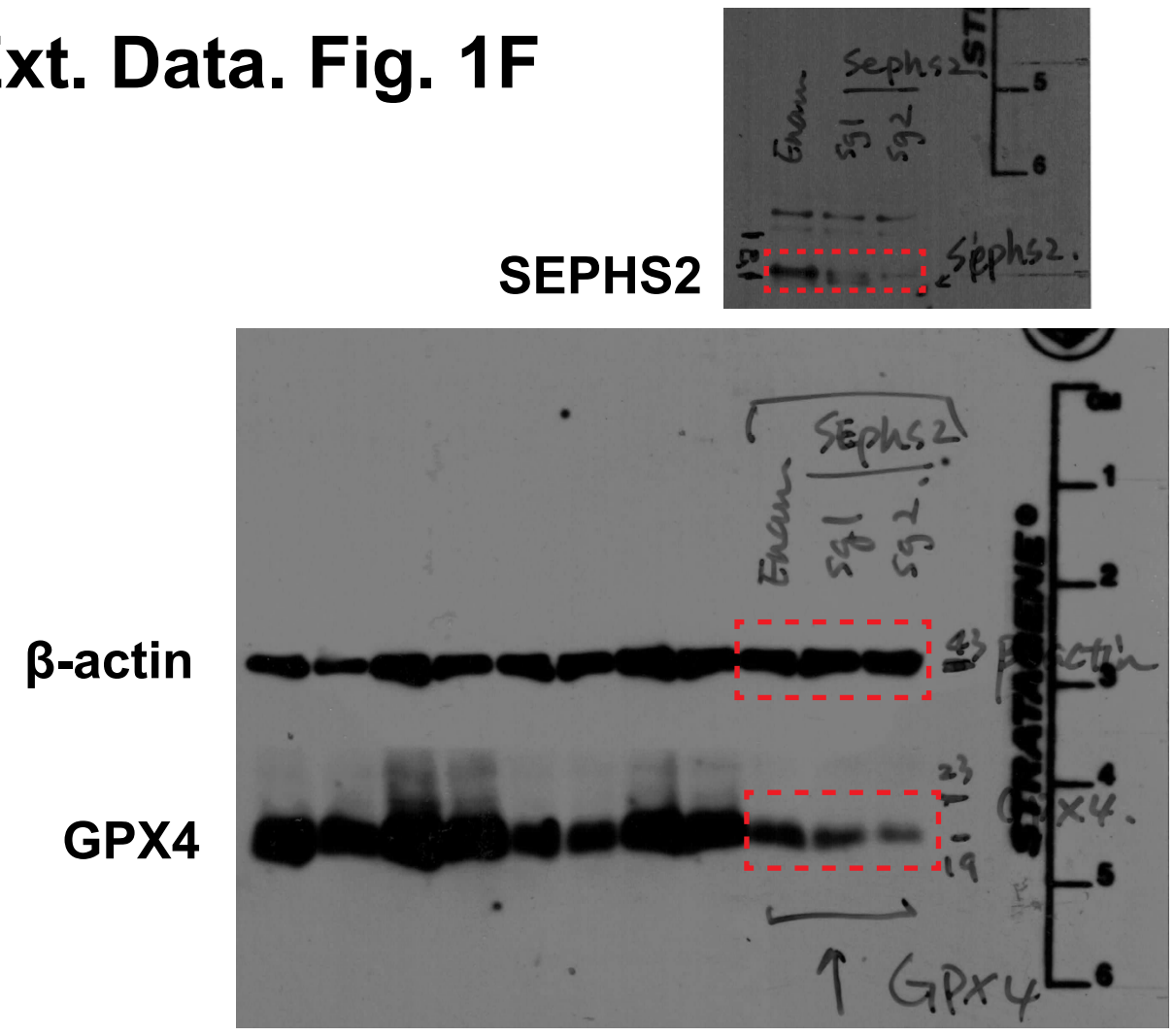


## Ext. Data. Fig. 1G

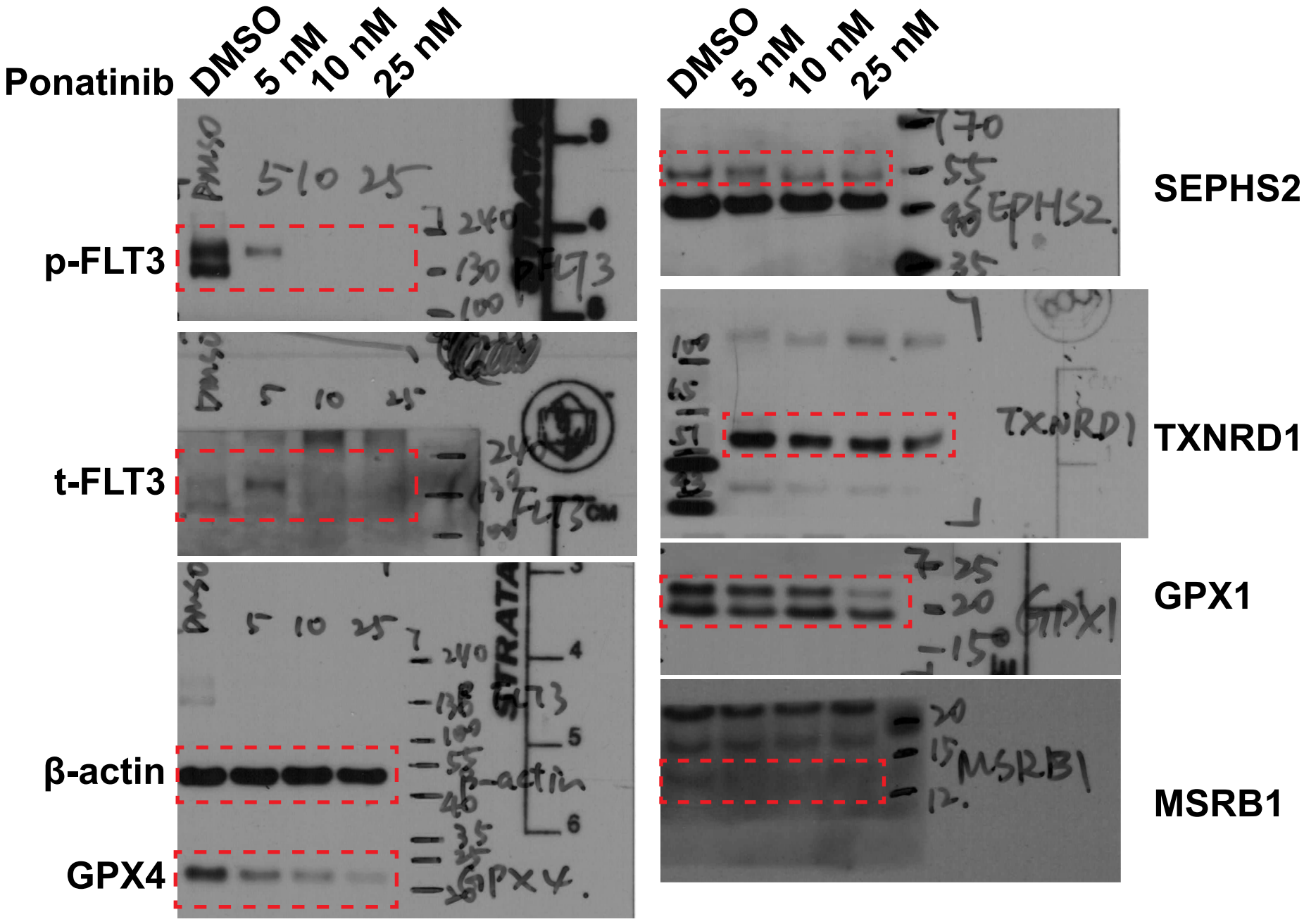


## Ext. Data. Fig. 1H

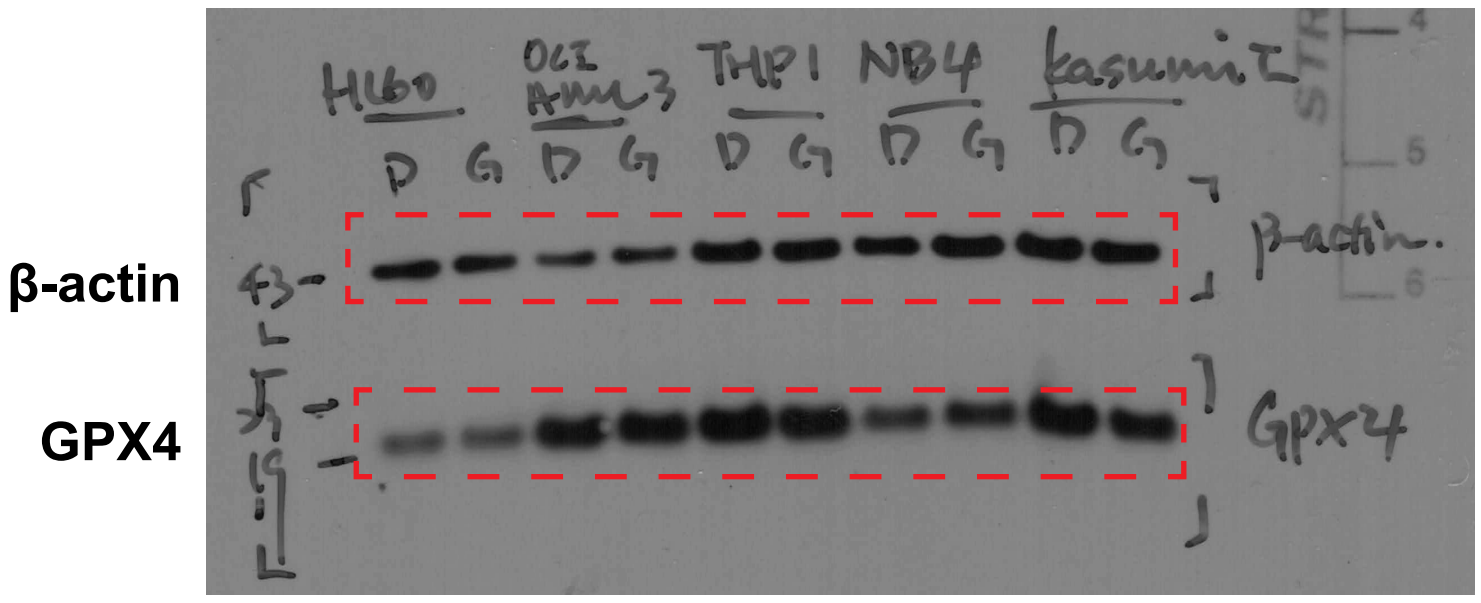


D = DMSO treated
G = Gilteritinib treated

# Extended Data Figure 1 (cont.)

## Ext. Data. Fig. 1I

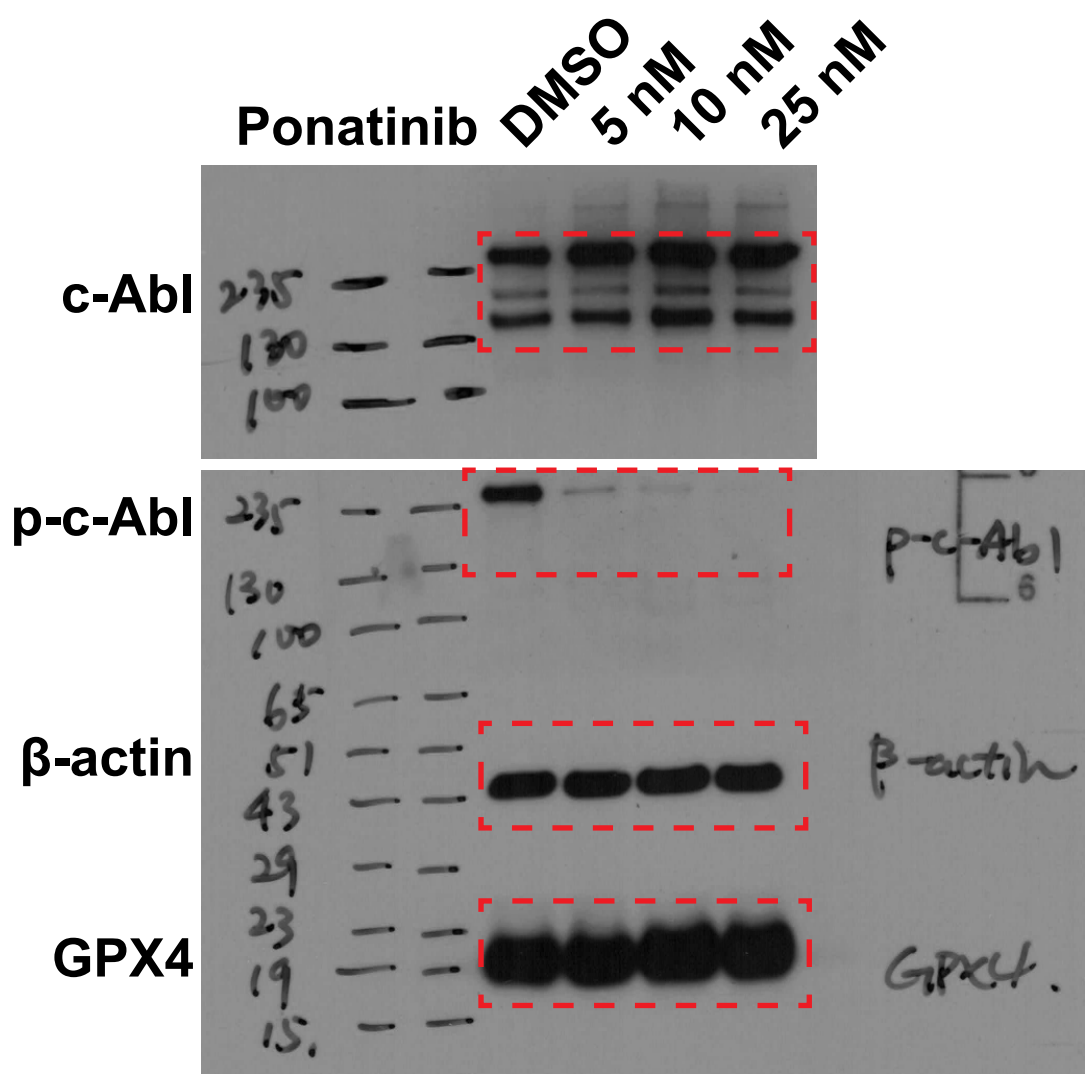


## Ext. Data. Fig. 1L

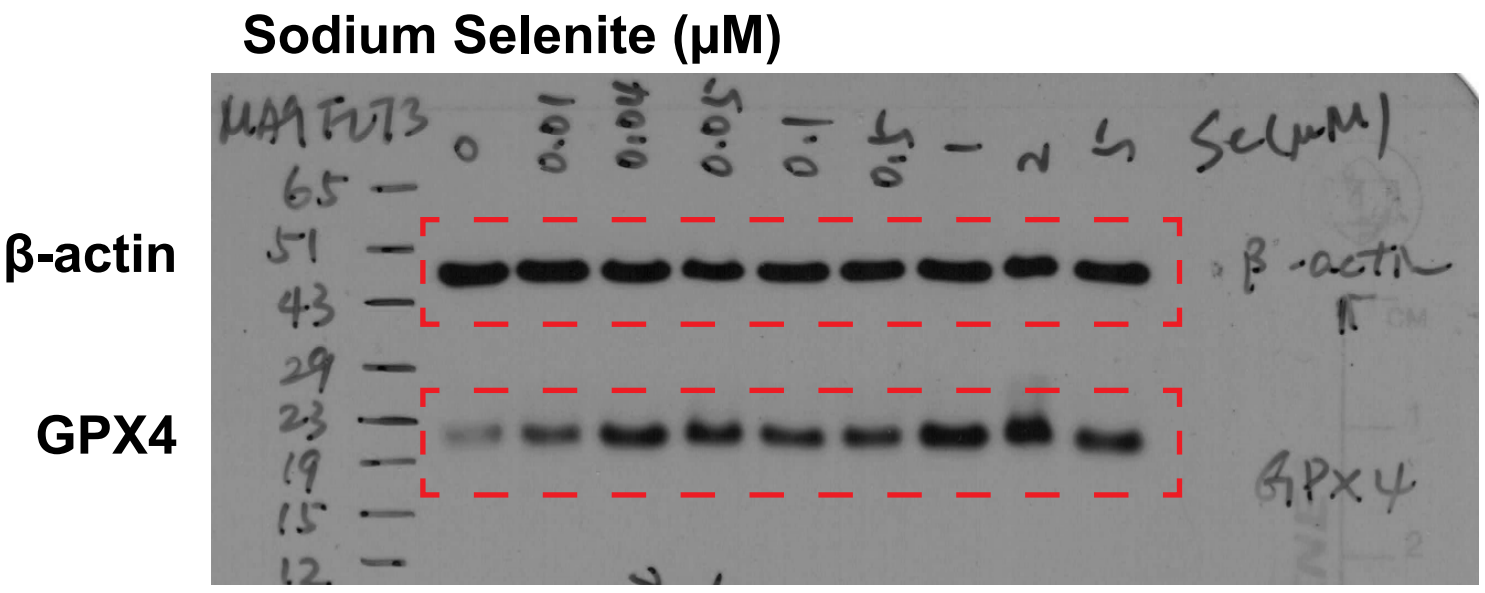


## Ext. Data. Fig. 1M

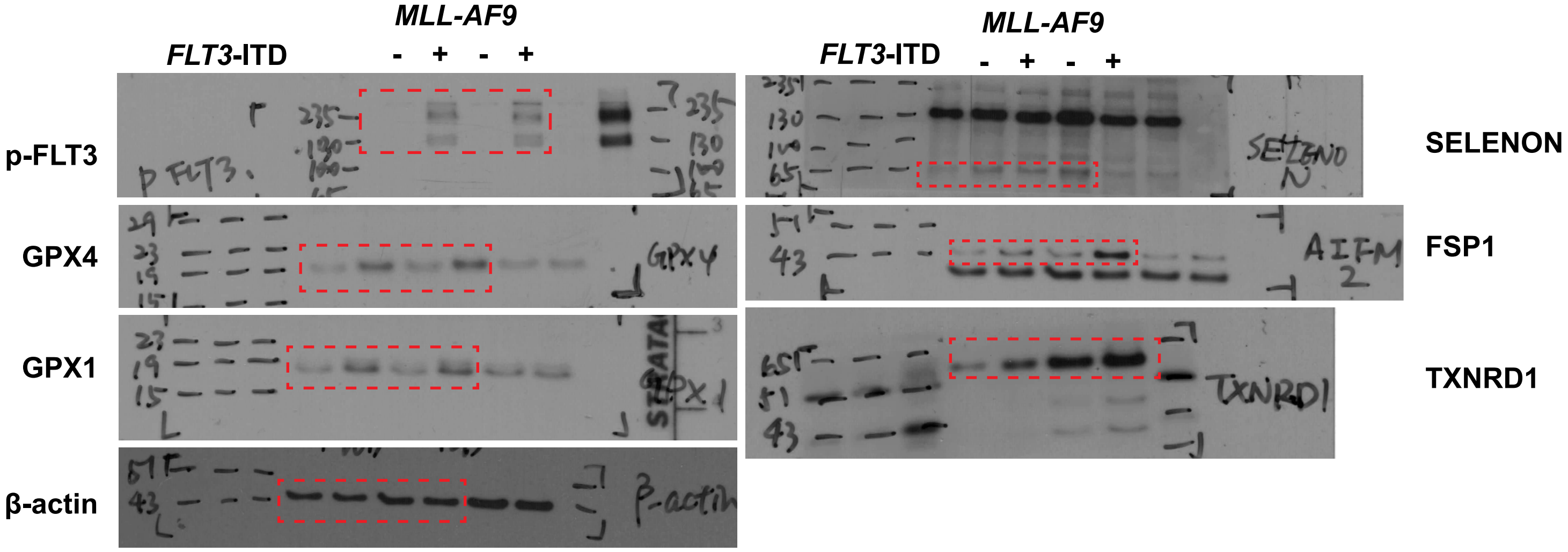

Supplement: Supplementary file 3 [file 41556_2026_2016_MOESM15_ESM.pdf]
