# Supplementary material for: Targeting oncogenic FLT3 uncovers a ferroptosis vulnerability through selenocysteine recoding in acute myeloid leukaemia
Source: Nat Cell Biol. 2026 Aug 7;28(8):1715–27. doi: 10.1038/s41556-026-02016-5 (PMC13457094; doi:10.1038/s41556-026-02016-5)
Supplement: Supplementary file 6 [file 41556_2026_2016_MOESM1_ESM.pdf]

nature cell biology

Supplementary information

https://doi.org/10.1038/s41556-026-02016-5

Targeting oncogenic FLT3 uncovers a ferroptosis vulnerability through selenocysteine recoding in acute myeloid leukaemia

In the format provided by the authors and unedited

Supplemental information

Targeting oncogenic FLT3 uncovers a ferroptosis vulnerability through selenocysteine recoding in acute myeloid leukemia

Minhua Li, Yudan Zhu, Yuki Kageyama, Ken Furudate, Ayumi Kitano, Taotao Tan, Mengdie Feng, Jing Zhou, Tao Wang, Robert J. Taylor, Alexandra M. Stevens, Md. Abul Hassan Samee, Jeffrey A. Magee, Koichi Takahashi, and Daisuke Nakada

Inventory of Supplemental Tables

Supplementary Table 1: Drug screening results with the SecGR cell line.

Supplementary Table 2: RNA-IP sequencing (RIP-seq) differentially expressed genes

Supplementary Table 3: BeatAML patient data analysis for selenoprotein synthesis pathway.

Supplementary Table 4: Primers used in the study.

Supplementary Figures

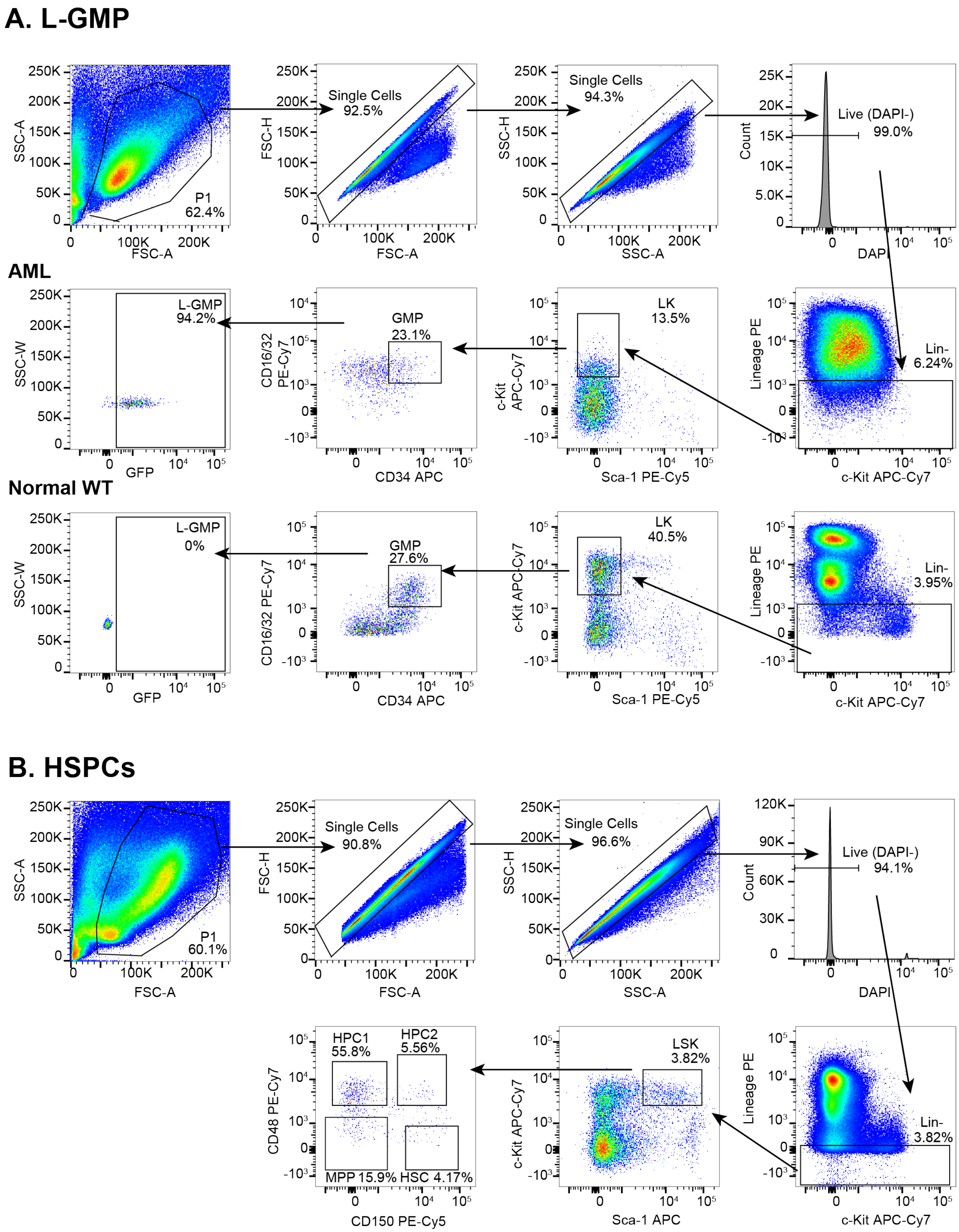

Supplementary Figure 1. L-GMP and hematopoietic stem and progenitor cells (HSPCs) gating strategy. A. L-GMP. **B.** HSPCs.

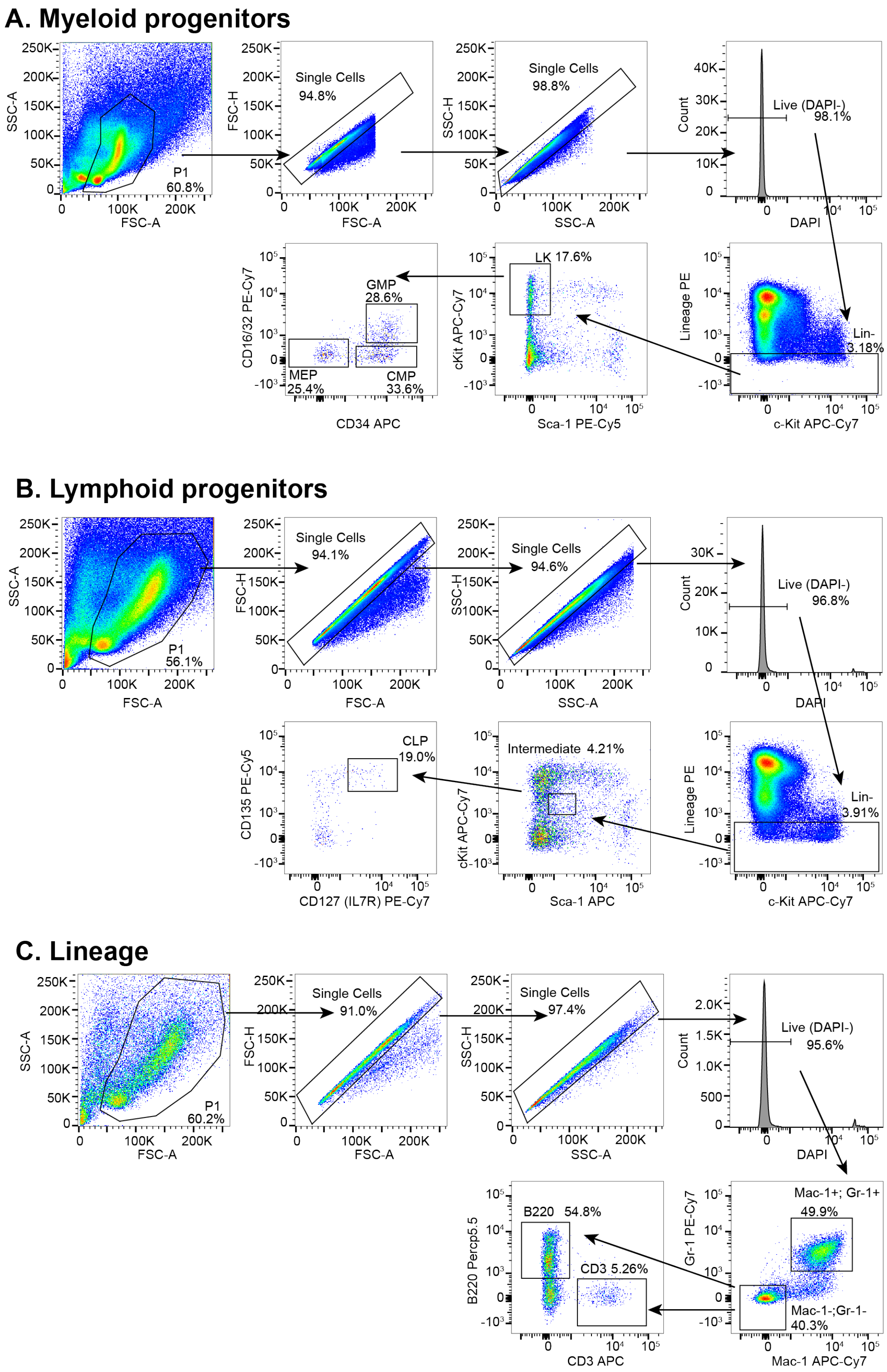

Supplementary Figure 2. Myeloid, lymphoid progenitors, and lineage populations gating strategy. A. Myeloid progenitors. **B.** Lymphoid progenitors. **C.** Lineage.

A. Competitive transplantation

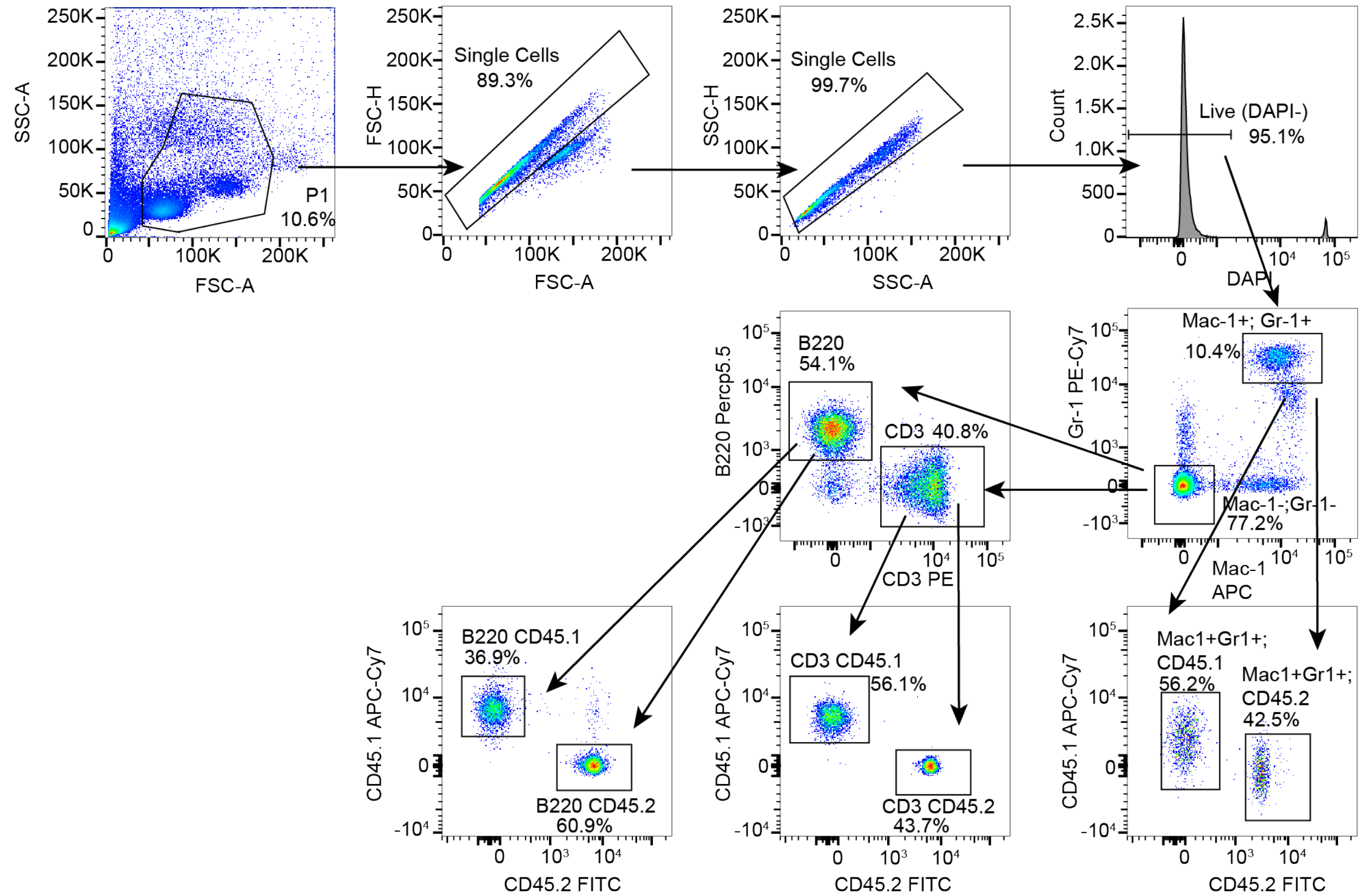

Supplementary Figure 3. Competitive transplant recipient peripheral blood chimerism gating strategy.

Gating strategy to identify Mac-1$^+$Gr-1$^+$ myeloid cells, B220$^+$ B-cells, and CD3$^+$ T-cells within CD45.2$^+$ recipient type and CD45.1$^+$ donor type cells in competitive bone marrow transplantation assays.
