# Supplementary material for: Targeting oncogenic FLT3 uncovers a ferroptosis vulnerability through selenocysteine recoding in acute myeloid leukaemia
Source: Nat Cell Biol. 2026 Aug 7;28(8):1715–27. doi: 10.1038/s41556-026-02016-5 (PMC13457094; doi:10.1038/s41556-026-02016-5)

Extended Data Figure 5

Ext. Data. Fig. 5C

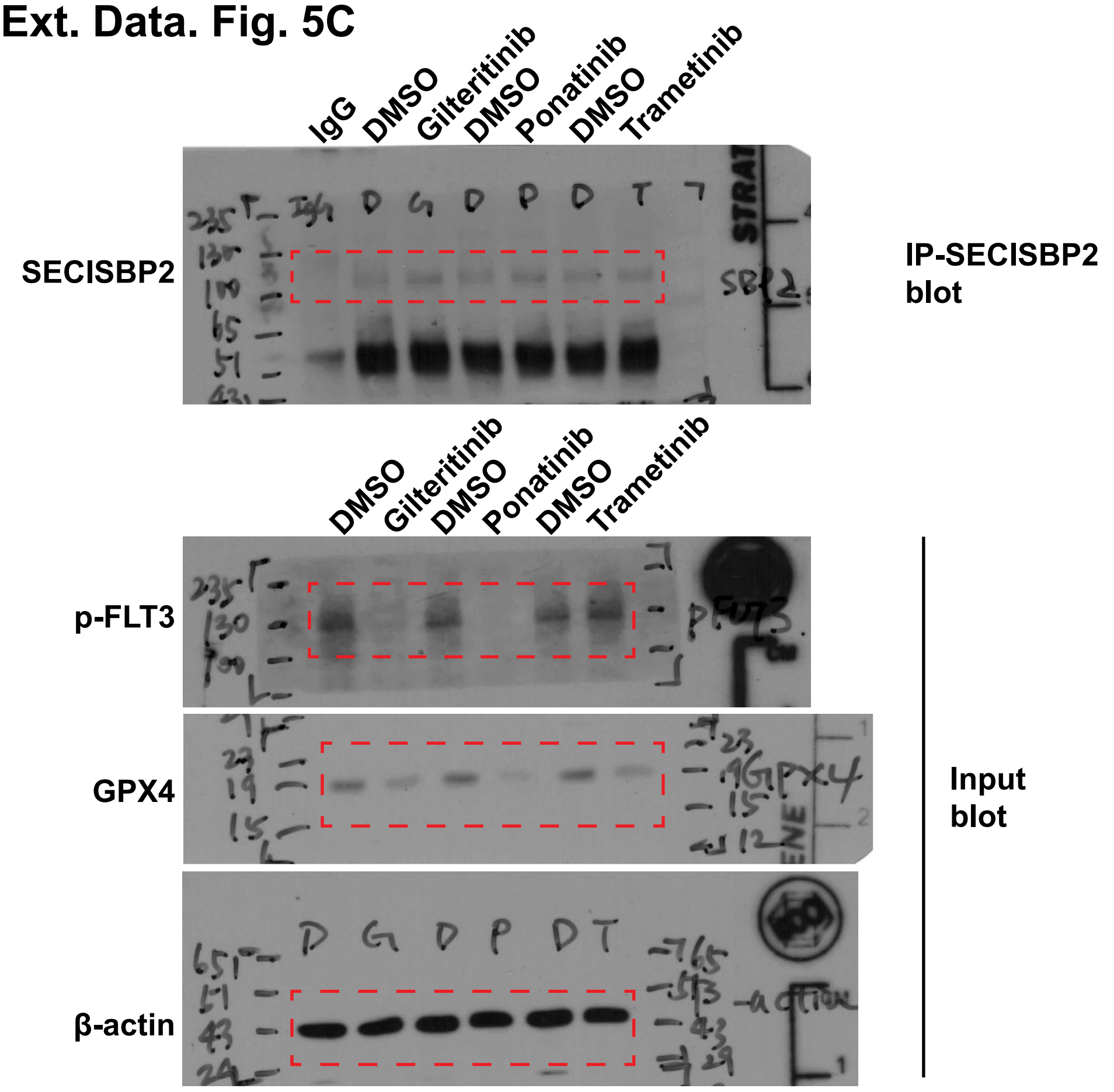

Supplement: Supplementary file 8 [file 41556_2026_2016_MOESM23_ESM.pdf]
