# Supplementary material for: Targeting oncogenic FLT3 uncovers a ferroptosis vulnerability through selenocysteine recoding in acute myeloid leukaemia
Source: Nat Cell Biol. 2026 Aug 7;28(8):1715–27. doi: 10.1038/s41556-026-02016-5 (PMC13457094; doi:10.1038/s41556-026-02016-5)
Supplement: Supplementary file 10 [file 41556_2026_2016_MOESM27_ESM.pdf]

Extended Data Figure 7

Ext. Data. Fig. 7D

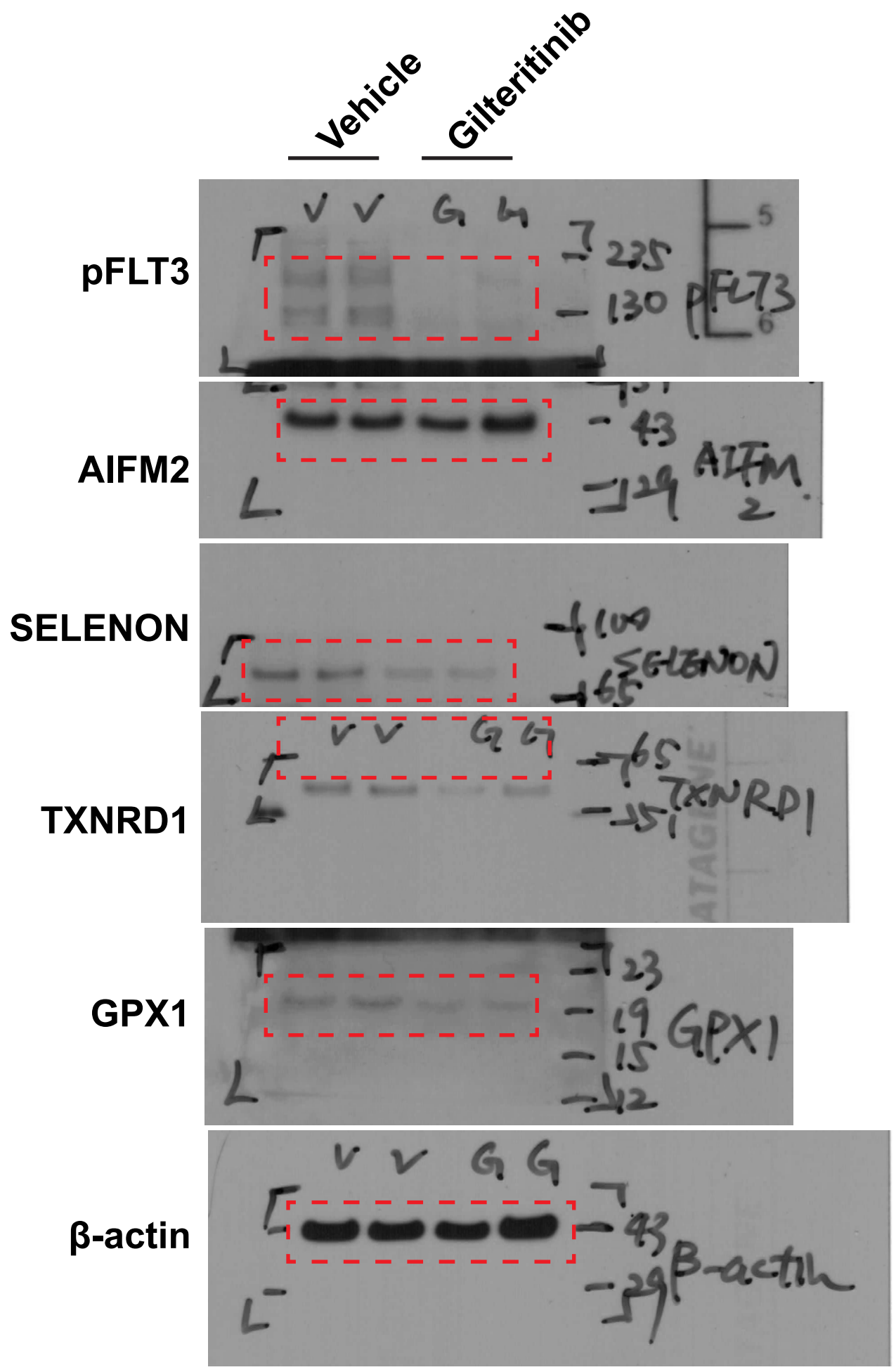

MLL-AF9; FLT3-ITD

Ext. Data. Fig. 7E

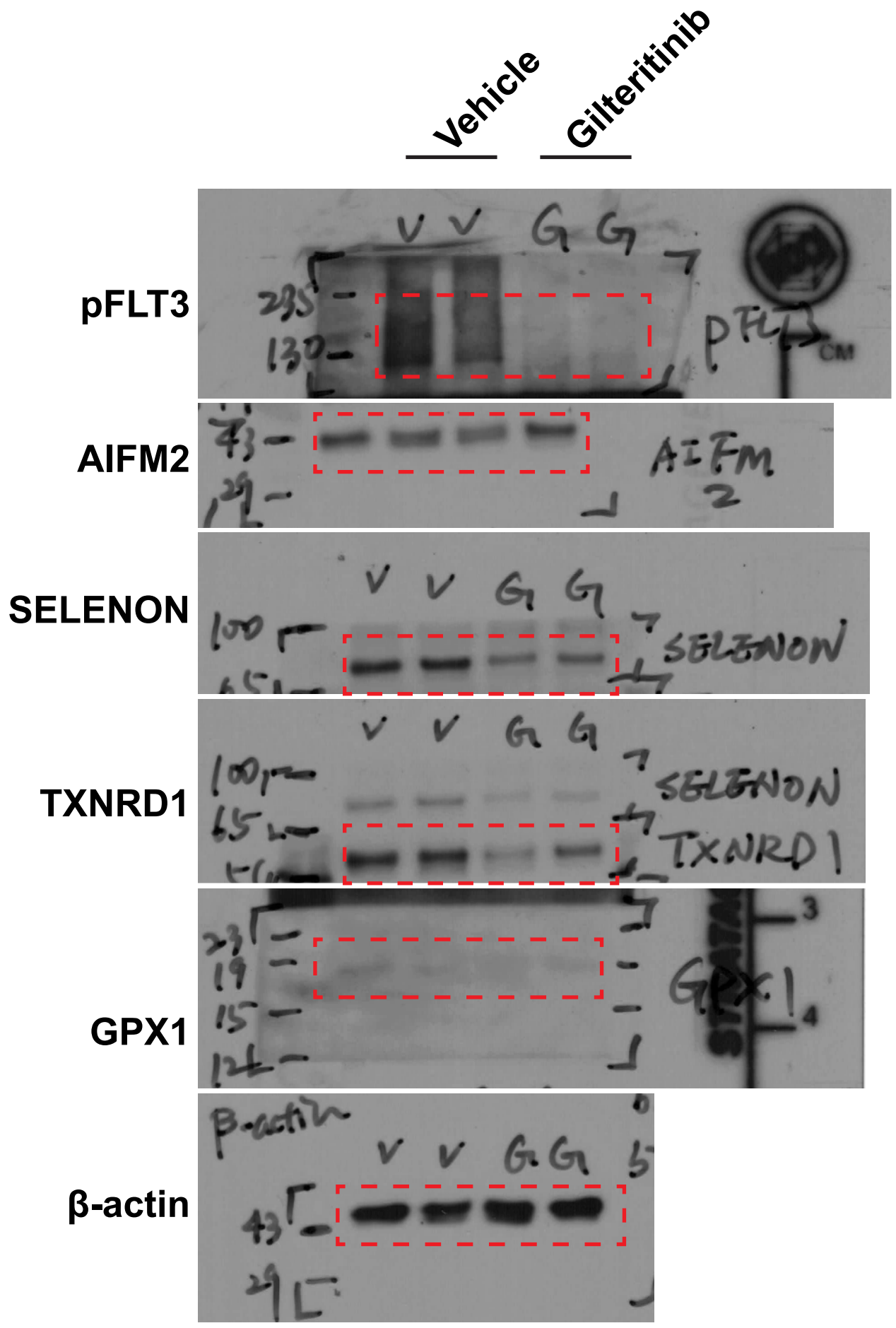

Vav1-NUP98-HOXD13; FLT3$^{ITD/+}$

Ext. Data. Fig. 7I

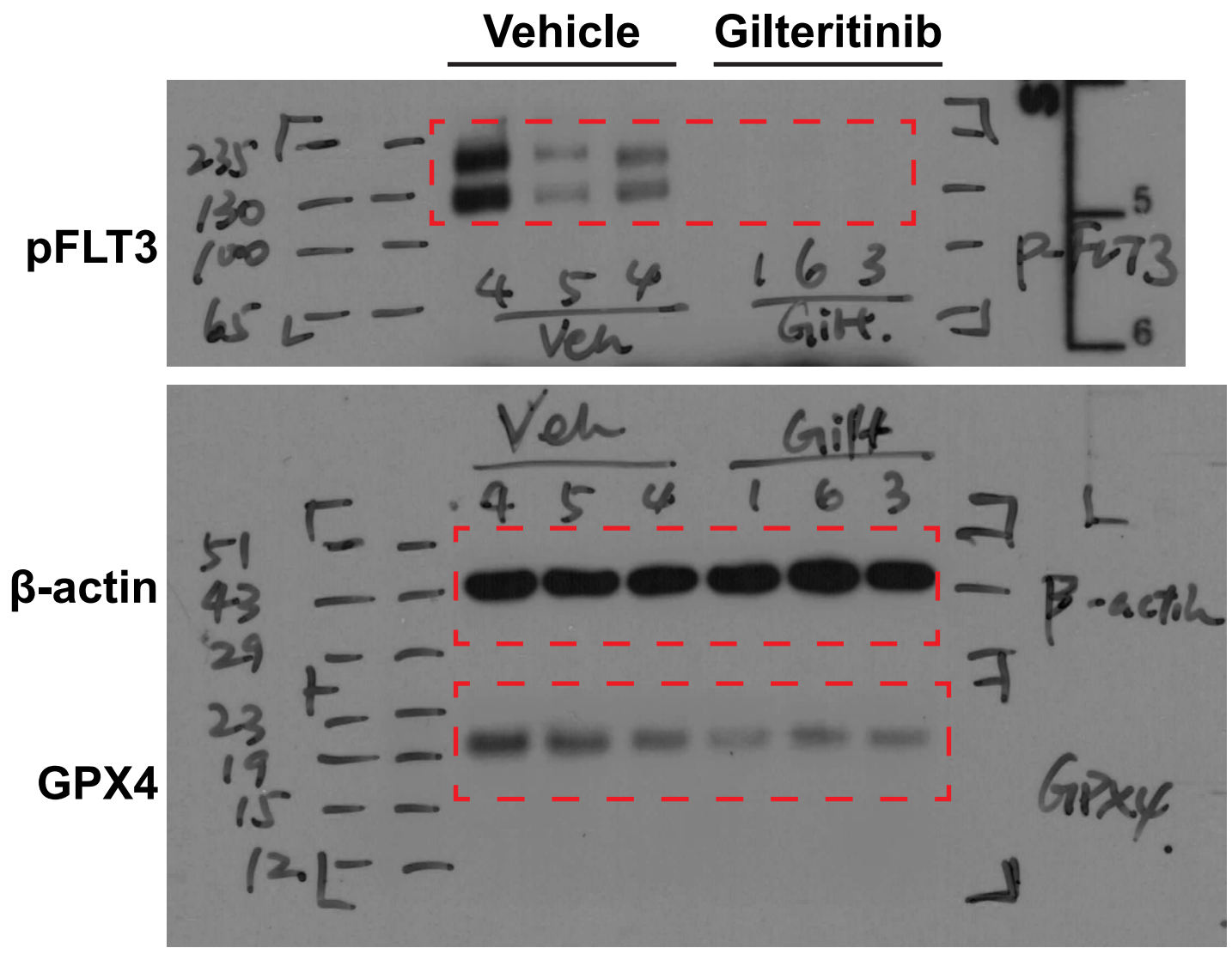

MLL-AF9; FLT3-ITD
