# Supplementary material for: Targeting oncogenic FLT3 uncovers a ferroptosis vulnerability through selenocysteine recoding in acute myeloid leukaemia
Source: Nat Cell Biol. 2026 Aug 7;28(8):1715–27. doi: 10.1038/s41556-026-02016-5 (PMC13457094; doi:10.1038/s41556-026-02016-5)
Supplement: Supplementary file 11 [file 41556_2026_2016_MOESM2_ESM.pdf]

nature portfolio

Corresponding author(s): Daisuke Nakada

Last updated by author(s): 6/5/2026

Reporting Summary

Nature Portfolio wishes to improve the reproducibility of the work that we publish. This form provides structure for consistency and transparency in reporting. For further information on Nature Portfolio policies, see our Editorial Policies and the Editorial Policy Checklist.

Statistics

For all statistical analyses, confirm that the following items are present in the figure legend, table legend, main text, or Methods section.

n/a	Confirmed	
☐	☒	The exact sample size (*n*) for each experimental group/condition, given as a discrete number and unit of measurement
☐	☒	A statement on whether measurements were taken from distinct samples or whether the same sample was measured repeatedly
☐	☒	The statistical test(s) used AND whether they are one- or two-sided<br>*Only common tests should be described solely by name; describe more complex techniques in the Methods section.*
☐	☒	A description of all covariates tested
☐	☒	A description of any assumptions or corrections, such as tests of normality and adjustment for multiple comparisons
☐	☒	A full description of the statistical parameters including central tendency (e.g. means) or other basic estimates (e.g. regression coefficient) AND variation (e.g. standard deviation) or associated estimates of uncertainty (e.g. confidence intervals)
☐	☒	For null hypothesis testing, the test statistic (e.g. *F*, *t*, *r*) with confidence intervals, effect sizes, degrees of freedom and *P* value noted<br>*Give P values as exact values whenever suitable.*
☒	☐	For Bayesian analysis, information on the choice of priors and Markov chain Monte Carlo settings
☒	☐	For hierarchical and complex designs, identification of the appropriate level for tests and full reporting of outcomes
☒	☐	Estimates of effect sizes (e.g. Cohen's *d*, Pearson's *r*), indicating how they were calculated

Our web collection on statistics for biologists contains articles on many of the points above.

Software and code

Policy information about availability of computer code

Data collection	ViiA™ 7 Software, FACSdiva v8.0.1 (BD Biosciences)
Data analysis	Flow cytometry data were analyzed using FlowJo v10.10 (LLC) and FACSdiva v8.0.1 (BD Biosciences). Graphpad Prism v10 (Graphpad Software) was used for data analyses and visualization. RIP-seq data analyzed using R version 4.4.3 (2025-02-28).

For manuscripts utilizing custom algorithms or software that are central to the research but not yet described in published literature, software must be made available to editors and reviewers. We strongly encourage code deposition in a community repository (e.g. GitHub). See the Nature Portfolio guidelines for submitting code & software for further information.

Data

Policy information about availability of data

All manuscripts must include a data availability statement. This statement should provide the following information, where applicable:

- Accession codes, unique identifiers, or web links for publicly available datasets
- A description of any restrictions on data availability
- For clinical datasets or third party data, please ensure that the statement adheres to our policy

Source data are provided with this paper. High throughput screen (HTS) Z-score values are provided in Supplementary Table 1. gRNA sequences and primers used in this study are listed in Supplementary Table 2. GPX4-fl , Vav-iCre, and Rosa26CreERT mice are available from JAX.

Research involving human participants, their data, or biological material

Policy information about studies with human participants or human data. See also policy information about sex, gender (identity/presentation), and sexual orientation and race, ethnicity and racism.

Reporting on sex and gender	The AML PDX model was a gift from Dr. Marina Konopleva and was derived from a 66-year-old male refractory AML patient that received prior treatments with azacitidine and midostaurin. This PDX model was collected by the original investigators under institutional review board approval with written informed consent from the patient.
Reporting on race, ethnicity, or other socially relevant groupings	N/A
Population characteristics	*Describe the covariate-relevant population characteristics of the human research participants (e.g. age, genotypic information, past and current diagnosis and treatment categories). If you filled out the behavioural & social sciences study design questions and have nothing to add here, write "See above."*
Recruitment	This study only used this PDX model and did not involve new collection of human specimens.
Ethics oversight	MD Anderson Cancer Center

Note that full information on the approval of the study protocol must also be provided in the manuscript.

Field-specific reporting

Please select the one below that is the best fit for your research. If you are not sure, read the appropriate sections before making your selection.

☒ Life sciences ☐ Behavioural & social sciences ☐ Ecological, evolutionary & environmental sciences

For a reference copy of the document with all sections, see nature.com/documents/nr-reporting-summary-flat.pdf

Life sciences study design

All studies must disclose on these points even when the disclosure is negative.

Sample size	No methods were used to pre-determine sample size. Sample sizes were chosen based on observed effect sizes and standard errors from previous experiments.
Data exclusions	No data were excluded.
Replication	The reported findings were replicated at least three independent experiments conducted under similar conditions.
Randomization	The assignment of mice to experimental groups was randomized using computer-generated random numbers. This method was used to mitigate bias and ensure that any observed treatment effects could be reliably attributed to the experimental interventions.
Blinding	The investigators were not blinded to allocation during experiments and outcome assessment.

Reporting for specific materials, systems and methods

We require information from authors about some types of materials, experimental systems and methods used in many studies. Here, indicate whether each material, system or method listed is relevant to your study. If you are not sure if a list item applies to your research, read the appropriate section before selecting a response.

Materials & experimental systems

n/a	Involved in the study	
☐	☒	Antibodies
☐	☒	Eukaryotic cell lines
☒	☐	Palaeontology and archaeology
☐	☒	Animals and other organisms
☒	☐	Clinical data
☒	☐	Dual use research of concern
☒	☐	Plants

Methods

n/a	Involved in the study	
☒	☐	ChIP-seq
☐	☒	Flow cytometry
☒	☐	MRI-based neuroimaging

Antibodies

Antibodies used

Flow antibodies: Anti-Mouse: CD2 (PE conjugated, clone RM2-5, Cat# 100108; RRID: AB_2073690); CD2 (BV421 conjugated, clone RM2-5, Cat# 740010; RRID: AB_2739782); CD3 (PE conjugated, clone 145-2C11, Cat# 100308; RRID: AB_312673); CD3 (BV421 conjugated, clone 145-2C11, Cat# 100336; RRID: AB_11203705); CD3 (APC conjugated, clone 145-2C11, Cat# 100312; RRID: AB_312677); CD3 (Biotin conjugated, clone 145-2C11, Cat# 100304; RRID: AB_312669); CD8 (PE conjugated, clone 53-6.7, Cat# 100708; RRID: AB_312747); CD8 (BV421 conjugated, clone 53-6.7, Cat# 100738; RRID: AB_11204079); B220 (PE conjugated, clone RA3-6B2, Cat# 103208; RRID: AB_312993); B220 (BV421 conjugated, clone RA3-6B2, Cat# 103240; RRID: AB_11203896); B220 (PerCP/Cyanine5.5 conjugated, clone RA3-6B2, Cat# 103236; RRID: AB_893354); B220 (APC/Cyanine7 conjugated, clone RA3-6B2, Cat# 103224; RRID: AB_313007); B220 (Alexa Fluor® F700 conjugated, clone RA3-6B2, Cat# 103232; RRID: AB_493717); Ter119 (PE conjugated, clone TER-119, Cat# 116208; RRID: AB_313709); Ter119 (BV421 conjugated, clone TER-119, Cat# 116233; RRID: AB_10933426); Gr-1 (PE conjugated, clone RB6-8C5, Cat# 108408; RRID: AB_313373); Gr-1 (BV421 conjugated, clone RB6-8C5, Cat# 108434; RRID: AB_2562219); Gr-1 (PE/Cyanine7 conjugated, clone RB6-8C5, Cat# 108416; RRID: AB_313381); c-kit/CD117 (APC/Cyanine7 conjugated, clone 2B8, Cat# 105826; RRID: AB_1626278); c-kit/CD117 (Biotin conjugated, clone 2B8, Cat# 13-1171-82; RRID: AB_466569); Sca-1 (APC conjugated, clone D7, Cat# 122512; RRID: AB_756197); Sca-1 (BV785 conjugated, clone D7, Cat# 108139; RRID: AB_2565957); Sca-1 (PE/Cyanine5 conjugated, clone D7, Cat# 108110; RRID: AB_313347); Sca-1 (APC conjugated, clone D7, Cat# 108110; RRID: AB_313347); CD48 (PE/Cyanine7 conjugated, clone HM48-1, Cat# 103424; RRID: AB_2075049); CD48 (Alexa Fluor® F700 conjugated, clone HM48-1, Cat# 103426; RRID: AB_10612755); CD150 (PE/Cyanine5 , clone TC15-12F12.2, Cat# 115912; RRID: AB_493598); Flk2/CD135 (Biotin conjugated, clone A2F10, Cat# 135307; RRID: AB_1953267); IL-7Rα/CD127 (Biotin conjugated, clone A7R34, Cat# 135006; RRID: AB_2126118); CD16/32 (PE/Cyanine7 conjugated, clone 93, Cat# 25-0161-82; RRID: AB_469598); CD16/32 (BV510 conjugated, clone 93, Cat# 101333; RRID: AB_2563692); IL-7Rα/CD127 (PE/Cyanine7 conjugated, clone A7R34, Cat# 135014; RRID: AB_1937265); CD11b (APC/Cyanine7 conjugated, clone M1/70, Cat# ; RRID:); CD45.1 (APC/Cyanine7 conjugated, clone A20, Cat# 110716; RRID: AB_313505); CD45.2 (APC conjugated, clone 104, Cat# 56-0454-82; RRID: AB_657752); CD34 (eFluor660 conjugated, clone RAM34, Cat# 50-0341-82; RRID: AB_10596826); CD34 (Biotin conjugated, clone RAM34, Cat# 13034181; RRID: AB_466424)
Anti-Human antibodies: CD45 (APC conjugated, clone HI30, Cat# 304012; RRID: AB_314400)

Antibodies used for immunoblotting: EEFSEC (1:1000, 10628-1-AP, Proteintech); GPX4 (1:2500, ab125066, Abcam); Phospho-FLT3 (1:1000, 3464S, Cell Signaling Technology); FLT3 (1:1000, 3462S, Cell Signaling Technology); SEPHS2 (1:1000, PA5-27950, Invitrogen); TXNRD1 (1:1000, 11117-1-AP, Proteintech); MSRB1/SEPX1 (1:1000, 15333-1-AP, Proteintech); GPX1 (1:1000, MA5-45018, Invitrogen); FSP1 / AIFM2 (1:1000, 20886-1-AP, Proteintech); p-p44/42 (1:1000, 4370S, Cell Signaling Technology); p44/42 (1:1000, 4695S, Cell Signaling Technology); MEK1 (1:1000, 9124S, Cell Signaling Technology); p-c-ABL (1:1000, 2865S, Cell Signaling Technology); c-ABL (1:1000, 2862S, Cell Signaling Technology); LRP8 (1:1000, MA5-38208, Invitrogen); anti-Ty1 (1:1000, MA5-23513, ThermoFisher Scientific); anti-GAPDH (1:5000, 2118, Cell Signaling Technology); anti-beta-actin (1:5000, A5441, Sigma Aldrich); SLC7A11 (1:1000, #12691S, Cell Signaling Technology); SELENOP (1:1000, ab109514, Abcam); phospho-MAPK Substrates (1:1000, CST14378S, Cell Signaling Technology)

Validation

Primary antibodies were validated and titrated with appropriate positive and negative controls in order to determine the optimal stain concentration for each test. Flow antibodies were confirmed using isotype controls.

Eukaryotic cell lines

Policy information about cell lines and Sex and Gender in Research

Cell line source(s)

No ICLAC cell lines were used in this study. HEK293T (ATCC, CRL-3216; RRID: CVCL_0063) was from ATCC. MOLM-13, MV4-11, THP-1, NB-4, K562, Kasumi-1, KG-1, HL-60, OCI-AML3 are gifts from laboratories of Margaret Goodell, Rachel Rau and Daniel Lacorazza at Baylor College of Medicine. Murine leukemia cells were isolated from bone marrow of AML mice induced by MLL-AF9 oncogene with or without FLT-ITD (or other FLT3-ITD variants). Transgenic Vav1-NUP98-HOXD13; Flt3ITD/+ AML cells were isolated from bone marrow of primary AML mice.

Authentication

These cell lines were authenticated by Cytogenetic and Cell Authentication Core at MD Anderson Cancer Center.

Mycoplasma contamination

The cell lines were routinely tested free of mycoplasma contamination.

Commonly misidentified lines
(See ICLAC register)

No ICLAC cell lines were used in this study.

Animals and other research organisms

Policy information about studies involving animals; ARRIVE guidelines recommended for reporting animal research, and Sex and Gender in Research

Laboratory animals

C57BL/6J (JAX stock: 00664), B6.SJL-Ptprca Pepcb/BoyJ (CD45.1) (JAX stock: 002014), Gpx4tm1.1Qra/J (JAX stock: 027964), B6.Cg-Commd10Tg(Vav1-icre)A2Kio/J (JAX stock: 008610), B6.129-Gt(ROSA)26Sortm1(cre/ERT2)Tyj/J (JAX stock: 008463). The mice were housed in the Association for Assessment and Accreditation of Laboratory Animal Care International (AAALAC)-accredited, specific pathogen-free animal care facilities at Baylor College of Medicine (BCM).

Wild animals

N/A

Reporting on sex

Both sexes were used in the study.

Field-collected samples	N/A
Ethics oversight	All animal experiments were performed in accordance with protocols approved by the Institutional Animal Care and Use Committee (IACUC) of BCM.

Note that full information on the approval of the study protocol must also be provided in the manuscript.

Plants

Seed stocks	*Report on the source of all seed stocks or other plant material used. If applicable, state the seed stock centre and catalogue number. If plant specimens were collected from the field, describe the collection location, date and sampling procedures.*
Novel plant genotypes	*Describe the methods by which all novel plant genotypes were produced. This includes those generated by transgenic approaches, gene editing, chemical/radiation-based mutagenesis and hybridization. For transgenic lines, describe the transformation method, the number of independent lines analyzed and the generation upon which experiments were performed. For gene-edited lines, describe the editor used, the endogenous sequence targeted for editing, the targeting guide RNA sequence (if applicable) and how the editor was applied.*
Authentication	*Describe any authentication procedures for each seed stock used or novel genotype generated. Describe any experiments used to assess the effect of a mutation and, where applicable, how potential secondary effects (e.g. second site T-DNA insertions, mosiacism, off-target gene editing) were examined.*

Flow Cytometry

Plots

Confirm that:

☒ The axis labels state the marker and fluorochrome used (e.g. CD4-FITC).

☒ The axis scales are clearly visible. Include numbers along axes only for bottom left plot of group (a 'group' is an analysis of identical markers).

☒ All plots are contour plots with outliers or pseudocolor plots.

☒ A numerical value for number of cells or percentage (with statistics) is provided.

Methodology

Sample preparation	Peripheral blood, whole bone marrow isolated from tibias and femurs, and other mouse organs were used for flow cytometry analysis. Peripheral blood samples were incubated with ACK solution to lyse red blood cells. The whole bone marrow cells were filtered through a 48um cell strainer to obtain single cell suspension. These samples were subsequently stained with flow cytometry antibodies, washed, and analyzed with a flow cytometer.
Instrument	Flow cytometry was performed with FACSAria II, FACSCanto II, LSR II, or LSRFortessa flow cytometers (BD Biosciences), and cell sorting with a FACS Aria III.
Software	Data analysis was performed using FlowJo v10.7 and FACS DIVA 8.0.1.
Cell population abundance	The cell population abundance was shown in the flow cytometry plots and source data.
Gating strategy	HSCs (lineage−Sca-1+c-kit+(LSK) CD150+CD48−/low), MPPs (LSKCD150-CD48-/low), HPC2 (LSKCD150+CD48+), HPC1 (LSKCD150-CD48+), GMP (Lin-Sca1-c-kit+CD34+CD16/32+), CMP (Lin-Sca1-c-kit+CD34+CD16/32-/low), MEP (Lin-Sca1-c-kit +CD34-/lowCD16/32-/low), CLP (Lin-Sca1lowc-kit+IL7R+Flk2+) and LSCs (GFP+Lin-Sca1-c-kit+CD34+CD16/32+ or GFP/ mCherry+Lin-Sca1-c-kit+CD34+CD16/32+ or CD45.2/mCherry+Lin-Sca1-c-kit+CD34+CD16/32+)

☒ Tick this box to confirm that a figure exemplifying the gating strategy is provided in the Supplementary Information.
