# Supplementary material for: Targeting oncogenic FLT3 uncovers a ferroptosis vulnerability through selenocysteine recoding in acute myeloid leukaemia
Source: Nat Cell Biol. 2026 Aug 7;28(8):1715–27. doi: 10.1038/s41556-026-02016-5 (PMC13457094; doi:10.1038/s41556-026-02016-5)
Supplement: Supplementary file 13 [file 41556_2026_2016_MOESM7_ESM.pdf]

Figure 3

Fig. 3B

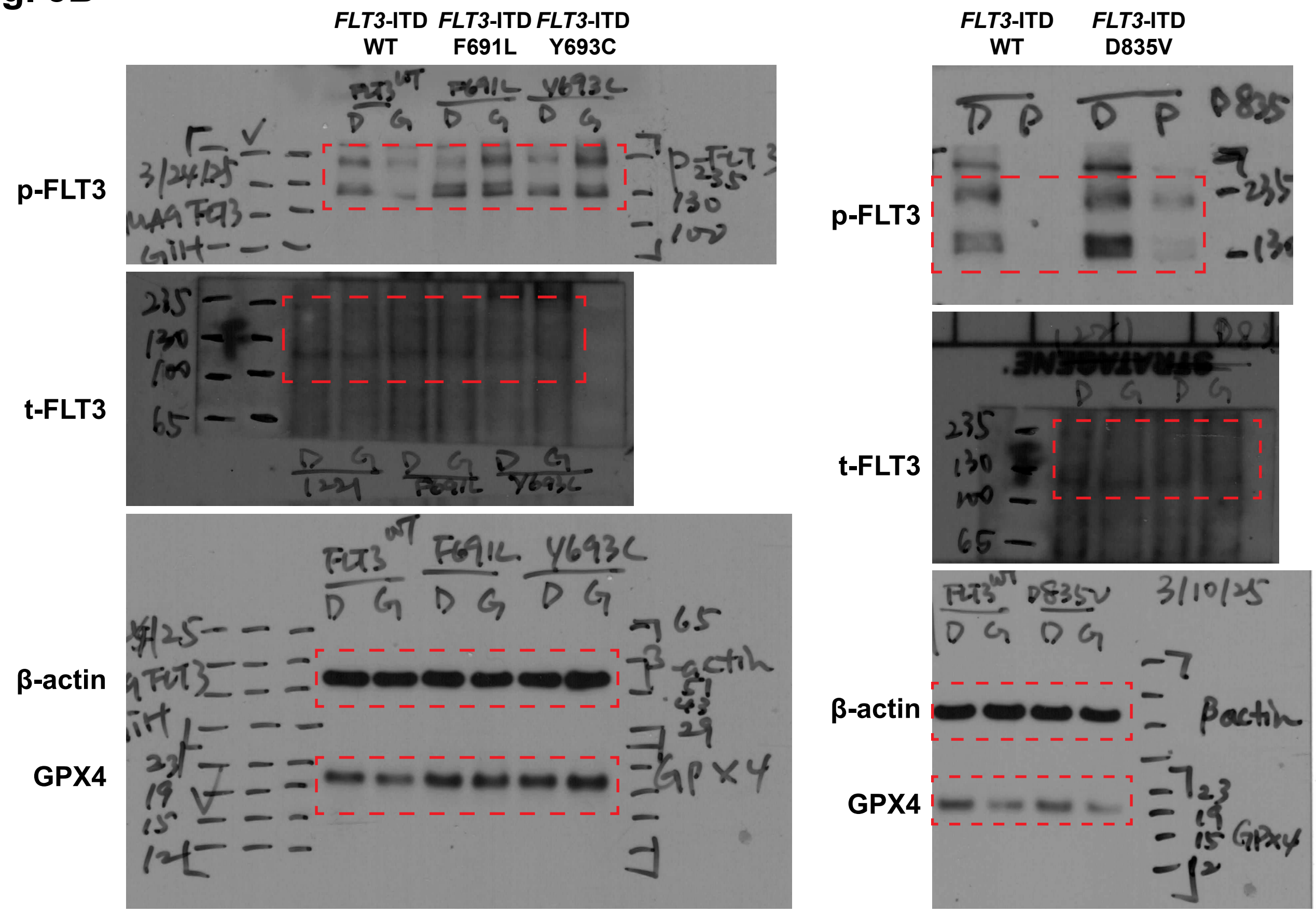

D= DMSO treated
G= Gilteritinib treated

D= DMSO treated
G= Gilteritinib treated

Fig. 3C

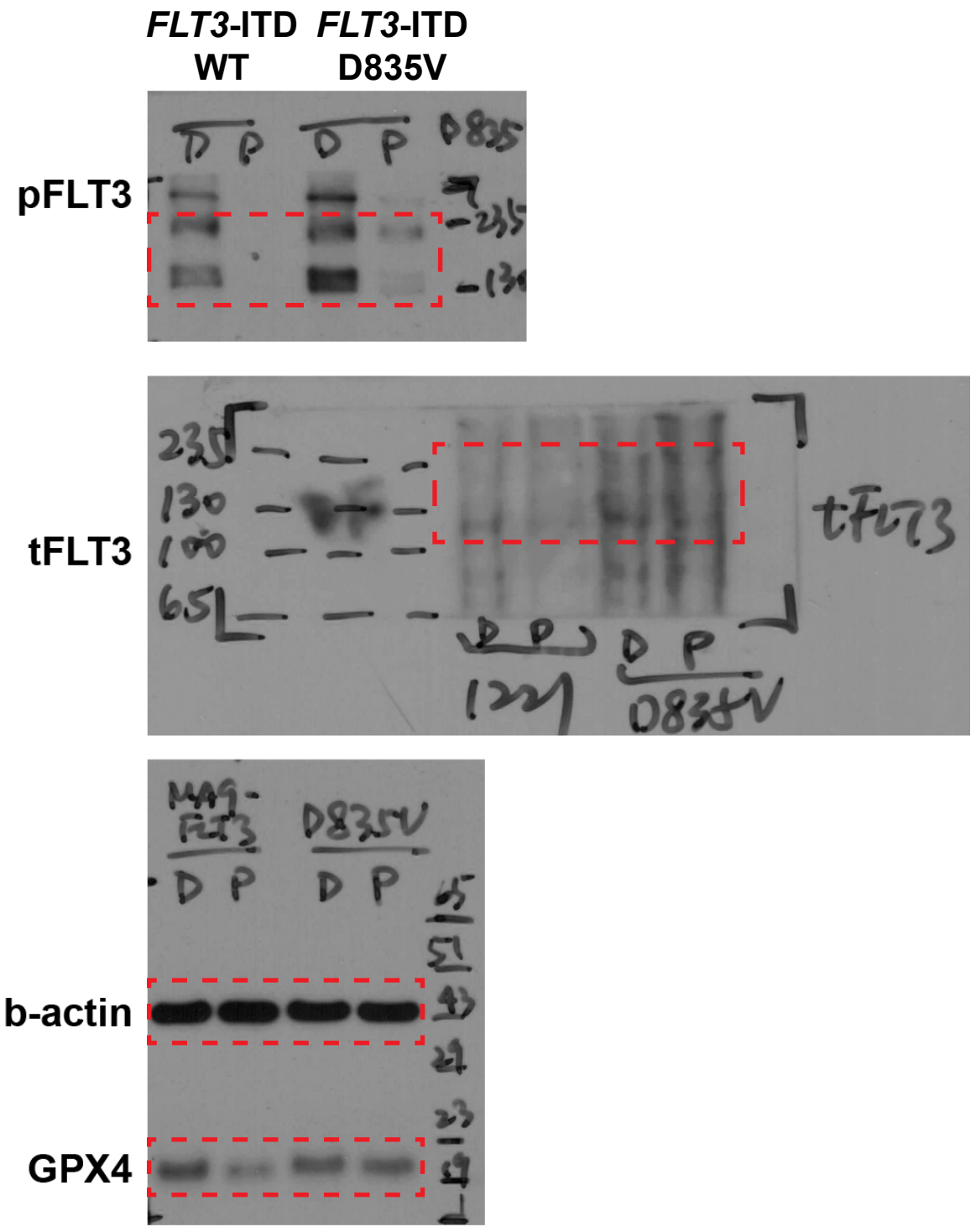

D= DMSO treated
P= Ponatinib treated
